# Drug-resistant enteric fever worldwide, 1990 to 2018: a systematic review and meta-analysis

**DOI:** 10.1186/s12916-019-1443-1

**Published:** 2020-01-03

**Authors:** Annie J. Browne, Bahar H. Kashef Hamadani, Emmanuelle A. P. Kumaran, Puja Rao, Joshua Longbottom, Eli Harriss, Catrin E. Moore, Susanna Dunachie, Buddha Basnyat, Stephen Baker, Alan D. Lopez, Nicholas P. J. Day, Simon I. Hay, Christiane Dolecek

**Affiliations:** 1 Big Data Institute, Li Ka Shing Centre for Health Information and Discovery, University of Oxford, Oxford, UK; 2 Institute for Health Metrics and Evaluation, University of Washington, Seattle, WA USA; 3 Bodleian Health Care Libraries, University of Oxford, Oxford, UK; 4 Centre for Tropical Medicine and Global Health, Nuffield Department of Medicine, University of Oxford, Oxford, UK; 5 Mahidol-Oxford Tropical Medicine Research Unit, Faculty of Tropical Medicine, Mahidol University, Bangkok, Thailand; 6 Oxford University Clinical Research Unit Nepal, Patan Academy of Health Sciences, Kathmandu, Nepal; 7 Oxford University Clinical Research Unit Vietnam, The Hospital for Tropical Diseases, Wellcome Trust Major Overseas Programme, Ho Chi Minh City, Vietnam; 8 Melbourne School of Population and Global Health, University of Melbourne, Melbourne, Australia; 9 Department of Health Metrics Sciences, School of Medicine, University of Washington, Seattle, USA

**Keywords:** Enteric fever, Typhoid fever, Paratyphoid fever, *Salmonella* Typhi, *Salmonella* Paratyphi A, Antimicrobial drug resistance, Harmonisation of breakpoints, Prevalence of resistance, Meta-analysis, Drug-resistant infections, Multidrug resistance, Fluoroquinolone resistance, Ceftriaxone resistance, ESBL-producing, Azithromycin resistance

## Abstract

**Background:**

Antimicrobial resistance (AMR) is an increasing threat to global health. There are > 14 million cases of enteric fever every year and > 135,000 deaths. The disease is primarily controlled by antimicrobial treatment, but this is becoming increasingly difficult due to AMR. Our objectives were to assess the prevalence and geographic distribution of AMR in *Salmonella enterica* serovars Typhi and Paratyphi A infections globally, to evaluate the extent of the problem, and to facilitate the creation of geospatial maps of AMR prevalence to help targeted public health intervention.

**Methods:**

We performed a systematic review of the literature by searching seven databases for studies published between 1990 and 2018. We recategorised isolates to allow the analysis of fluoroquinolone resistance trends over the study period. The prevalence of multidrug resistance (MDR) and fluoroquinolone non-susceptibility (FQNS) in individual studies was illustrated by forest plots, and a random effects meta-analysis was performed, stratified by Global Burden of Disease (GBD) region and 5-year time period. Heterogeneity was assessed using the *I*^2^ statistics. We present a descriptive analysis of ceftriaxone and azithromycin resistance.

**Findings:**

We identified 4557 articles, of which 384, comprising 124,347 isolates (94,616 *S*. Typhi and 29,731 *S*. Paratyphi A) met the pre-specified inclusion criteria. The majority (276/384; 72%) of studies were from South Asia; 40 (10%) articles were identified from Sub-Saharan Africa. With the exception of MDR *S*. Typhi in South Asia, which declined between 1990 and 2018, and MDR *S*. Paratyphi A, which remained at low levels, resistance trends worsened for all antimicrobials in all regions. We identified several data gaps in Africa and the Middle East. Incomplete reporting of antimicrobial susceptibility testing (AST) and lack of quality assurance were identified.

**Interpretation:**

Drug-resistant enteric fever is widespread in low- and middle-income countries, and the situation is worsening. It is essential that public health and clinical measures, which include improvements in water quality and sanitation, the deployment of *S*. Typhi vaccination, and an informed choice of treatment are implemented. However, there is no licenced vaccine for *S*. Paratyphi A. The standardised reporting of AST data and rollout of external quality control assessment are urgently needed to facilitate evidence-based policy and practice.

**Trial registration:**

PROSPERO CRD42018029432.

## Background

Enteric fever, a serious bloodstream infection caused by the human-restricted bacterial pathogens *Salmonella enterica* serovars Typhi (*S.* Typhi) and Paratyphi A, is an important cause of morbidity and mortality in the developing world. Transmission occurs faeco-orally through contaminated water and food. An estimated 14.3 million infections and more than 135,000 deaths are caused by enteric fever worldwide each year [1], mostly affecting children and young adults.

*S.* Typhi is the aetiological agent of almost 30% of community-acquired bacterial bloodstream infections in Asia [2] and 10% in Africa [3], whilst *S.* Paratyphi A is an emerging pathogen in Asia, that causes up to 35% of all enteric fever episodes in India and Nepal and more than 60% in China [4, 5]. Notably, paratyphoid fever is clinically indistinguishable from typhoid fever [4]. Enteric fever is an important cause of acute undifferentiated febrile illness [6]. There is heterogeneity in the aetiologies of febrile illness according to geographic location, age group, diagnostic testing panel and seasonality [6–8]. A study in India identified enteric fever in 4% of > 1200 adult patients as the cause of febrile illness (the testing panel included dengue fever, scrub typhus, leptospirosis, enteric fever and malaria) [9], whilst a study in Nepal that tested for bacterial pathogens, dengue and HIV reported enteric fever in 36% (117/323) of febrile illnesses with confirmed bacterial aetiology [10], highlighting this variation.

Enteric fever has been eliminated in industrialised countries by improving drinking water and sanitation; vaccination can also be deployed to reduce the burden of typhoid fever (there is no vaccine against *S*. Paratyphi A), but effective treatment is critical to reduce morbidity and mortality. However, the development and spread of antimicrobial drug resistance (AMR) threatens the effectiveness of antimicrobials and may lead to a resurgence of enteric fever in many parts of the world. As is true for many bacterial infections, there is no simple and reliable point-of-care test that can diagnose enteric fever, define the antimicrobial susceptibility profile and inform patient management. The gold standard diagnostic, microbiological blood culture, is expensive and slow (it usually takes 3–4 days to get the blood culture and susceptibility testing result) and has a low sensitivity of approximately 50% [11, 12], due to the low-grade bacteraemia [13]. Prior to the first antimicrobials, case fatality rates were approximately 30%; this has been reduced to less than 1%, depending on the timely initiation of the appropriate empirical treatment [14]. Antimicrobial susceptibility testing (AST) and surveillance play a critical role in capturing local susceptibility patterns and guiding empirical treatment; however, microbiological facilities and the relevant expert knowledge are lacking in many low- and middle-income countries (LMICs) [15–17]. Significantly, *S*. Typhi and *S*. Paratyphi are WHO priority pathogens for AMR surveillance [17].

The WHO currently recommends chloramphenicol, ampicillin and cotrimoxazole (trimethoprim-sulfamethoxazole), fluoroquinolones, third-generation cephalosporines (ceftriaxone, cefixime) and azithromycin for the treatment of enteric fever [11]. Unfortunately, AMR is widespread, and patients treated with ineffective antimicrobials show a poor clinical response and a higher rate of complications and deaths, as well as prolonged faecal shedding, which sustains transmission and induces secondary cases [18–20].

Here, we performed a systematic review and meta-analysis of the literature to evaluate the prevalence of AMR in *S.* Typhi and *S*. Paratyphi A and to determine the spatial and temporal distribution of drug-resistant enteric fever at the regional level, grouped by Global Burden of Disease (GBD) study region from 1990 to 2018. The ultimate aim of our work is to create fine-scaled geospatial maps of the distribution of AMR to aid targeted public health interventions for this preventable disease [21].

## Methods

### Search strategy and selection criteria

We conducted a systematic review of published literature between 1990 and 2018 following the PRISMA guidelines (Additional file 1: Table S1) [22]. The protocol was registered with the international prospective register of systematic reviews (CRD42018029432). The search strategy was devised by an academic librarian (EH). MEDLINE, Ovid Embase, Global Health, Cochrane Library, Scopus, Web of Science-Core Collection and LILACS were searched using a syntax that combined Medical Subject Headings (MeSH) and free text terms for the pathogens of interest (e.g. *S.* Typhi, *S.* Paratyphi A, enteric fever) with terms for antimicrobial resistance (e.g. resistan*, suscept*, surveil*) (Additional file 1: Table S2). The extended search was conducted in October 2017 and updated in March 2019. The search was limited to publications from 1990 onwards; no restrictions on language or filters (e.g. humans) were implemented.

Included studies were required to report quantifiable in vitro antimicrobial susceptibility data for *S.* Typhi and/or *S.* Paratyphi A isolated from blood culture, examining at least 10 representative organisms and indicating the study location. Reports from travellers being diagnosed in high-income countries were excluded. Studies with pooled *S.* Typhi and *S.* Paratyphi A susceptibility data, studies reporting on isolates from stool culture and duplicate isolates were also excluded.

Prospective and retrospective hospital-, laboratory- and community-based studies were included, if they met the specified inclusion criteria. Review articles were scanned for relevant references*.* Studies were screened at title, abstract and full-text stage by one author (CD) and reviewed by a second author (AB). Data were extracted into a predefined database by AB and reviewed by BKH and JL. Additionally, 20% of the extracted studies were checked by a third reviewer (CD). Disagreements were resolved by discussion. Susceptibility data for antimicrobials recommended for the treatment of enteric fever by WHO, i.e. ampicillin/amoxicillin, chloramphenicol, trimethoprim-sulphamethoxazole (co-trimoxazole), fluoroquinolones (e.g. ciprofloxacin and ofloxacin), third-generation cephalosporins (e.g. ceftriaxone and cefixime) and azithromycin, were extracted [11]. Furthermore, multidrug resistance (MDR; defined as resistance to ampicillin/amoxicillin, chloramphenicol and co-trimoxazole) and nalidixic acid resistance, as a proxy marker for reduced ciprofloxacin susceptibility, were recorded [18].

Variables extracted included the study start and end dates, patients’ characteristics (age range, mean age, percentage of males, inpatients or outpatients), study design, number of patients screened, number of patients with positive blood culture, antimicrobial susceptibility testing (AST) method and the number (or percentage) of resistant, intermediate and susceptible isolates out of the total number of isolates tested against each antimicrobial. We also recorded case fatalities and clinical outcomes when available. Additionally, the testing standard (e.g. Clinical and Laboratory Standards Institute (CLSI)) and interpretive criteria (including version or year) used to determine resistance, use of internal quality controls and participation in external quality assessments schemes were recorded. The study setting, precise study location, country and GBD study region were recorded for each study. Data were disaggregated by serovar and study location.

We aimed to control for bias and allow for comparison across studies by adhering to the predefined inclusion and exclusion criteria. We expected that there would be differences in the quality of the AST and interpretation of results, reflecting the reality in many LMICs. We adapted a descriptive tool for quality assessment used by Arndt, based on sample size and microbiological testing methodology [23]. We reviewed the complete description of susceptibility testing methods, which included testing standard, version and/or year (i.e. breakpoints), internal quality controls and external quality assessment. No study was excluded based on this assessment, due to the lack of standardised reporting guidelines for microbiological studies.

### Data analysis

Each study was assigned to a year based on the midyear of the study. Studies were grouped based on the GBD region and 5-year time period (1990–1994; 1995–1999; 2000–2004; 2005–2009; 2010–2014; 2015–2018). If study dates were not provided, these were imputed as the publication date minus the median difference between the publication date and the mid-year for the remaining studies in the dataset.

Typhoid-specific lower breakpoints against fluoroquinolones (FQ) came into effect during our study period [24]. To allow the analysis of resistance trends over time, we classified ciprofloxacin intermediate (minimum inhibitory concentration (MIC) 0.12–0.5 μg/mL) and resistant *S.* Typhi and *S*. Paratyphi (MIC ≥ 1 μg/mL) according to the updated breakpoints (CLSI, 2012), as well as isolates with ‘decreased ciprofloxacin (or FQ) susceptibility’ (ciprofloxacin MIC 0.125–1.0 μg/mL) and nalidixic acid-resistant isolates (as proxy marker for ‘decreased ciprofloxacin (or fluoroquinolone) susceptibility’), as fluoroquinolone non-susceptible (FQNS). The term ‘decreased ciprofloxacin (or FQ) susceptibility’ described organisms with raised ciprofloxacin MICs that technically were not resistant due to the higher historical FQ breakpoints before 2012. If ciprofloxacin data were not available or it was not clear which breakpoints were used, nalidixic acid resistance data were used instead.

For all other antimicrobials, we classified intermediate susceptible organisms as resistant. We determined the percentage of patients with resistant *S.* Typhi or *S.* Paratyphi A isolates and used forest plots to illustrate the proportion of MDR and FQNS for each individual study; 95% confidence intervals (CI) were calculated using the Agresti-Coull method [25].

We combined individual studies using random effect meta-analysis to arrive at pooled prevalence rates of MDR and FQNS for each region, time period and serovar. Heterogeneity was assessed visually using forest plots and quantitatively using the *I*^2^ statistic and its associated *p* value [26]. In addition to the categorical data on the proportion of FQNS, we present quantitative ciprofloxacin MIC data for *S.* Typhi from large studies with > 90 isolates in Delhi, India. Stacked bar plots were used to illustrate changes in the distribution of ciprofloxacin MICs over the study period.

Ceftriaxone and azithromycin are recommended for the treatment of MDR and FQ-resistant enteric fever [11]. We also provide a descriptive analysis of ceftriaxone and azithromycin resistance as part of this review.

We used double arcsine transformation to stabilise the variance of proportions and performed random effects meta-analysis using the REML heterogeneity variance estimator [27]. Pooled prevalence was calculated for sub-groups that included at least three studies. All statistical analyses were conducted at a 5% significance level using the statistical software package ‘metafor’ in R (version 3.4.2).

## Results

**Fig. 1:**
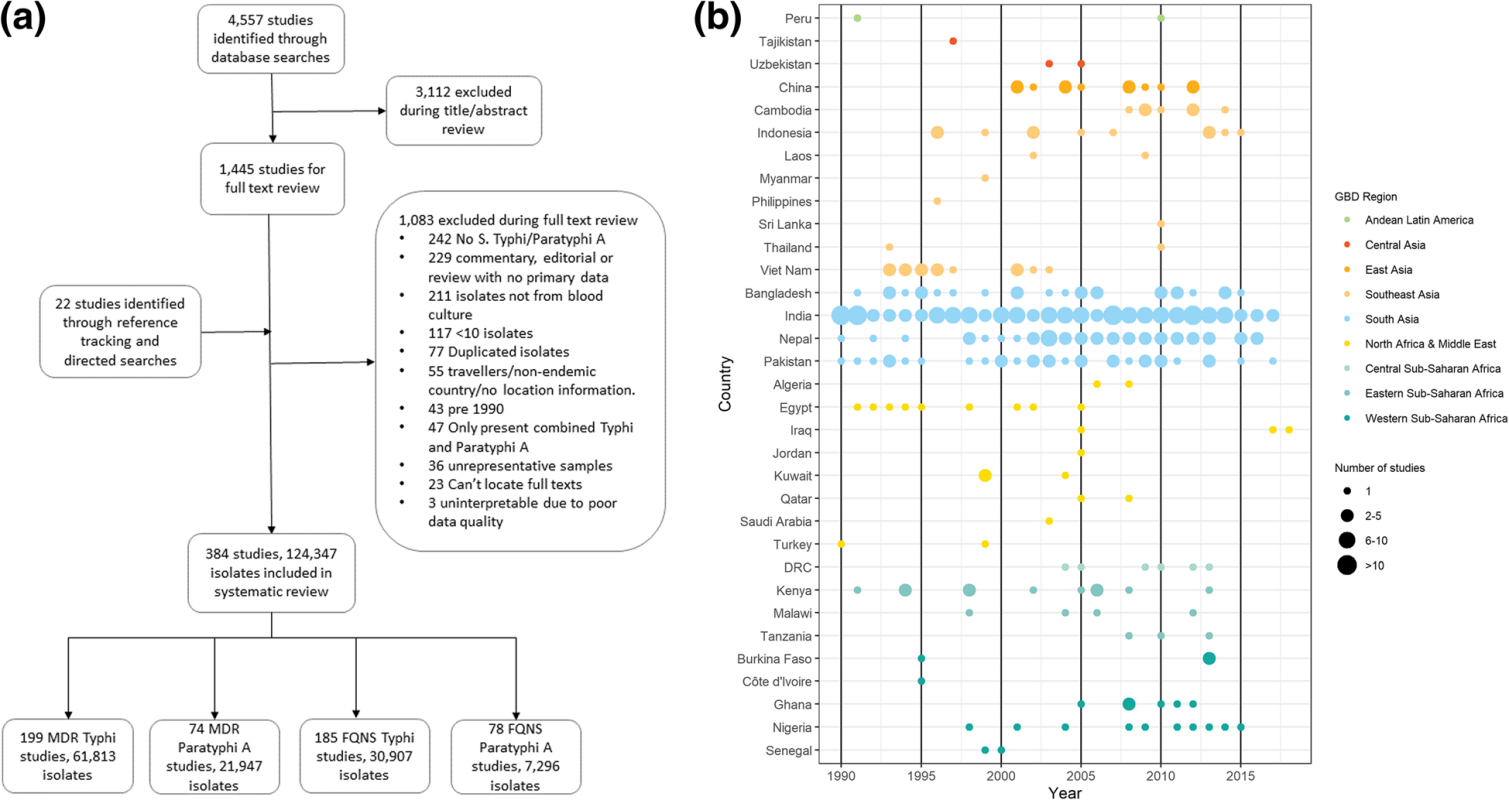
Study selection. **a** PRISMA flow chart depicting study screening and selection; approximately 150 studies were received from the Bodleian Library/British National Library. **b** Data availability plotted by year (*x*-axis) and country (*y*‐axis), grouped by region. The number of studies for each country‐year is depicted by the size of the point

Our online database searches identified 4557 articles, with an additional 22 obtained through reference tracking. A total of 3112 studies were excluded at abstract review and 1445 at full-text review; the main reasons for exclusion are shown in Fig. 1a. Ultimately, data were extracted from 384 articles yielding information for 124,347 isolates: 94,616 *S*. Typhi and 29,731 *S*. Paratyphi A. There were 199 data points for MDR *S.* Typhi, 185 for FQNS *S*. Typhi, 73 data points for MDR *S*. Paratyphi A and 78 for FQNS *S*. Paratyphi A (Fig. 1a, Additional file 1: Table S3)*.* (One study could contribute several data points due to reporting on multiple antimicrobials, serovars and locations.) The majority of data were from South Asia, with the highest number of reports (173/384; 45%) from India. No data were identified from Oceania (Fig. 1b). Table 1 shows the study characteristics.

**Table 1:** Study characteristics – population characteristics

Study characteristics	Number of studies (%)
**Region of study**^a^	
Andean Latin America	2 (0.5)
Central Asia	3 (0.8)
East Asia	12 (3.1)
North Africa & Middle East	17 (4.4)
South Asia	276 (71)
Southeast Asia	38 (9.8)
Sub-Saharan Africa, Central	6 (1.5)
Sub-Saharan Africa, Eastern	14 (3.6)
Sub-Saharan Africa, Western	21 (5.4)
**Number of blood cultures screened**	
0-99	13 (3.4)
100-499	43 (11.2)
500-999	30 (7.8)
1000-4999	49 (12.8)
5000+	59 (15.4)
Not stated	190 (49.5)
**Specific age groups**	
Adults only	5 (1.3)
Children only	68 (17.7)
No specified age restrictions/Adults and children	311 (81)
**Reported pre-admission antibiotic use (proportion of patients in the study)**^b^	
0	19 (4.9)
1-25%	15 (3.8)
26-50%	13 (3.3)
51-75%	7 (1.8)
76-100%	7 (1.8)
Not stated	329 (84.4)
**Reported case fatality rate**	
0	61 (15.9)
1-5%	24 (6.3)
6-10%	2 (0.5)
11-15%	1 (0.3)
16-20%	2 (0.5)
21-25%	1 (0.3)
Not stated	293 (76.3)
**Patient type**^c^	
Inpatients	73 (19)
Outpatients	14 (3.6)
Outpatients & Emergency department	6 (1.6)
Inpatients & Outpatients	44 (11.4)
Community	7 (1.8)
Not specified	241 (62.6)

^a^Three studies reported isolates from multiple regions

^b^Six studies reported the proportion of participants using antibiotics prior to testing separately for different sites or for persons infected with *S.* Typhi and *S.* Paratyphi A separately

^c^One study consisted of two separate parts, one was community based and the other in outpatients

The majority (230/384; 60%) of reports were retrospective studies. AST methods were reported in 329 (86%) studies and were primarily Kirby-Bauer disc diffusion; testing standards (e.g. CLSI) were reported in 218 (57%) studies; interpretive criteria (version or year; i.e. breakpoints) were reported in 168 (44%) studies and use of internal quality controls in 122 (32%) studies*.* Five studies reported participation in international and two studies in national EQA schemes, whilst 23 studies reported confirmation of AST results by national or international reference laboratories (Table 2). Clinical outcomes including case fatalities were presented by 91 studies (Tables 1 and 2).

**Table 2:** Study characteristics – quality assessment

Study quality characteristics	Number of studies (%)
**Study design**^a^	
Clinical trial	16 (4.0)
Prospective	155 (38.7)
Retrospective	230 (57.4)
**Sample size**	
10-29	63 (16.4)
30-99	137 (35.7)
>100	184 (47.9)
**Method of antimicrobial susceptibility testing**	
Disk-diffusion	183 (47.7)
Disk-diffusion & MIC determination	98 (25.5)
Microdilution	26 (6.8)
E-test	9 (2.3)
Automated methods^b^	7 (1.8)
Multiple MIC determination methods	6 (1.6)
Not stated	55 (14.3)
**Guidelines for antimicrobial susceptibility testing**^c^	
BSAC/EUCAST	11 (2.8)
CLSI/NCCLS	200 (51.4)
Other	12 (3.1)
Not stated	166 (42.7)
**Version of antimicrobial susceptibility testing guidelines stated**	
Stated	168 (43.8)
Not stated	216 (56.3)
**Internal quality control reported**	
Yes	122 (31.8)
No	262 (68.2)
**External quality assessment (EQA) participation reported**	
International EQA	5 (1.3)
National EQA	2 (0.5)
Results confirmed by national or international surveillance laboratory	23 (6)
Not stated	354 (92.2)

^a^One article combined the report of a retrospective and a prospective study

^b^Automated systems include VITEK 2, Phoenix 100 and Rapid ATB tests

^c^Six studies used the CLSI/NCCS and the BSAC/EUCAST guidelines for different antibiotics so contributed to the numbers twice

Heterogeneity was high (*I*^2^ > 80%) within most subgroups (Figs. 2, 3, 4 and 5, Additional file 1: Figures S3-S12). Results of our sensitivity analysis (Additional file 1: Tables S4a & b, Figure S2a & b) showed that removing studies deemed as having a risk of bias due to incomplete reporting of AST methodology had no effect on either the heterogeneity within subgroups, or on the pooled prevalence of resistance. This further supported our decision not to exclude studies based on the risk of bias assessment.

**Fig. 2:**
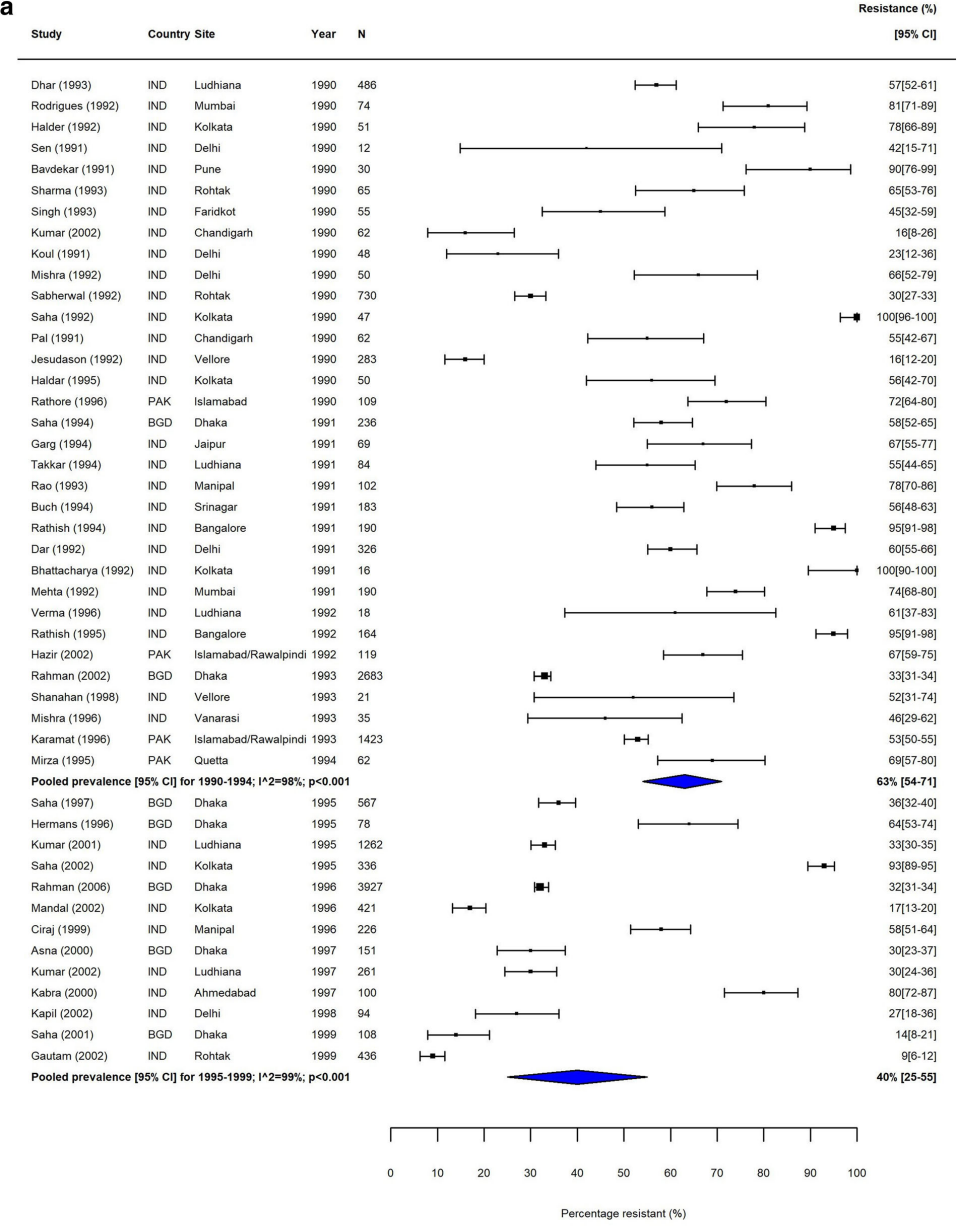

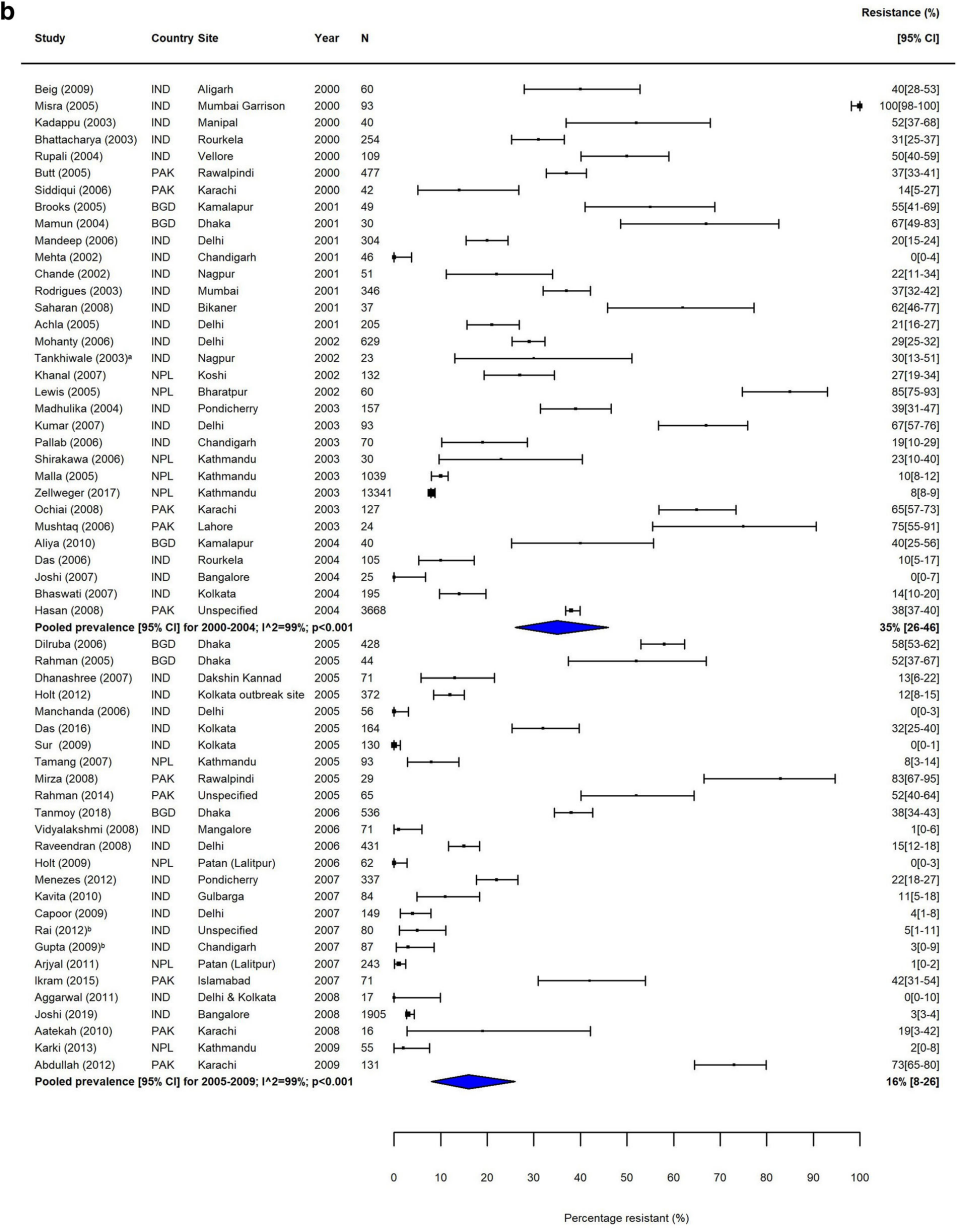

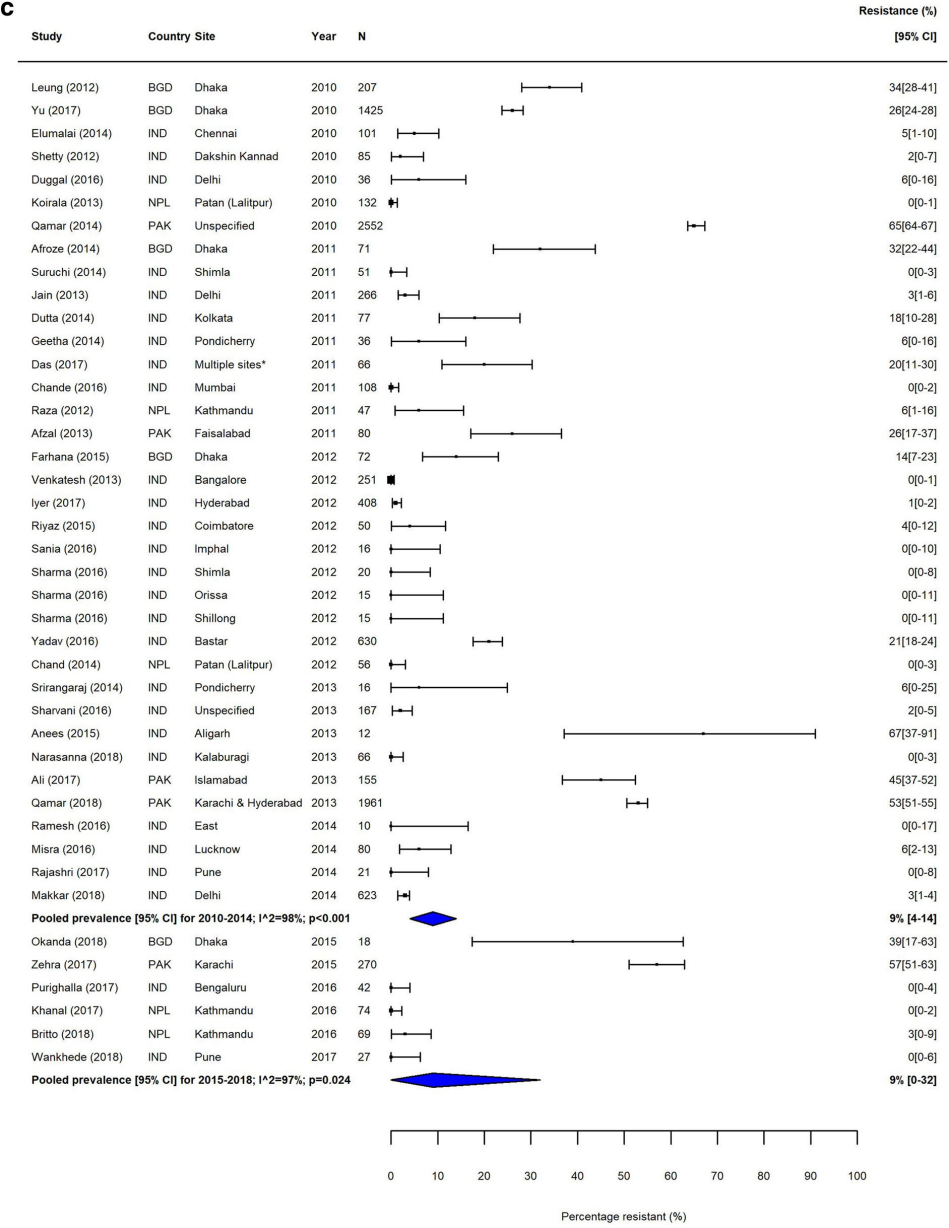
MDR *S.* Typhi in South Asia. Forest plots illustrating the prevalence of MDR amongst *S.* Typhi strains isolates in South Asia, grouped by 5‐year time periods. Individual study results are displayed with 95% confidence intervals; the pooled prevalence [95%CI] for each subgroup is represented by the blue diamond: **a** 1990–1999, **b** 2000–2009, and **c** 2010–2018. Multidrug resistance is defined as concurrent resistance against ampicillin, chloramphenicol and co‐trimoxazole

**Fig. 3:**
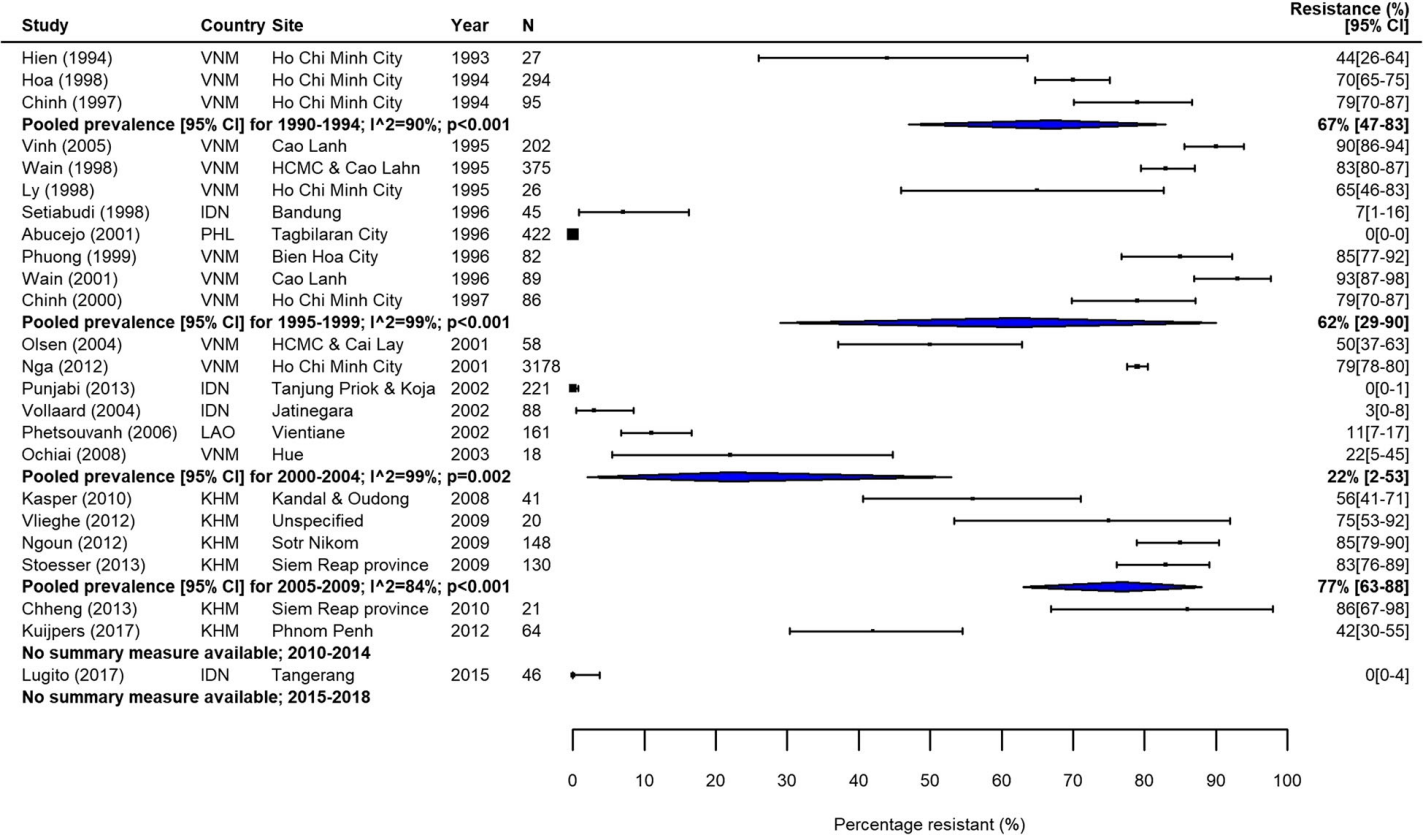
MDR *S.* Typhi in Southeast Asia. Forest plots illustrating the prevalence of MDR amongst *S.* Typhi strains isolates in Southeast Asia, grouped by 5‐year time periods. Individual study results are displayed with 95% confidence intervals; the pooled prevalence [95%CI] for each subgroup is represented by the blue diamond. Multidrug resistance is defined as concurrent resistance against ampicillin, chloramphenicol and co‐trimoxazole

**Fig. 4:**
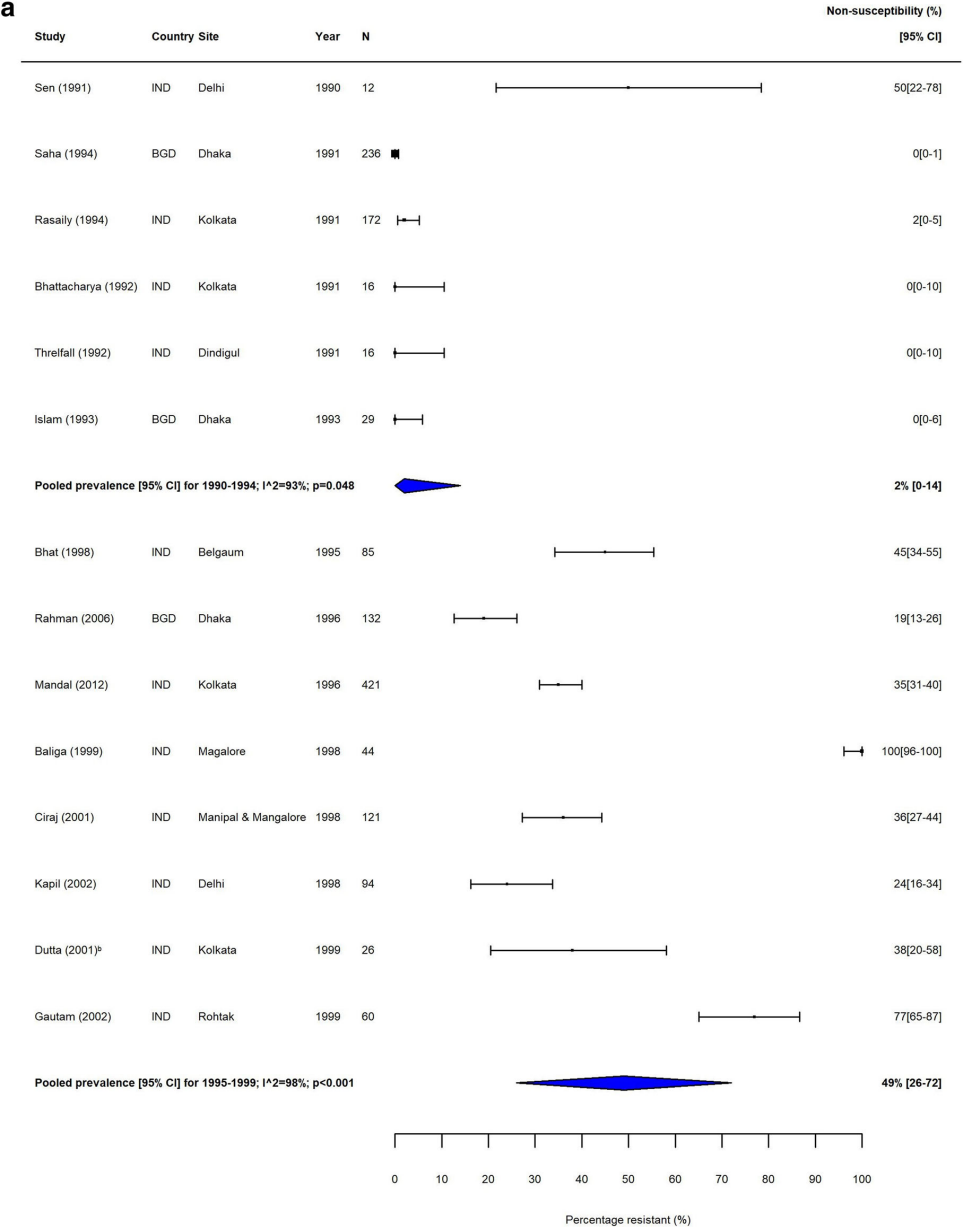

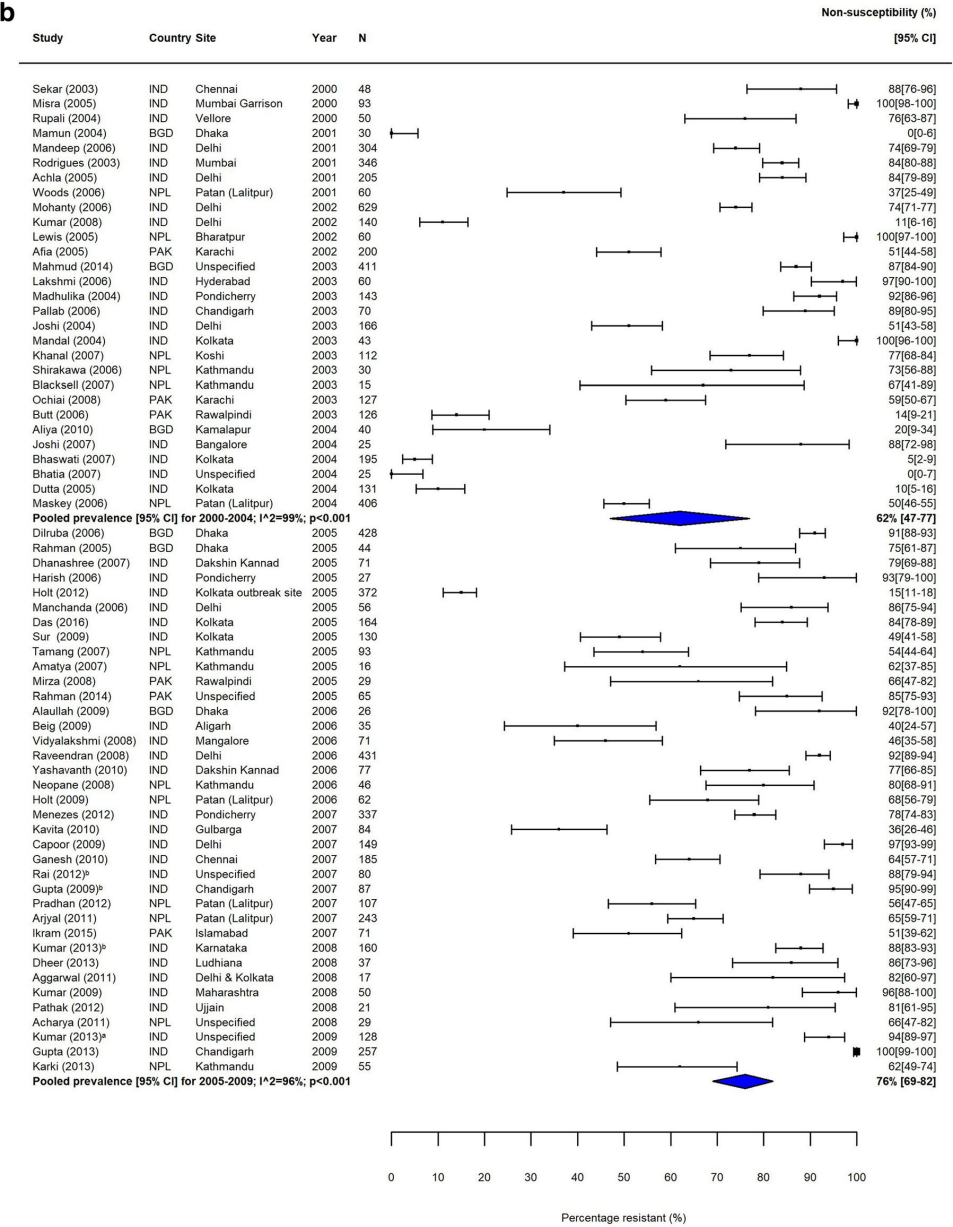

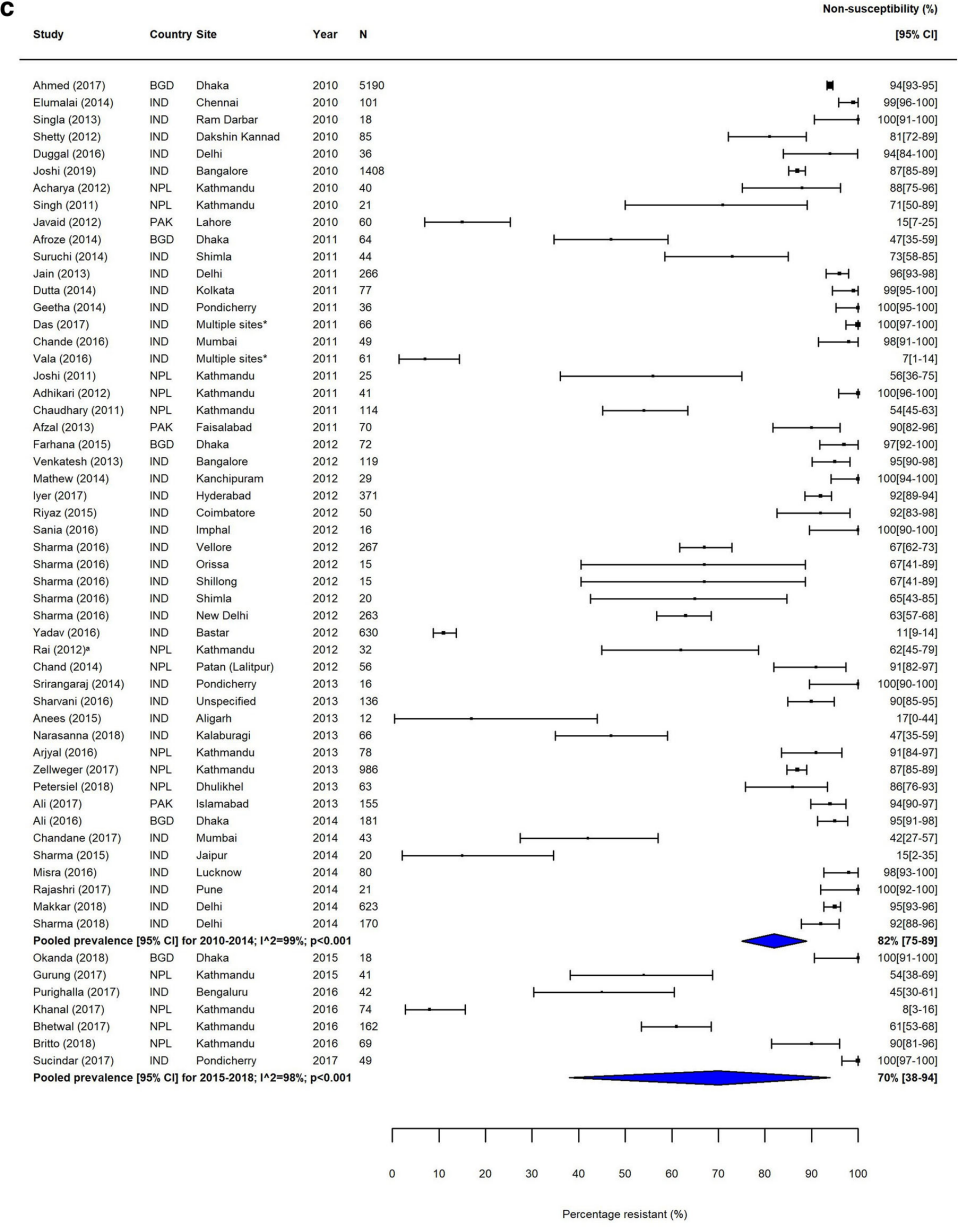
FQNS *S.* Typhi in South Asia. Forest plots illustrating the prevalence of FQNS amongst *S.* Typhi in South Asia, grouped by 5‐year time periods. Individual study results are displayed with 95% confidence intervals; the pooled prevalence [95%CI] for each subgroup is represented by the blue diamonds: **a** 1990–1999, **b** 2000–2009, and **c** 2010–2018. To allow the analysis of resistance trends over time despite typhoid‐specific breakpoint changes for ciprofloxacin (CLSI, 2012) coming into effect during our study (1990–2018), we categorised intermediate (ciprofloxacin MIC 0.12–0.5 μg/ml) and resistant strains isolates (≥ 1 μg/ml) according to the updated breakpoints, as well as isolates with ‘decreased ciprofloxacin (or fluoroquinolone) susceptibility’ (ciprofloxacin MIC 0.125–1.0 μg/ml) and nalidixic acid-resistant strains isolates (as proxy marker for ‘decreased ciprofloxacin (or fluoroquinolone) susceptibility’) as fluoroquinolone non‐susceptible (FQNS)

**Fig. 5:**
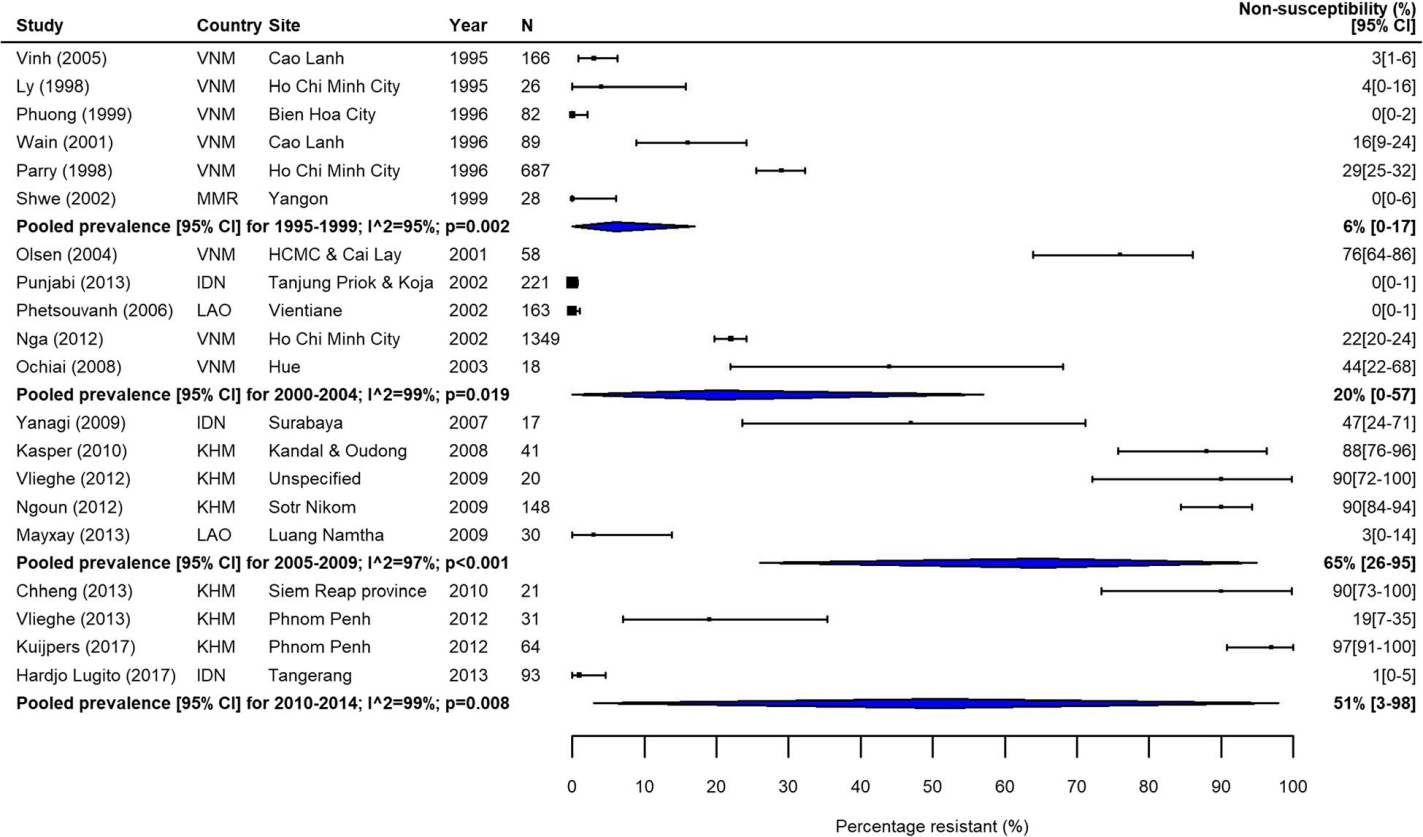
FQNS *S.* Typhi in Southeast Asia. Forest plots illustrating the prevalence of FQNS amongst *S.* Typhi in South Asia, grouped by 5‐year time periods. Individual study results are displayed with 95% confidence intervals; the pooled prevalence [95%CI] for each subgroup is represented by the blue diamonds

The proportion of MDR *S.* Typhi isolates showed a high degree of variation in the seven GBD regions over our study period. In South Asia, which includes the high-burden countries India, Nepal, Pakistan and Bangladesh, despite high heterogeneity, there was a clear downward trend in MDR over the study period (Table 3, Fig. 2). In contrast, the proportions of MDR *S.* Typhi isolates in Southeast Asia remained high, with pooled prevalence > 60% for most time periods (Table 3). The variability in resistance within this region was high; some countries, including the island nations of Indonesia and the Philippines, as well as Laos, reported low levels of MDR, whilst studies from neighbouring Vietnam and Cambodia reported considerably higher resistance levels (Fig. 3).

**Table 3:**
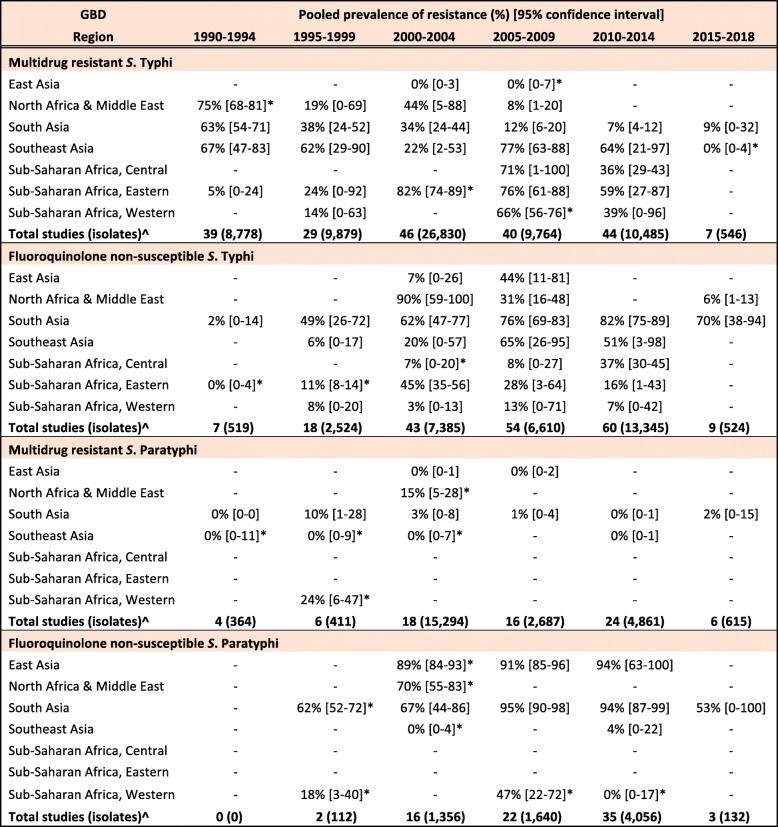
Pooled percentage prevalence [95% confidence intervals] of multidrug resistance and fluoroquinolone non-susceptibility amongst S. Typhi and S. Paratyphi A, grouped by region and five-year time-period

For sub-Saharan Africa, data were sparse, with only 21 (5%) studies examining the prevalence of MDR *S.* Typhi identified. For the Central sub-Saharan region, five studies were identified, all of which were conducted in DR Congo. Two studies were available for 2005–2009: one reported 30% MDR resistance (201 patients) [28] and a smaller study (11 patients), performed during a typhoid outbreak characterised by high rates of peritonitis and perforation, reported 100% MDR resistance [29]. For the period from 2010 to 2014, three studies were available which showed a pooled MDR prevalence of 36% (95% CI, 29–43%) for MDR *S.* Typhi (Table 3, Additional file 1: Figure S3a).

In Eastern sub-Saharan Africa, despite few data points, an increase in the proportion of MDR *S.* Typhi isolates was detectable during the study period (Additional file 1: Figure S3b). During 1990–1994, two studies from Kenya reported 0% (24 isolates) [30] and 13% (38 isolates) [31] MDR resistance, respectively, whilst in the following 5-year time periods this prevalence ranged from 60 to 82% in the Kenyan Isolates*.* In Malawi, no MDR was reported in 12 isolates during 1995–1999 [32], whilst in 2005–2009, 88% of 2054 isolates were MDR [33].

In Western sub-Saharan Africa, only six studies (seven data points) were available, with a large variability in results. No resistance was reported from Burkina Faso [34–36], whilst considerably higher levels of MDR were reported in Nigeria (37% (68 isolates)) in 1998 [37], increasing to 100% (58 isolates) in 2014 [38]) and Ghana with 63% (30 isolates) and 66% (89 isolates) [36, 39] (Additional file 1: Figure S3c).

A large variability of MDR in *S.* Typhi was also observed in North Africa and the Middle East (NAME), with studies from Egypt in 1998 [40], Saudi Arabia in 2003 [41] and Iraq in 2005 [42] showing particularly high levels of MDR with 67% (45 isolates), 100% (12 isolates) and 83% (59 isolates), respectively (Additional file 1: Figure S4). The pooled prevalence of MDR *S.* Typhi decreased from 44% (95% CI, 5–88%) during 2000–2004 to 8% (95%CI, 1–20%) during 2005–2009, due to a multi-centre study [42], which showed relatively low levels of MDR (Table 3). In East Asia, MDR *S*. Typhi was not reported in any of the four publications (Additional file 1: Figure S5).

Trends in FQNS amongst *S.* Typhi isolates differed from those of MDR across all regions, most likely reflecting changes in prescribing patterns and antimicrobial use that occurred during the study period. Despite high variability between and within countries, FQNS *S.* Typhi in South Asia increased steadily for each time period (Table 3, Fig. 4), from 2% (95%CI; 0–14%) in 1990–1994 to 81% (95%CI; 72%–89%) in 2010–2014 and 70% (95%CI; 38%–94%) in 2015–2018. Between 2010 and 2014, only seven of the 46 identified studies reported less than 50% of *S.* Typhi isolates as FQNS [43–49], highlighting the severity of this issue. Fewer studies were available from Southeast Asia. Similarly to South Asia, the proportions of FQNS *S.* Typhi increased steadily during our study period (Table 3, Fig. 5). Heterogeneity was extremely high in this region, and as with MDR, lower proportions of FQNS were reported in Indonesia.

In sub-Saharan Africa, few studies were available and results were highly variable (Additional file 1: Figure S6). The prevalence of FQNS *S.* Typhi was lower than in South and Southeast Asia, but comparatively high levels were reported in DR Congo in 2010 (41% of 17 isolates) [50] and in 2013 (37% of 164 isolates) [51], in Tanzania in 2010 (36% of 45 isolates) [52] and in Kenya (≥ 20%) in 2002 [53], 2005 [54] and 2013 [36]. In Western sub-Saharan Africa, no FQNS *S*. Typhi were reported in Burkina Faso [35, 36] and Ghana [39]; low levels (between 0 and 13%) in Senegal [55], south and central Nigeria (Lagos and Abuja) [37, 38, 56]; whilst comparatively higher proportions of FQNS (41% and 82%) were reported in northern Nigeria (Zaria and Kano) [57, 58].

In NAME, few reports were available. A multicentre study undertaken in Egypt, Iraq, Jordan and Qatar reported that the proportion of FQNS *S.* Typhi ranged between 17% in one location in Egypt and 81% in Iraq (Table 3, Additional file 1: Figure S7) [42]. Other studies reported no FQNS in Algeria [59] and only low levels of FQNS in Iraq in 2017 and 2018 [60, 61]. In East Asia, data were also limited but FQNS *S*. Typhi increased over time from 0% (15 isolates) reported by one study in 2002 [62] to 63% (139 isolates) in 2009 [63] (Additional file 1: Figure S8).

**Fig. 6:**
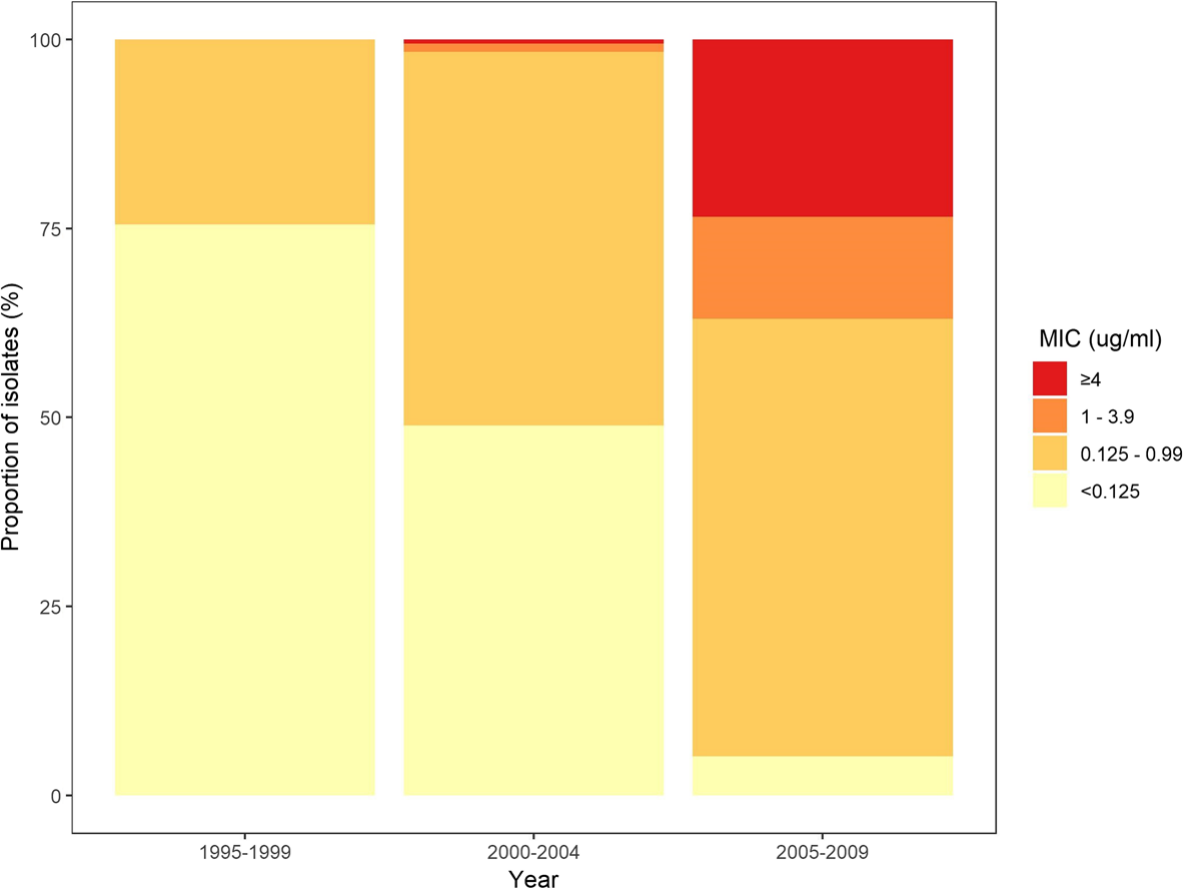
Stacked bar chart of *S*. Typhi ciprofloxacin MIC distributions. Data are from three selected studies [64–66] with sample sizes of more than 90 strains isolates performed in Delhi, India

In addition to our analysis of resistance frequencies, using data from three large studies in Delhi, India, we investigated changes in ciprofloxacin MIC distributions for *S.* Typhi over our study period [64–66]. Between 1995 and 2009, there was a large increase in the proportion of isolates with intermediate resistance (MIC 0.125–0.9 μg/mL), resistance (MIC 1–3.9 μg/mL), and high-level resistance (MIC ≥ 4 μg/mL), whilst the proportion of susceptible isolates decreased from 76% in 1995–1999 [64] to 23% in 2005–2009 [66] (Fig. 6). These data highlight increases in both the proportion and degree of resistance to ciprofloxacin in India during our study period.

The resistance pattern for *S.* Paratyphi A differed considerably to that of *S.* Typhi. In South Asia, the majority of studies reported either no or low levels of MDR; just six of 67 studies reported more than 20% MDR [46, 67–71]; five of these studies were from Pakistan. All five studies identified from Southeast Asia and the four from East Asia found 0% MDR in *S.* Paratyphi A (Additional file 1: Figures S9-S11). Contrary to this, FQNS was high amongst *S.* Paratyphi A in South Asia, with a pooled prevalence above 90% for 2000–2004, 2005–2009 and 2010–2014 (only one study available prior to 2000; Table 3, Additional file 1: Figure S12). Only three studies from Southeast Asia were identified; two from Indonesia [72, 73] found no FQNS and one from Cambodia [74] found 11% (183 isolates) FQNS *S.* Paratyphi A (Additional file 1: Figure S13). All six studies from East Asia were from 2004 onwards and found very high levels of FQNS in *S.* Paratyphi A (Additional file 1: Figure S14).

There were few reports of *S.* Paratyphi A in Africa and the Middle East where the burden of disease is not well described [23]. Two studies reported MDR *S.* Paratyphi A, with 24% in Nigeria [37] and 15% in Kuwait [75]. Three studies reported FQNS *S.* Paratyphi A, with 18% [37] and 47% [57] in Nigeria and 70% in Kuwait [75]. Kuwait has a large migrant worker population from South Asia, and although we tried to exclude imported cases, this may have affected the level of resistance observed here.

*S.* Typhi ceftriaxone susceptibility data were extracted from 198 studies (221 data points; Additional file 2: Data file S3). Of these, 59 (27%) studies reported at least one resistant isolate; the majority of these studies originated from South Asia. For the 34 studies that reported at least one organism, but ≤ 5% ceftriaxone-resistant isolates, disc diffusion was the standard testing method. Only five studies additionally determined MICs: two by agar dilution [45, 65] and three by *E*-test [76–78]. However, ceftriaxone resistance was not confirmed by *E*-test in one study [76]. Only two studies tested for extended-spectrum beta-lactamase (ESBL) production [65, 76]; both reported negative results.

Of the 31 studies reporting more than 5% ceftriaxone resistance (range 6–45%), six failed to report susceptibility testing methods [79–84]. Three studies used automated testing methods; two of those used VITEK2 testing and reported 25% (220 isolates) [85] and 33% (30 isolates) resistance [61], and one study deployed the BD Phoenix 100 system and reported 12% (42 isolates) resistance. One study reported 6% (16 isolates) ceftriaxone resistance using microdilution testing [86]. The remaining 21 studies performed Kirby-Bauer disc diffusion testing; of those, only three studies, one with 43% (80 isolates) [87], one with 15% (630 isolates) [46] and one with 11% (300 isolates) resistance [88], determined ceftriaxone MICs. However, ceftriaxone resistance was not confirmed by MIC testing (agar dilution) in the two latter studies [46, 88]. Only two of the 21 studies [87, 89] tested for ESBL-production, with Afzal et al. reporting negative results [87].

Twenty-three studies, mostly from South Asia, reported at least one ceftriaxone-resistant *S*. Paratyphi A isolate (Additional file 2: Data file S3). Sixteen studies reported at least one organism, but ≤ 5% ceftriaxone resistant isolates; of these, four used automated systems (VITEK 2, RapidATB) or microdilution. The remaining studies performed disc diffusion, but none of these determined MICs. Only one study with 3% resistance described ESBL-testing, but did not report the results [90]. Seven studies reported > 5% ceftriaxone resistance. Of these, one study reported 17% (157 strains) resistance using VITEK2 [91], and one study performed agar dilution for all the isolates and reported intermediate resistance (MIC < 2 μg/mL) for 100% (27/27) of the isolates, but ESBL-testing was not performed [92]. The remaining five studies (resistance between 6 and 13%) used Kirby-Bauer disc diffusion testing, and in addition, three of the studies performed MIC testing by agar dilution [46, 88, 93]. However, ceftriaxone resistance was confirmed by MIC testing in only one study [93].

Due to the lack of CLSI interpretive criteria for azithromycin and *S.* Typhi before 2015, fewer studies (59/384; 15%) reported azithromycin susceptibility testing. Data from 22 studies could be standardised according to the BSAC guidelines [94]. Resistant isolates were those with an MIC > 16 μg/ml [epidemiological cut-off value; corresponding to EUCAST 2014 guidelines] or by disc diffusion, a zone diameter ≤ 18 mm (Additional file 1: Table S5). Fourteen studies reported no resistance against azithromycin and five identified < 10% resistance. Azithromycin resistance of 13% (16 isolates), 34% (80 isolates) and 85% (71 isolates) in *S.* Typhi was reported by studies conducted in India and Pakistan [95–97]. The first two studies performed Kirby-Bauer disc diffusion testing and MIC determination by *E*-test, whilst the latter study deployed disc diffusion testing only.

## Discussion

Our systematic review and meta-analysis, encompassing 384 articles equating to 94,616 *S*. Typhi and 29,731 *S*. Paratyphi A isolates (124,347 isolates in total), provides comprehensive evidence of the magnitude and geographic extent of the AMR problem in enteric fever. We described and analysed the consecutive emergence of resistance to different antimicrobial classes, reflecting the potential selection pressure imposed by the use of differing antimicrobials to treat enteric fever [98].

The start of our study coincided with the height of the MDR *S*. Typhi epidemic in South and Southeast Asia in the early 1990s [14, 99]. The subsequent change to the FQs, which have excellent pharmacological properties and are recommended as the treatment of choice [11], led to the emergence of FQNS isolates, characterised by mutations in the FQ target genes which determine nalidixic acid resistance and higher FQ MICs. These organisms were associated with poor clinical outcomes and higher rates of complications [18, 20]. The rise in FQNS necessitated a switch to parenteral ceftriaxone, which requires hospitalisation, cefixime, which has been demonstrated to be clinically less effective [100], or azithromycin. However, resistance to these antimicrobials is also on the increase, exemplified by the worrying recent emergence of extensively drug-resistant (XDR) *S*. Typhi organisms in Pakistan [101], exhibiting MDR, FQR and ceftriaxone-resistance. In Africa, MDR *S.* Typhi is widespread and FQNS is posing a new treatment problem, with alternative antimicrobials like azithromycin and ceftriaxone either not routinely available or unaffordable in resource-limited settings. Our study emphasises the contribution of *S*. Paratyphi A to the AMR problem. Although the prevalence of MDR *S.* Paratyphi A was low, we found high prevalence of FQNS *S*. Paratyphi A in China, India, Nepal and Bangladesh, often with FQ MICs exceeding those of *S.* Typhi [102]. Apart from a trend of decreasing MDR *S*. Typhi in South Asia (with the exception of Pakistan) and NAME, resistance to the first-line antimicrobials and the FQs has increased in enteric fever and is widely distributed, with ceftriaxone and azithromycin resistance [103, 104] becoming evident.

We aimed to perform an exhaustive search and provide a comprehensive synthesis of all relevant articles examining AMR enteric fever. This yielded a large number of studies, with differences in patient selection and blood culture sampling criteria, as well as such with incomplete description of microbiological methods. However, the AMR trends shown in our analysis are corroborated by longitudinal studies performed by single centres according to standard clinical protocols, utilising microbiology laboratories with international EQA schemes or ISO accreditation (C. Dolecek, personal observations) [18, 33, 53, 77, 102, 105–108].

Apart from highlighting temporal trends in the seven GBD regions, our study also points at the variability of resistance between countries and even within a single country (e.g. India, Vietnam). The variability between neighbouring countries is particularly notable in Southeast Asia, with low levels of MDR and FQNS resistance in Laos and Indonesia. This is likely due to a mixture of factors, which include the scarcity of microbiological facilities which might not allow a complete picture, differing patterns of economic development, medical infrastructure and antimicrobial consumption, differences in transmission and circulating organism genotypes [42, 109]. We plan to explore these potential sources of heterogeneity in-depth, using individual patient datasets and data on antimicrobial consumption, and also to investigate fine-scale geographic variation in resistance levels using a geostatistical modelling framework [21]. Despite estimates of typhoid fever incidence at almost 500 infections per 100,000 persons per year in India [62] and high incidences in both rural and urban locations in Africa [36], the majority of studies had relatively small sample sizes, reflecting treatment seeking behaviour, antimicrobial pretreatment and the low sensitivity of blood culture [11, 12, 110]. In LMICs, patients may not have access to or are prevented from attending health facilities due to high out-of-pocket costs, therefore seeking treatment over the counter from community pharmacies or in the informal sector. Even if blood culture is available, it is often not performed, as each test incurs further costs for the patient [111]. These factors contribute to the underrepresentation of culture confirmed enteric fever and bacterial infections in more general.

Our systematic review highlights many data gaps. For example, the majority of studies were performed on the Indian Subcontinent, where the burden of enteric fever is the highest [1, 62]. In Africa, despite a considerable disease burden [36], AST data were only available in ten countries, where they tended to be reported by a small number of institutions. Data were particularly sparse in West and Central Africa. Yet, routine surveillance of AMR is critical to assess the effectiveness of antimicrobial regimens and to guide local and national treatment policies [17]. The paucity of microbiology facilities and the consequent lack of AST data to inform empirical antimicrobial treatment have ramifications beyond enteric fever [3, 112, 113]. Although there is no defined AMR threshold for enteric fever, there is agreement that 20% AMR prevalence should trigger a change in empirical treatment policy for uncomplicated bacterial infections, with a lower threshold for life-threatening infections and outpatients [114]. Our forest plots do not include a vertical bar to indicate this clinical threshold, but once resistance is 20% and above, this specific antimicrobial regimen is considered ineffective and unsafe and should no longer be used for empirical treatment in this setting. Conversely, the lack of data from South America and South Africa is consistent with a low incidence of enteric fever as consequence of economic progress and improvements in water safety over the last decades [115, 116], therefore not currently fitting the definition of an endemic region [117].

Another key finding of our systematic review is the difference in the quality and reporting of public health AST data. Conforming to previous systematic reviews [3, 113, 118–120], we identified incomplete AST reporting in many studies. The absence of robust AST data may reflect weaknesses, varying testing methodology and performance across laboratories in LMICs, but partially also reflects the lack of clear and coherent reporting guidelines for microbiology data, comparable to STROBE [121]. There is ambiguity whether the statement, ‘AST was performed according to CLSI 2008’, encompasses all recommended steps, including control organisms and confirmatory testing of possible ESBL-producing organisms. Less than a third of studies in our dataset described internal quality controls; this concurs with data from Tadesse (48%; 69/144) [119] and Leopold (47%; 120/256) [113]. In addition, several laboratories failed to state their participation in EQA schemes. This included well-resourced and established laboratories where EQA participation is known to take place but the authors did not specify this. Recently, reporting guidelines for microbiology data (Microbiology Investigation Criteria for Reporting Objectively; MICRO) were published [122]; compliance with MICRO guidelines will be greatly beneficial for future AMR surveillance and research.

We identified several studies that reported higher proportions of ceftriaxone- and azithromycin-resistant *S*. Typhi organisms than a recently published smaller systematic review on resistance determinants of *S.* Typhi [123]; this discrepancy may reflect methodological weaknesses. The majority of studies with a high proportion of ceftriaxone-resistant isolates performed disc susceptibility testing only, without further MIC and ESBL-testing. Whilst these comply with current CLSI guidelines (the recommendations to perform confirmatory tests were removed in 2010), we identified several studies that reported conflicting results between disc susceptibility testing and MIC testing (i.e. resistance not confirmed by MIC) [46, 76, 88], raising doubt about the reliability of some of these results. For example, one study reported 100% intermediate ceftriaxone resistance but did not investigate ESBL-production or discuss these alarming results [92].

High-quality AST testing is an important public health tool; identifying AMR will guide practitioners towards effective antimicrobial regimens and enable updated local treatment guidelines, whilst at the same time it also prevents practitioners being steered away from effective antimicrobials due to unsubstantiated results. These issues are very important as our antimicrobial armamentarium is limited.

## Limitations

Our study has several limitations. First, there was high statistical heterogeneity (*I*^2^ > 80%) within each subgroup, raising issues about performing random meta-analysis of MDR and FQNS *S*. Typhi and *S.* Paratyphi A. We conducted a sensitivity analysis aimed at explaining some of this heterogeneity. We removed studies with characteristics that were most likely to contribute to the heterogeneity, i.e. studies with less robust methodological detail and small sample sizes. However, we still identified high heterogeneity and the pooled prevalence estimates remained very similar. We conducted these sensitivity analyses on data from South Asia; there were insufficient studies for other geographical regions. Furthermore, we performed a comparison of the pooled prevalence of the meta-analysis with the median prevalence of resistance, as a more conservative summary estimate [112, 113], and found an excellent correlation for all subgroups. One caveat of our study is that the pooled prevalence for some regions and 5-year time periods had wide confidence intervals; this was especially true in Africa, due to few studies with relatively small sample sizes and heterogeneity in the size of the effect. Second, it is likely that policies of taking blood cultures, which include patient selection and blood volumes, will differ across hospitals in LMICs, and often, these policies may not be consistently implemented. Therefore, the included studies might have suffered from selection and sampling bias. Only a few studies were conducted as multicentre studies and used the same protocol at all sites [36, 42, 124] or provided data over long periods of time using the same patient selection criteria for blood culture (e.g. [33, 77, 102, 107]); these studies support our findings. The majority of studies were performed by routine hospital microbiology departments and did not report clinical information, numbers of annual blood cultures screened (as recommended by WHO GLASS [17]) or the denominator population. It might be that only very sick patients had blood cultures performed, and therefore, the proportion of resistance could possibly be overestimated [125]. Additionally, most studies were performed in urban centres, which have a higher availability of antimicrobial drugs, and AMR patterns in rural areas may be different. However, due to the emergence and expansion of certain very successful lineages, especially the MDR and FQNS H58 haplotype [126], we would predict these organisms to be well distributed.

Notably, despite searching seven databases, we will have missed papers, including non-English language papers that were not indexed in any of these databases.

Fourth, the analysis of resistance could have been impeded by interpretive breakpoint changes for two classes of antimicrobials. Third-generation cephalosporin breakpoints for Enterobacteriaceae were lowered in 2010 [127]. Ceftriaxone-resistant enteric fever was rare at this time; therefore, we did not expect significant changes to the resistant proportions. Of more significance, however, were the lower FQ breakpoints for enteric fever that came into effect in 2012 [24]. We recategorised isolates to allow the analysis of FQNS *S*. Typhi and *S*. Paratyphi A over the study period. However, this approach is likely to be an underestimation, as nalidixic acid testing does not capture all mechanisms of decreased FQ susceptibility [18]. Furthermore, none of the studies used pefloxacin (instead of ciprofloxacin) discs for the detection of low-level ciprofloxacin resistance [128]. Again, this might have led to a slight underestimation of the FQNS proportions. Additionally, the relatively wide range of MICs, the FQNS isolates will cover, has to be noted. The forest plots present categorical data (resistant/intermediate), with ciprofloxacin MICs ranging from above 0.06 to beyond 32 μg/mL. For isolates with lower MICs, the fluoroquinolones, especially if given at higher doses, might still achieve cure, albeit delayed [18]. However, as documented, not only the proportions but also the degree of resistance (expressed by the MICs) have increased during the study period and high-level FQ resistance is now prevalent in South Asia; therefore, FQ treatment is inappropriate.

## Conclusions

Despite these limitations, our report gives conclusive evidence that AMR amongst *S.* Typhi and *S.* Paratyphi A is worsening. Antimicrobial treatment of a primarily preventable infection fuels antimicrobial use [129] and contributes to the resistance problem by exposing bystander organisms to antimicrobials [130]. Interventions that reduce the number of enteric fever infections are urgently needed [131]; improvements of sanitation and water quality must be prioritised to reduce the burden of this and other water-borne infections. In the meantime, the GAVI endorsement and deployment of conjugate typhoid vaccines offer hope that the burden of typhoid fever will reduce in the near future. However, there remains no licenced vaccine for *S.* Paratyphi A. In addition, better surveillance of AMR, including standardised reporting of AST data and rollout of external quality control assessment, is urgently needed to facilitate evidence-based treatment policy and practice.

## Supplementary information

**Additional file 1.** Supplementary Materials.

**Additional file 2.** **Data file S1:** Individual study results for multidrug resistance. **Data file S2:** Individual study results for fluoroquinolone non-susceptibility. **Data file S3:** Indivual study results for ceftriaxone non-susceptibility.

## Data Availability

All data generated or analysed during this study are included in this published article and its supplementary information files.

## Abbreviations

AMR: Antimicrobial drug resistance, antimicrobial drug resistant
AST: Antimicrobial susceptibility testing
CLSI: Clinical and Laboratory Standards Institute
CI: Confidence interval
ESBL: Extended spectrum beta-lactamases
EQA: External quality assurance
FQ: Fluoroquinolone
GAVI: The Vaccine Alliance (formerly the Global Alliance for Vaccines and Immunisation)
GBD: Global Burden of Disease Study
HIV: Human Immunodeficiency Virus
GLASS: Global Antimicrobial Resistance Surveillance System
LMIC: Low- and middle-income country
MIC: Minimum inhibitory concentration
MICRO: Microbiology Investigation Criteria for Reporting Objectively
MDR: Multidrug resistance
NAME: North Africa and the Middle East
PRISMA: Preferred Reporting Items for Systematic Reviews and Meta-Analysis
*S*. Typhi: *Salmonella enterica* serovar Typhi
*S*. Paratyphi: *Salmonella enterica* serovar Paratyphi
STROBE: Strengthening the Reporting of Observational Studies in Epidemiology
WHO: World Health Organization

## References

[1] GBD Typhoid and Paratyphoid Collaborators (2019). The global burden of typhoid and paratyphoid fevers: a systematic analysis for the Global Burden of Disease Study 2017. Lancet Infect Dis.

[2] Deen J, von Seidlein L, Andersen F, Elle N, White NJ, Lubell Y (2012). Community-acquired bacterial bloodstream infections in developing countries in South and Southeast Asia: a systematic review. Lancet Infect Dis.

[3] Reddy EA, Shaw AV, Crump JA (2010). Community-acquired bloodstream infections in Africa: a systematic review and meta-analysis. Lancet Infect Dis.

[4] Maskey AP, Day JN, Phung QT, Thwaites GE, Campbell JI, Zimmerman M (2006). Salmonella enterica serovar Paratyphi A and S. enterica serovar Typhi cause indistinguishable clinical syndromes in Kathmandu, Nepal. Clin Infect Dis.

[5] Ochiai RL, Wang X, von Seidlein L, Yang J, Bhutta ZA, Bhattacharya SK (2005). Salmonella paratyphi A rates. Asia Emerg Infect Dis.

[6] Crump JA (2012). Typhoid fever and the challenge of nonmalaria febrile illness in sub-saharan Africa. Clin Infect Dis.

[7] D'Acremont V, Kilowoko M, Kyungu E, Philipina S, Sangu W, Kahama-Maro J (2014). Beyond malaria--causes of fever in outpatient Tanzanian children. N Engl J Med.

[8] Guerin P, Hopkins H, Thomas N, Elven J, Das D, Eyers J, et al. Mapping the aetiology of non-malarial febrile illness globally in malaria-endemic regions: a systematic review. PROSPERO 2016 CRD42016049281. 2016; https://www.crd.york.ac.uk/prospero/display_record.php?ID=CRD42016049 281.

[9] Abhilash KP, Jeevan JA, Mitra S, Paul N, Murugan TP, Rangaraj A (2016). Acute undifferentiated febrile illness in patients presenting to a tertiary care hospital in South India: clinical spectrum and outcome. J Glob Infect Dis.

[10] Murdoch DR, Woods CW, Zimmerman MD, Dull PM, Belbase RH, Keenan AJ (2004). The etiology of febrile illness in adults presenting to Patan Hospital in Kathmandu, Nepal. Am J Trop Med Hyg.

[11] World Health Organisation. Vaccines and Biologicals. Background document: The diagnosis, treatment and prevention of typhoid fever. Geneva: 2003. https://www.glowm.com/pdf/WHO-diagnosis%20treatment%20prevention%20of%20typhoid%20fever-2003-CustomLicense.pdf. Assessed 18 Apr 2019.

[12] Arjyal A, Basnyat B, Koirala S, Karkey A, Dongol S, Agrawaal KK (2011). Gatifloxacin versus chloramphenicol for uncomplicated enteric fever: an open-label, randomised, controlled trial. Lancet Infect Dis.

[13] Wain J, Diep TS, Ho VA, Walsh AM, Nguyen TT, Parry CM (1998). Quantitation of bacteria in blood of typhoid fever patients and relationship between counts and clinical features, transmissibility, and antibiotic resistance. J Clin Microbiol.

[14] Parry CM, Hein TT, Dougan G, White NJ, Farrar JJ (2002). Typhoid fever. N Engl J Med.

[15] World Health Organisation. Global strategy for containment of antimicrobial resistance. https://www.who.int/drugresistance/WHO_Global_Strategy_English.pdf?ua=1. Accessed 22 Nov 2018. 2001.

[16] World Health Organisation. Antimicrobial resistance: global report on surveillance 2014. http://apps.who.int/iris/bitstream/handle/10665/112642/9789241564748_eng.pdf?sequence=1. Accessed 22 Nov 2018.

[17] World Health Organisation. Global antimicrobial resistance surveillance system; manual for early implementation. 2015. https://apps.who.int/iris/bitstream/handle/10665/188783/9789241549400_eng.pdf?sequence=1. Accessed 18 Apr 2019.

[18] Parry CM, Vinh H, Chinh NT, Wain J, Campbell JI, Hien TT (2011). The influence of reduced susceptibility to fluoroquinolones in Salmonella enterica serovar Typhi on the clinical response to ofloxacin therapy. PLoS Negl Trop Dis.

[19] Parry C. M., Ho V. A., Phuong L. T., Bay P. V. B., Lanh M. N., Tung L. T., Tham N. T. H., Wain J., Hien T. T., Farrar J. J. (2006). Randomized Controlled Comparison of Ofloxacin, Azithromycin, and an Ofloxacin-Azithromycin Combination for Treatment of Multidrug-Resistant and Nalidixic Acid-Resistant Typhoid Fever. Antimicrobial Agents and Chemotherapy.

[20] Mandeep W, Rajni G, Rajesh M, Premila P, Pushpa A, Mani K (2005). Current perspectives of enteric fever: a hospital-based study from India. Ann Trop Paediatr.

[21] Hay SI, Rao PC, Dolecek C, Day NPJ, Stergachis A, Lopez AD (2018). Measuring and mapping the global burden of antimicrobial resistance. BMC Med.

[22] Moher D, Liberati A, Tetzlaff J, Altman DG, The PG (2009). Preferred Reporting Items for Systematic Reviews and Meta-Analyses: The PRISMA statement. PLoS Med.

[23] Arndt MB, Mosites EM, Tian M, Forouzanfar MH, Mokhdad AH, Meller M (2014). Estimating the burden of Paratyphoid A in Asia and Africa. PLoS Negl Trop Dis.

[24] Clinical and Laboratory Standards Institute (2012). Performance standards for antimicrobial susceptibility testing; twenty-second informational supplement. CLSI document M100-S22.

[25] Agresti A, Coull BA (1998). Approximate is better than “Exact” for interval estimation of binomial proportions. Am Stat.

[26] Higgins JP, Thompson SG, Deeks JJ, Altman DG (2003). Measuring inconsistency in meta-analyses. Bmj..

[27] Barendregt JJ, Doi SA, Lee YY, Norman RE, Vos T (2013). Meta-analysis of prevalence. J Epidemiol Community Health.

[28] Lunguya O, Lejon V, Phoba MF, Bertrand S, Vanhoof R, Verhaegen J (2012). Salmonella Typhi in the Democratic Republic of the Congo: Fluoroquinolone decreased susceptibility on the rise. PLoS Negl Trop Dis.

[29] Muyembe-Tamfum JJ, Veyi J, Kaswa M, Lunguya O, Verhaegen J, Boelaert M (2009). An outbreak of peritonitis caused by multidrug-resistant Salmonella Typhi in Kinshasa, Democratic Republic of Congo. Travel Med Infect Dis.

[30] Dougle M, Hendriks E, Sanders E, Dorigo-Zetsma JW (1997). Laboratory investigations in the diagnosis of septicaemia and malaria. East Afr Med J.

[31] Kariuki S, Gilks C, Revathi G, Hart CA (2000). Genotypic analysis of multidrug-resistant Salmonella enterica Serovar Typhi, Kenya. Emerg Infect Dis.

[32] Gordon MA, Walsh AL, Chaponda M, Soko D, Mbvwinji M, Molyneux ME (2001). Bacteraemia and mortality among adult medical admissions in Malawi - predominance of non-Typhi Salmonellae and Streptococcus pneumoniae. J Infect.

[33] Feasey Nicholas A., Gaskell Katherine, Wong Vanessa, Msefula Chisomo, Selemani George, Kumwenda Save, Allain Theresa J., Mallewa Jane, Kennedy Neil, Bennett Aisleen, Nyirongo Joram O., Nyondo Patience A., Zulu Madalitso D., Parkhill Julian, Dougan Gordon, Gordon Melita A., Heyderman Robert S. (2015). Rapid Emergence of Multidrug Resistant, H58-Lineage Salmonella Typhi in Blantyre, Malawi. PLOS Neglected Tropical Diseases.

[34] Ki-Zerbo GA, Sawadogo AB, Kyelem N, Zoubga A, Thiombiano R, Durand G (2000). Enterobacteriaceae bacteriemia in human deficiency virus seropositive in patients at Bobo-Dioulasso hospital (Burkina Faso): study of 26 cases. Medecine et Maladies Infectieuses..

[35] Maltha Jessica, Guiraud Issa, Kaboré Bérenger, Lompo Palpouguini, Ley Benedikt, Bottieau Emmanuel, Van Geet Chris, Tinto Halidou, Jacobs Jan (2014). Frequency of Severe Malaria and Invasive Bacterial Infections among Children Admitted to a Rural Hospital in Burkina Faso. PLoS ONE.

[36] Marks F, Kalckreuth V, Aaby P, Adu-Sarkodie Y, Tayeb M, Ali M (2017). Incidence of invasive Salmonella disease in sub-Saharan Africa: a multicentre population-based surveillance study. Lancet Glob Health.

[37] Akinyemi KO, Coker AO, Olukoya DK, Oyefolu AO, Amorighoye EP, Omonigbehin EO (2000). Prevalence of multi-drug resistant Salmonella Typhi among clinically diagnosed typhoid fever patients in Lagos, Nigeria. Zeitschrift fur Naturforschung Section C Journal of Biosciences.

[38] Akinyemi KO, Oyefolu AOB, Mutiu WB, Iwalokun BA, Ayeni ES, Ajose SO (2018). Typhoid fever: tracking the trend in Nigeria. (Special Issue: tackling typhoid - what do global and country trends teach us?). Am J Trop Med Hyg.

[39] Eibacha D, Al-Emrana HM, Dekker DM, Krumkamp R, Yaw A-S, Espinoza LMC (2016). The emergence of reduced ciprofloxacin susceptibility in Salmonella enterica causing bloodstream infections in rural Ghana. Clin Infect Dis.

[40] Abdel Wahab MF, el-Gindy IM, Sultan Y, el-Naby HM. Comparative study on different recent diagnostic and therapeutic regimens in acute typhoid fever. J Egypt Public Health Assoc 1999;74(1–2):193–205. 17216959

[41] Panhotra BR, Saxena AK, Al-Arabi Al-Ghamdi AM (2004). Emerging nalidixic acid and ciprofloxacin resistance in non-typhoidal Salmonella isolated from patients having acute diarrhoeal disease. Ann Saudi Med.

[42] Rahman BA, Wasfy MO, Maksoud MA, Hanna N, Dueger E, House B (2014). Multi-drug resistance and reduced susceptibility to ciprofloxacin among Salmonella enterica serovar Typhi isolates from the Middle East and Central Asia. New Microbes New Infect.

[43] Javaid H, Zafar A, Ahmed JM, Ejaz H, Zubair M (2012). Changing patterns of antimicrobial susceptibility of salmonella Typhi at the children’s hospital Lahore. Pakistan J Med Health Sci.

[44] Afroze SR, Rahim MA, Hasan MM, Afroz F, Haque HF, Ahmed JU (2014). Pattern of antibiotic sensitivity in enteric fever: a tertiary care hospital experience. J Med.

[45] Vala S, Shah U, Ahmad SA, Scolnik D, Glatstein M (2016). Resistance patterns of typhoid fever in children: a longitudinal community-based study. Am J Ther.

[46] Yadav VC, Kiran VR, Sharma R (2016). Enteric fever in Bastar tribal region-prevalence and sensitivity patterns. J Evol Med Dental Sciences-Jemds.

[47] Anees A, Indu S, Fatima K, Anjum P (2015). Multi-drug resistant *Salmonella enterica* subspecies enterica serotype Typhi: a diagnostic and therapeutic challenge. Int J Curr Microbiol Appl Sci.

[48] Chandane P, Gandhi A, Bowalekar S (2017). Study of antibiotic susceptibility pattern of Salmonella Typhi in children suffering from enteric fever. Ann Trop Med Public Health.

[49] Sharma, Sharma R, Gupta S (2015). Bacteriological analysis of blood culture isolates with their antibiogram from a tertiary care hospital. Int J Pharm Sci Res.

[50] Phoba MF, Lunguya O, Mayimon DV, di Mputu PL, Bertrand S, Vanhoof R (2012). Multidrug-resistant Salmonella enterica, Democratic Republic of the Congo. Emerg Infect Dis.

[51] Kalonji Lisette Mbuyi, Post Annelies, Phoba Marie-France, Falay Dadi, Ngbonda Dauly, Muyembe Jean-Jacques, Bertrand Sophie, Ceyssens Pieter-Jan, Mattheus Wesley, Verhaegen Jan, Barbé Barbara, Kuijpers Laura, Van Geet Chris, Lunguya Octavie, Jacobs Jan (2015). InvasiveSalmonellaInfections at Multiple Surveillance Sites in the Democratic Republic of the Congo, 2011–2014. Clinical Infectious Diseases.

[52] Thriemer Kamala, Ley Benedikt, Ame Shaali, von Seidlein Lorenz, Pak Gi Deok, Chang Na Yoon, Hashim Ramadhan, Schmied Wolfgang Hellmut, Busch Clara Jana-Lui, Nixon Shanette, Morrissey Anne, Puri Mahesh K., Ali Mohammad, Ochiai R. Leon, Wierzba Thomas, Jiddawi Mohammad S., Clemens John D., Ali Said M., Deen Jaqueline L. (2012). The Burden of Invasive Bacterial Infections in Pemba, Zanzibar. PLoS ONE.

[53] Kariuki S, Revathi G, Muyodi J, Mwituria J, Munyalo A, Mirza S (2004). Characterization of multidrug-resistant typhoid outbreaks in Kenya. J Clin Microbiol.

[54] Mengo DM, Kariuki S, Muigai A, Revathi G (2010). Trends in Salmonella enteric serovar Typhi in Nairobi, Kenya from 2004 to 2006. Journal of Infection in Developing Countries..

[55] Lefebvre N, Gning SB, Nabeth P, Ka S, Ba-Fall K, Rique M (2005). Clinical and laboratory features of typhoid fever in Senegal. A 70-case study [French]. Med Trop.

[56] Akinyemi KO, Smith SI, Bola Oyefolu AO, Coker AO (2005). Multidrug resistance in Salmonella enterica serovar typhi isolated from patients with typhoid fever complications in Lagos, Nigeria. Public Health.

[57] Adeshina GO, Osuagwu NO, Okeke CLE, Ehinmidu JO, Bolaji RO (2009). Prevalence and susceptibility of Salmonella Typhi and Salmonella Paratyphi in Zaria, Nigeria. Int J Health Res.

[58] Abdullahi M, Olonitola SO, Umoh VJ, Inabo IH (2015). Antibacterial resistance profile and PCR detection of antibiotic resistance genes in Salmonella serovars isolated from blood samples of hospitalized subjects in Kano, North-West, Nigeria. Brit Microbiol Res J.

[59] Bouzenoune F, Debbih KK, Boudersa F, Kouhil S, Nezzar N (2011). Antibiotic susceptibility of Salmonella enterica serovar Typhi isolated from blood cultures at the Ain M'lila hospital (Algeria), between 2005 and 2008. [French]. Med et Maladies Infectieuses.

[60] Aljanaby AAJ, Medhat AR (2017). Prevalence of some antimicrobials resistance associated-genes in Salmonella Typhi isolated from patients infected with typhoid fever. J Biol Sci.

[61] Al-Abbasy AJ (2018). Molecular study of antibiotic resistance gene in salmonella enterica serovar typhi isolates. Int J Pharm Res.

[62] Ochiai RL, Acosta CJ, Danovaro-Holliday MC, Baiqing D, Bhattacharya SK, Agtini MD (2008). A study of typhoid fever in five Asian countries: disease burden and implications for controls. Bull World Health Organ.

[63] Zhuang L, Zhang YJ, Tang Z, Dong C, Zhou L, Qian HM (2012). Epidemiologic characteristics of typhoid and paratyphoid fever on related drug resistance and molecular types regarding Salmonella Typhi and S. Paratyphi, in Jiangsu province [Chinese]. Chung-Hua Liu Hsing Ping Hsueh Tsa Chih Chin J Epidemiol.

[64] Kapil A, Renuka, Das B. Nalidixic acid susceptibility test to screen ciprofloxacin resistance in Salmonella Typhi. Indian J Med Res 2002;115(February):49–54. 12138664

[65] Capoor MR, Nair D, Hasan AS, Aggarwal P, Gupta B (2006). Typhoid fever: narrowing therapeutic options in India. Southeast Asian J Trop Med Public Health.

[66] Capoor MR, Nair D, Walia NS, Routela RS, Grover SS, Deb M (2009). Molecular analysis of high-level ciprofloxacin resistance in Salmonella enterica serovar Typhi and S. Paratyphi A: need to expand the QRDR region. Epidemiol Infect.

[67] Butt T, Ahmad RN, Salman M, Kazmi SY (2005). Changing trends in drug resistance among typhoid Salmonellae in Rawalpindi, Pakistan. Eastern Mediterranean Health J.

[68] Hasan R, Zafar A, Abbas Z, Mahraj V, Malik F, Zaidi A (2008). Antibiotic resistance among Salmonella enterica serovars Typhi and Paratyphi A in Pakistan (2001-2006). J Infect Dev Countries.

[69] Abdullah FE, Faryal H, Kanwal F, Saboohi I, Iqbal MS (2012). Enteric fever in Karachi: current antibiotic susceptibility of Salmonellae isolates. J College Physicians Surgeons Pakistan.

[70] Zehra NM, Irfan F, Mirza IA, Imtiaz A, Nadeem S, Hameed F (2017). Current trends of antimicrobial susceptibility of typhoidal Salmonellae isolated at tertiary care hospital. J Coll Physicians Surg Pak.

[71] Qureshi AH, Mushahid N, Ijaz A, Ahmad A, Ateque M, Marri MH (2001). Changing drug susceptibility pattern of Salmonellae Paratyphi A. J Coll Physicians Surg Pak.

[72] Punjabi NH, Agtini MD, Ochiai RL, Simanjuntak CH, Lesmana M, Subekti D (2013). Enteric fever burden in North Jakarta, Indonesia: a prospective, community-based study. J Infect Dev Ctries..

[73] Hardjo Lugito NP, Cucunawangsih (2017). Antimicrobial resistance of *Salmonella enterica* serovars Typhi and Paratyphi isolates from a general hospital in Karawaci, Tangerang, Indonesia: A five-year review. International Journal of Microbiol.

[74] Kuijpers LMF, Veng CH, Sar D, Chung P, Phe T, Kham C (2015). Ongoing outbreak of Salmonella enterica serovar Paratyphi A infections, Phnom Penh, Cambodia. J Infect Dev Ctries..

[75] Dimitrov T, Dashti AA, Albaksami O, Jadaon MM (2010). Detection of mutations in the gyrA gene in fluoroquinolone resistance Salmonella enterica serotypes Typhi and Paratyphi A isolated from the infectious diseases hospital. Kuwait J Clin Pathol.

[76] Elumalai S, Muthu G, Selvam RE, Ramesh S (2014). Detection of TEM-, SHV- and CTX-M-type beta-lactamase production among clinical isolates of Salmonella species. J Med Microbiol.

[77] Qamar FN, Asma A, Kazi AM, Erum K, Zaidi AKM (2014). A three-year review of antimicrobial resistance of Salmonella enterica serovars Typhi and Paratyphi A in Pakistan. J Infect Dev Ctries..

[78] Capoor MR, Deepthi N, Jitendra P, Smita S, Monorama D, Pushpa A (2009). Minimum inhibitory concentration of carbapenems and tigecycline against Salmonella spp. J Med Microbiol.

[79] Maheshwari VD, Agarwal SK (1996). Present status of drug resistance in cases of enteric fever in Rajasthan. J Assoc Physicians India.

[80] Mishra OP, Gupta BL, Nath G, Prakash J (1996). Treatment of multidrug-resistant typhoid fever. J Trop Pediatr.

[81] Bajracharya BL, Baral MR, Shakya S, Tuladhar P, Paudel M, Acharya B (2006). Clinical profile and antibiotics response in typhoid fever. Kathmandu Univ Med J.

[82] Mushtaq MA (2006). What after ciprofloxacin and ceftriaxone in treatment of Salmonella Typhi. Pakistan J Med Sci.

[83] Yu R, Liang J, Xu H (2010). Clinical analysis of 125 children with typhoid fever from 1993 to 2008 in Chongqing area. [Chinese]. Chinese J Pract Pediatr.

[84] Singh U, Neopane A, Thapa M, Aryal N, Agrawal K (2011). Salmonella Typhi infections and effect of fluroquinolones and third generation cephalosporins in clinical outcome. J Nepal Paediatric Soc.

[85] Narain U, Gupta R. Emergence of resistance in community-acquired enteric fever. Indian Pediatrics. 2015;(8):52, 709. 10.1007/s13312-015-0704-0 26388636

[86] Bhat KG, Tripathy A, Rajagopal R, Ramachandran S (2009). A simple broth-disk method to determine the minimum inhibitory concentration of ceftriaxone on Salmonella enterica serovar Typhi and Paratyphi. Indian J Pathol Microbiol.

[87] Afzal A, Sarwar Y, Ali A, Maqbool A, Salman M, Habeeb MA (2013). Molecular evaluation of drug resistance in clinical isolates of Salmonella enterica serovar Typhi from Pakistan. J Infect Dev Ctries.

[88] Gautam V, Gupta NK, Chaudhary U, Arora DR (2002). Sensitivity pattern of Salmonella serotypes in Northern India. Braz J Infect Dis.

[89] Kumar S, Rizvi M, Berry N (2008). Rising prevalence of enteric fever due to multidrug-resistant Salmonella: an epidemiological study. J Med Microbiol.

[90] Suruchi B, Anil K, Ganju SA, Atal S. Antibiotic susceptibility pattern of *Salmonella enterica* serovar Typhi and Paratyphi a from North India: the changing scenario. Int J Pharma Bio Sci. 2014;5(4):1-9.

[91] Makkar A, Gupta S, Khan ID, Gupta RM, Rajmohan KS, Chopra H (2018). Epidemiological profile and antimicrobial resistance pattern of enteric fever in a tertiary care hospital of North India - a seven year ambispective study. Acta Med (Hradec Kralove).

[92] Geetha VK, Yugendran T, Srinivasan R, Harish BN (2014). Plasmid-mediated quinolone resistance in typhoidal Salmonellae: a preliminary report from South India. Indian J Med Microbiol.

[93] Shetty AK, Shetty IN, Furtado ZV, Antony B, Boloor R (2012). Antibiogram of Salmonella isolates from blood with an emphasis on nalidixic acid and chloramphenicol susceptibility in a tertiary care hospital in coastal Karnataka: a prospective study. J Laboratory Physicians.

[94] British Society for Antimicrobial Chemotherapy. BSAC Methods for antimicrobial susceptibility testing. Version 11.1 May 2012. http://bsac.org.uk/wp-content/uploads/2012/02/Version-11.1-2012-Final-.pdf. Accessed 22 Nov 2018. 2012.

[95] Srirangaraj S, Kali A, Charles MV (2014). A study of antibiogram of Salmonella enterica serovar Typhi isolates from Pondicherry, India. Australas Med J.

[96] Rai S, Jain S, Prasad KN, Ghoshal U, Dhole TN (2012). Rationale of azithromycin prescribing practices for enteric fever in India. Indian J Med Microbiol.

[97] Ikram S, Hussain S, Aslam A, Khan MD, Ahmed I (2016). Evaluation of the current trends in the antimicrobial susceptibility patterns of typhoid salmonellae. Pakistan J Med Health Sci.

[98] Karkey A, Thwaites GE, Baker S (2018). The evolution of antimicrobial resistance in Salmonella Typhi. Curr Opin Gastroenterol.

[99] Rowe B, Ward LR, Threlfall EJ (1997). Multidrug-resistant Salmonella typhi: a worldwide epidemic. Clin Infect Dis.

[100] Pandit A, Arjyal A, Day JN, Paudyal B, Dangol S, Zimmerman MD (2007). An open randomized comparison of gatifloxacin versus cefixime for the treatment of uncomplicated enteric fever. PLoS ONE.

[101] Klemm EJ, Shakoor S, Page AJ, Qamar FN, Judge K, Saeed DK, et al. Emergence of an extensively drug-resistant *Salmonella enterica* serovar Typhi clone harboring a promiscuous plasmid encoding resistance to fluoroquinolones and third-generation cephalosporins. mBio. 2018;9(1):e00105-18. 10.1128/mBio.00105-18. 10.1128/mBio.00105-18 PMC5821095 29463654

[102] Thompson CN, Karkey A, Dongol S, Arjyal A, Wolbers M, Darton T (2017). Treatment response in enteric fever in an era of increasing antimicrobial resistance: an individual patient data analysis of 2092 participants enrolled into 4 randomized, controlled trials in Nepal. Clin Infect Dis.

[103] Hassing RJ, Goessens WH, van Pelt W, Mevius DJ, Stricker BH, Molhoek N (2014). Salmonella subtypes with increased MICs for azithromycin in travelers returned to The Netherlands. Emerg Infect Dis.

[104] Molloy A, Nair S, Cooke FJ, Wain J, Farrington M, Lehner PJ (2010). First report of Salmonella enterica serotype Paratyphi A azithromycin resistance leading to treatment failure. J Clin Microbiol.

[105] Rupali P, Abraham OC, Jesudason MV, John TJ, Zachariah A, Sivaram S (2004). Treatment failure in typhoid fever with ciprofloxacin susceptible Salmonella enterica serotype Typhi. Diagn Microbiol Infect Dis.

[106] Ahmed D, Nahid MA, Sami AB, Halim F, Akter N, Sadique T, et al. Bacterial etiology of bloodstream infections and antimicrobial resistance in Dhaka, Bangladesh, 2005-2014. Antimicrob Resist Infect Control. 2017;6:2. 10.1186/s13756-016-0162-z PMC5217397 28070309

[107] Rahman M, Siddique AK, Shoma S, Rashid H, Salam MA, Ahmed QS (2006). Emergence of multidrug-resistant Salmonella enterica serotype Typhi with decreased ciprofloxacin susceptibility in Bangladesh. Epidemiol Infect.

[108] Vlieghe ER, Phe T, De Smet B, Veng CH, Kham C, Bertrand S (2012). Azithromycin and ciprofloxacin resistance in Salmonella bloodstream infections in Cambodian adults. PLoS Negl Trop Dis.

[109] Holt KE, Dutta S, Manna B, Bhattacharya SK, Bhaduri B, Pickard DJ (2012). High-resolution genotyping of the endemic Salmonella Typhi population during a Vi (typhoid) vaccination trial in Kolkata. PLoS Negl Trop Dis.

[110] Kalckreutha VV, Koningsa F, Aaby P, Yaw A-S, Ali M, Aseffa A (2016). The Typhoid Fever Surveillance in Africa Program (TSAP): clinical, diagnostic, and epidemiological methodologies. (Special Issue: Typhoid fever surveillance in Africa program.). Clin Infect Dis.

[111] Molander V, Elisson C, Balaji V, Backhaus E, John J, Vargheese R (2013). Invasive pneumococcal infections in Vellore, India: clinical characteristics and distribution of serotypes. BMC Infect Dis.

[112] Williams PCM, Isaacs D, Berkley JA (2018). Antimicrobial resistance among children in sub-Saharan Africa. Lancet Infect Dis.

[113] Leopold SJ, van Leth F, Tarekegn H, Schultsz C (2014). Antimicrobial drug resistance among clinically relevant bacterial isolates in sub-Saharan Africa: a systematic review. J Antimicrob Chemother.

[114] Gupta K, Hooton TM, Naber KG, Wullt B, Colgan R, Miller LG (2011). International clinical practice guidelines for the treatment of acute uncomplicated cystitis and pyelonephritis in women: a 2010 update by the Infectious Diseases Society of America and the European Society for Microbiology and Infectious Diseases. Clin Infect Dis.

[115] Als D, Radhakrishnan A, Arora P, Gaffey MF, Campisi S, Velummailum R (2018). Global trends in typhoidal Salmonellosis: a systematic review. Am J Trop Med Hyg.

[116] Keddy KH, Smith AM, Sooka A, Tau NP, Ngomane HMP, Radhakrishnan A (2018). The burden of typhoid fever in South Africa: the potential impact of selected interventions. Am J Trop Med Hyg.

[117] Radhakrishnan A, Als D, Mintz ED, Crump JA, Stanaway J, Breiman RF (2018). Introductory article on global burden and epidemiology of typhoid fever. Am J Trop Med Hyg.

[118] Ashley EA, Lubell Y, White NJ, Turner P (2011). Antimicrobial susceptibility of bacterial isolates from community acquired infections in sub-Saharan Africa and Asian low and middle income countries. Trop Med Int Health.

[119] Tadesse BT, Ashley EA, Ongarello S, Havumaki J, Wijegoonewardena M, Gonzalez IJ (2017). Antimicrobial resistance in Africa: a systematic review. BMC Infect Dis.

[120] Bryce A, Hay AD, Lane IF, Thornton HV, Wootton M, Costelloe C (2016). Global prevalence of antibiotic resistance in paediatric urinary tract infections caused by Escherichia coli and association with routine use of antibiotics in primary care: systematic review and meta-analysis. BMJ..

[121] Vandenbroucke JP, von Elm E, Altman DG, Gotzsche PC, Mulrow CD, Pocock SJ (2007). Strengthening the Reporting of Observational Studies in Epidemiology (STROBE): explanation and elaboration. Epidemiology..

[122] Turner P, Fox-Lewis A, Shrestha P, Dance DAB, Wangrangsimakul T, Cusack TP (2019). Microbiology Investigation Criteria for Reporting Objectively (MICRO): a framework for the reporting and interpretation of clinical microbiology data. BMC Med.

[123] Britto CD, Wong VK, Dougan G, Pollard AJ (2018). A systematic review of antimicrobial resistance in Salmonella enterica serovar Typhi, the etiological agent of typhoid. PLoS Negl Trop Dis.

[124] Ochiai L, Khan MI, Sahastrabuddhe S, Wierzba T (2012). Epidemiology of typhoid fever. Int J Infect Dis.

[125] Teerawattanasook N, Tauran PM, Teparrukkul P, Wuthiekanun V, Dance DAB, Arif M (2017). Capacity and utilization of blood culture in two referral hospitals in Indonesia and Thailand. Am J Trop Med Hyg.

[126] Wong VK, Baker S, Pickard DJ, Parkhill J, Page AJ, Feasey NA (2015). Phylogeographical analysis of the dominant multidrug-resistant H58 clade of Salmonella Typhi identifies inter-and intracontinental transmission events. Nat Genet.

[127] Clinical and Laboratory Standards Institute (2010). Performance standards for antimicrobial susceptibility testing; twentieth informational supplement; M100-S20.

[128] Clinical and Laboratory Standards Institute (2015). Performance standards for antimicrobial susceptibility testing; twenty-fifth informational supplement. CLSI document M100-S25.

[129] Klein EY, Van Boeckel TP, Martinez EM, Pant S, Gandra S, Levin SA (2018). Global increase and geographic convergence in antibiotic consumption between 2000 and 2015. Proc Natl Acad Sci U S A.

[130] Tedijanto C, Olesen SW, Grad YH, Lipsitch M (2018). Estimating the proportion of bystander selection for antibiotic resistance among potentially pathogenic bacterial flora. Proc Natl Acad Sci U S A.

[131] O’Neill J. Tackling drug-resistant infections globally: final report and recommendations. The review on antimicrobial resistance 2016.

