# Supplementary material for: Drug-resistant enteric fever worldwide, 1990 to 2018: a systematic review and meta-analysis
Source: BMC Med. 2020 Jan 3;18:1. doi: 10.1186/s12916-019-1443-1 (PMC6941399; doi:10.1186/s12916-019-1443-1)

**Additional File 1:**

**Tables:**

Table S1. PRISMA Checklist adapted from Moher *et al.*

Table S2. Database search strategy.

Table S3. Number of included studies and isolates

Table S4. Sensitivity analysis

Table S5. Azithromycin resistance in *S.* Typhi.

Table S6. Studies incuded in the systematic review

**Figures:**

Figure S1. Metaanalysis and median prevalence comparison

Figure S2. Sensitivity analysis

Figure S3. MDR *S.* Typi in sub-Saharan Africa

Figure S4. MDR *S.* Typhi in NAME

Figure S5. MDR *S.* Typhi in East Asia

Figure S6. FQNS *S.* Typhi in sub-Saharan Africa

Figure S7. FQNS *S.* Typhi in NAME

Figure S8. FQNS *S.* Typhi in East Asia

Figure S9. MDR *S.* Paratypi in South Asia

Figure S10. MDR *S.* Paratyphi in Southeast Asia

Figure S11. MDR *S.* Paratyphi in East Asia

Figure S12. FQNS *S.* Paratypi in South Asia

Figure S13. FQNS *S.* Paratyphi in Southeast Asia

Figure S14. FQNS *S.* Paratyphi in East Asia

**Table S1. PRISMA Checklist:** adapted from Moher *et al.*

| **Section/topic** | **#** | **Checklist item** | **Reported** |
| --- | --- | --- | --- |
| **TITLE** | | |  |
| Title | 1 | Identify the report as a systematic review, meta-analysis, or both. | 🗹 |
| **ABSTRACT** | | |  |
| Structured summary | 2 | Provide a structured summary including, as applicable: background; objectives; data sources; study eligibility criteria, participants, and interventions; study appraisal and synthesis methods; results; limitations; conclusions and implications of key findings; systematic review registration number. | 🗹 |
| **INTRODUCTION** | | |  |
| Rationale | 3 | Describe the rationale for the review in the context of what is already known. | 🗹 |
| Objectives | 4 | Provide an explicit statement of questions being addressed with reference to participants, interventions, comparisons, outcomes, and study design (PICOS). | 🗹 |
| **METHODS** | | |  |
| Protocol and registration | 5 | Indicate if a review protocol exists, if and where it can be accessed (e.g., Web address), and, if available, provide registration information including registration number. | 🗹 |
| Eligibility criteria | 6 | Specify study characteristics (e.g., PICOS, length of follow-up) and report characteristics (e.g., years considered, language, publication status) used as criteria for eligibility, giving rationale. | 🗹 |
| Information sources | 7 | Describe all information sources (e.g., databases with dates of coverage, contact with study authors to identify additional studies) in the search and date last searched. | 🗹 |
| Search | 8 | Present full electronic search strategy for at least one database, including any limits used, such that it could be repeated. | 🗹 |
| Study selection | 9 | State the process for selecting studies (i.e., screening, eligibility, included in systematic review, and, if applicable, included in the meta-analysis). | 🗹 |
| Data collection process | 10 | Describe method of data extraction from reports (e.g., piloted forms, independently, in duplicate) and any processes for obtaining and confirming data from investigators. | 🗹 |
| Data items | 11 | List and define all variables for which data were sought (e.g., PICOS, funding sources) and any assumptions and simplifications made. | 🗹 |
| Risk of bias in individual studies | 12 | Describe methods used for assessing risk of bias of individual studies (including specification of whether this was done at the study or outcome level), and how this information is to be used in any data synthesis. | 🗹 |
| Summary measures | 13 | State the principal summary measures (e.g., risk ratio, difference in means). | 🗹 |
| Synthesis of results | 14 | Describe the methods of handling data and combining results of studies, if done, including measures of consistency (e.g., I^2^) for each meta-analysis. | 🗹 |
| Risk of bias across studies | 15 | Specify any assessment of risk of bias that may affect the cumulative evidence (e.g., publication bias, selective reporting within studies). | 🗹 |
| Additional analyses | 16 | Describe methods of additional analyses (e.g., sensitivity or subgroup analyses, meta-regression), if done, indicating which were pre-specified. | 🗹 |
| **RESULTS** | | |  |
| Study selection | 17 | Give numbers of studies screened, assessed for eligibility, and included in the review, with reasons for exclusions at each stage, ideally with a flow diagram. | 🗹 |
| Study characteristics | 18 | For each study, present characteristics for which data were extracted (e.g., study size, PICOS, follow-up period) and provide the citations. | 🗹 |
| Risk of bias within studies | 19 | Present data on risk of bias of each study and, if available, any outcome level assessment (see item 12). | 🗹 |
| Results of individual studies | 20 | For all outcomes considered (benefits or harms), present, for each study: (a) simple summary data for each intervention group (b) effect estimates and confidence intervals, ideally with a forest plot. | 🗹 |
| Synthesis of results | 21 | Present results of each meta-analysis done, including confidence intervals and measures of consistency. | 🗹 |
| Risk of bias across studies | 22 | Present results of any assessment of risk of bias across studies (see Item 15). | 🗹 |
| Additional analysis | 23 | Give results of additional analyses, if done (e.g., sensitivity or subgroup analyses, meta-regression [see Item 16]). | 🗹 |
| **DISCUSSION** | | |  |
| Summary of evidence | 24 | Summarize the main findings including the strength of evidence for each main outcome; consider their relevance to key groups (e.g., healthcare providers, users, and policy makers). | 🗹 |
| Limitations | 25 | Discuss limitations at study and outcome level (e.g., risk of bias), and at review-level (e.g., incomplete retrieval of identified research, reporting bias). | 🗹 |
| Conclusions | 26 | Provide a general interpretation of the results in the context of other evidence, and implications for future research. | 🗹 |
| **FUNDING** | | |  |
| Funding | 27 | Describe sources of funding for the systematic review and other support (e.g., supply of data); role of funders for the systematic review. | 🗹 |

**Table S2: Database search strategy.** Strategy designed in Ovid MedLine and altered for other databases

| **Search number** | **Search term** |
| --- | --- |
| 1 | Typhoid Fever/ (11123) |
| 2 | typhoid*.ti,ab. (12200) |
| 3 | paratyphoid*.ti,ab. (1674) |
| 4 | "enteric fever*".ti,ab. (1511) |
| 5 | "salmonella typhi".ti,ab. (4311) |
| 6 | "salmonella paratyphi".ti,ab. (821) |
| 7 | paratyphoid fever/ (2380) |
| 8 | 1 or 2 or 3 or 4 or 5 or 6 or 7(20567) |
| 9 | exp Drug Resistance, Bacterial/ (78649) |
| 10 | exp Microbial Sensitivity Tests/ (116858) |
| 11 | "antibacterial resistan*".ti,ab. (617) |
| 12 | "antibacterial drug resistan*".ti,ab. (33) |
| 13 | "bacterial drug resistan*".ti,ab. (208) |
| 14 | "antimicrobial resistan*".ti,ab. (14949) |
| 15 | "antibiotic drug resistan*".ti,ab. (67) |
| 16 | "bacterial resistan*".ti,ab. (5266) |
| 17 | "antibiotics resistan*".ti,ab. (634) |
| 18 | "bacterial surveillan*".ti,ab. (40) |
| 19 | "antibiotic surveillan*".ti,ab. (36) |
| 20 | "antibacterial surveillan*".ti,ab. (1) |
| 21 | "antimicrobial surveillan*".ti,ab. (393) |
| 22 | "antimicrobial susceptib*".ti,ab. (11401) |
| 23 | exp Bacteremia/ (27328) |
| 24 | bacteraemia*.ti,ab. (5772) |
| 25 | bacteremia*.ti,ab. (21957) |
| 26 | ("blood culture*" adj3 result*).ti,ab. (1319) |
| 27 | (antibacter* adj3 surveillan*).ti,ab. (29) |
| 28 | 9 or 10 or 11 or 12 or 13 or 14 or 15 or 16 or 17 or 18 or 19 or 20 or 21 or 22 or 23 or 24 or 25 or 26 or 27 (215387) |
| 29 | 8 and 28 (2202) |
| 30 | 29 (2202) |
| 31 | limit 29 to yr="1990 -Current" (1930) |

**Table S3a: Total number of included studies and isolates.** Displayed are the total number of studies and isolates included by GBD region and five-year time-period. *Some studies provide data on multiple sites and/or time periods therefore the total number of studies displayed here is higher than the total number of studies included in this review.

| **GBD Region** | **Number of studies (Number of isolates)** | | | | | | |
| --- | --- | --- | --- | --- | --- | --- | --- |
|  | **1990-1994** | **1995-1999** | **2000-2004** | **2005-2009** | **2010-2014** | **2015-2018** | **Total** |
| Andean Latin America | 1(97) | 0(0) | 0(0) | 0(0) | 1(33) | 0(0) | 2(130) |
| Australasia | 0(0) | 0(0) | 0(0) | 0(0) | 0(0) | 0(0) | 0(0) |
| Caribbean | 0(0) | 0(0) | 0(0) | 0(0) | 0(0) | 0(0) | 0(0) |
| Central Asia | 0(0) | 1(34) | 1(41) | 1(123) | 0(0) | 0(0) | 3(198) |
| Central Europe | 0(0) | 0(0) | 0(0) | 0(0) | 0(0) | 0(0) | 0(0) |
| Central Latin America | 0(0) | 0(0) | 0(0) | 0(0) | 0(0) | 0(0) | 0(0) |
| Central Sub-Saharan Africa | 0(0) | 0(0) | 1(29) | 2(410) | 3(363) | 0(0) | 6(802) |
| East Asia | 0(0) | 0(0) | 7(4,916) | 4(443) | 2(463) | 0(0) | 13(5,822) |
| Eastern Europe | 0(0) | 0(0) | 0(0) | 0(0) | 0(0) | 0(0) | 0(0) |
| Eastern Sub-Saharan Africa | 2(62) | 3(357) | 2(345) | 6(2,504) | 4(254) | 0(0) | 17(3,522) |
| High-income Asia Pacific | 0(0) | 0(0) | 0(0) | 0(0) | 0(0) | 0(0) | 0(0) |
| High-income North America | 0(0) | 0(0) | 0(0) | 0(0) | 0(0) | 0(0) | 0(0) |
| North Africa & Middle East | 4(643) | 5(1,218) | 4(1,044) | 3(996) | 0(0) | 2(69) | 18(3,970) |
| Oceania | 0(0) | 0(0) | 0(0) | 0(0) | 0(0) | 0(0) | 0(0) |
| South Asia | 57(13,851) | 35(23,253) | 59(19,130) | 60(14,388) | 64(25,886) | 13(2,937) | 288(99,445) |
| Southeast Asia | 5(905) | 12(2,279) | 6(3,795) | 7(421) | 8(1,233) | 1(46) | 39(8,679) |
| Southern Latin America | 0(0) | 0(0) | 0(0) | 0(0) | 0(0) | 0(0) | 0(0) |
| Southern Sub-Saharan Africa | 0(0) | 0(0) | 0(0) | 0(0) | 0(0) | 0(0) | 0(0) |
| Tropical Latin America | 0(0) | 0(0) | 0(0) | 0(0) | 0(0) | 0(0) | 0(0) |
| Western Europe | 0(0) | 0(0) | 0(0) | 0(0) | 0(0) | 0(0) | 0(0) |
| Western Sub-Saharan Africa | 0(0) | 4(193) | 3(513) | 7(446) | 7(592) | 1(35) | 22(1,779) |
| Total | 69(15,558) | 60(27,334) | 83(29,813) | 90(19,731) | 89(28,824) | 17(3,087) | *408(124,347) |

**Table S3b: Number of included studies and isolates for *S.* Typhi.** Displayed by GBD region and five-year time-period. *Some studies provide data on multiple sites and/or time periods therefore the total number of studies displayed here is higher than the total number of studies included in this review.

| **GBD Region** | **Number of studies (Number of isolates)** | | | | | | |
| --- | --- | --- | --- | --- | --- | --- | --- |
|  | **1990-1994** | **1995-1999** | **2000-2004** | **2005-2009** | **2010-2014** | **2015-2018** | **Total** |
| Andean Latin America | 0(0) | 0(0) | 0(0) | 0(0) | 1(33) | 0(0) | 1(33) |
| Australasia | 0(0) | 0(0) | 0(0) | 0(0) | 0(0) | 0(0) | 0(0) |
| Caribbean | 0(0) | 0(0) | 0(0) | 0(0) | 0(0) | 0(0) | 0(0) |
| Central Asia | 0(0) | 1(34) | 1(41) | 1(123) | 0(0) | 0(0) | 3(198) |
| Central Europe | 0(0) | 0(0) | 0(0) | 0(0) | 0(0) | 0(0) | 0(0) |
| Central Latin America | 0(0) | 0(0) | 0(0) | 0(0) | 0(0) | 0(0) | 0(0) |
| Central Sub-Saharan Africa | 0(0) | 0(0) | 1(29) | 2(410) | 3(363) | 0(0) | 6(802) |
| East Asia | 0(0) | 0(0) | 5(618) | 2(164) | 0(0) | 0(0) | 7(782) |
| Eastern Europe | 0(0) | 0(0) | 0(0) | 0(0) | 0(0) | 0(0) | 0(0) |
| Eastern Sub-Saharan Africa | 2(62) | 3(357) | 2(345) | 6(2,504) | 4(254) | 0(0) | 17(3,522) |
| High-income Asia Pacific | 0(0) | 0(0) | 0(0) | 0(0) | 0(0) | 0(0) | 0(0) |
| High-income North America | 0(0) | 0(0) | 0(0) | 0(0) | 0(0) | 0(0) | 0(0) |
| North Africa & Middle East | 4(643) | 5(1,218) | 4(1,004) | 3(996) | 0(0) | 2(69) | 18(3,930) |
| Oceania | 0(0) | 0(0) | 0(0) | 0(0) | 0(0) | 0(0) | 0(0) |
| South Asia | 57(13,136) | 31(17,472) | 55(12,726) | 55(10,530) | 62(19,581) | 13(2,274) | 273(75,719) |
| Southeast Asia | 5(761) | 12(2,260) | 6(3,728) | 7(421) | 7(689) | 1(46) | 38(7,905) |
| Southern Latin America | 0(0) | 0(0) | 0(0) | 0(0) | 0(0) | 0(0) | 0(0) |
| Southern Sub-Saharan Africa | 0(0) | 0(0) | 0(0) | 0(0) | 0(0) | 0(0) | 0(0) |
| Tropical Latin America | 0(0) | 0(0) | 0(0) | 0(0) | 0(0) | 0(0) | 0(0) |
| Western Europe | 0(0) | 0(0) | 0(0) | 0(0) | 0(0) | 0(0) | 0(0) |
| Western Sub-Saharan Africa | 0(0) | 4(176) | 3(513) | 7(431) | 7(570) | 1(35) | 22(1,725) |
| Total | 68(14,602) | 56(21,517) | 77(19,004) | 83(15,579) | 84(21,490) | 17(2,424) | *385(94,616) |

**Table S3c: Number of included studies isolates for *S.* Paratyphi A.** Displayed by GBD region and five-year time-period. *Some studies provide data on multiple sites and/or time periods therefore the total number of studies displayed here is higher than the total number of studies included in this review.

| **GBD Region** | **Number of studies (Number of isolates)** | | | | | | |
| --- | --- | --- | --- | --- | --- | --- | --- |
|  | **1990-1994** | **1995-1999** | **2000-2004** | **2005-2009** | **2010-2014** | **2015-2018** | **Total** |
| Andean Latin America | 1(97) | 0(0) | 0(0) | 0(0) | 0(0) | 0(0) | 1(97) |
| Australasia | 0(0) | 0(0) | 0(0) | 0(0) | 0(0) | 0(0) | 0(0) |
| Caribbean | 0(0) | 0(0) | 0(0) | 0(0) | 0(0) | 0(0) | 0(0) |
| Central Asia | 0(0) | 0(0) | 0(0) | 0(0) | 0(0) | 0(0) | 0(0) |
| Central Europe | 0(0) | 0(0) | 0(0) | 0(0) | 0(0) | 0(0) | 0(0) |
| Central Latin America | 0(0) | 0(0) | 0(0) | 0(0) | 0(0) | 0(0) | 0(0) |
| Central Sub-Saharan Africa | 0(0) | 0(0) | 0(0) | 0(0) | 0(0) | 0(0) | 0(0) |
| East Asia | 0(0) | 0(0) | 2(4,298) | 4(279) | 2(463) | 0(0) | 8(5,040) |
| Eastern Europe | 0(0) | 0(0) | 0(0) | 0(0) | 0(0) | 0(0) | 0(0) |
| Eastern Sub-Saharan Africa | 0(0) | 0(0) | 0(0) | 0(0) | 0(0) | 0(0) | 0(0) |
| High-income Asia Pacific | 0(0) | 0(0) | 0(0) | 0(0) | 0(0) | 0(0) | 0(0) |
| High-income North America | 0(0) | 0(0) | 0(0) | 0(0) | 0(0) | 0(0) | 0(0) |
| North Africa & Middle East | 0(0) | 0(0) | 1(40) | 0(0) | 0(0) | 0(0) | 1(40) |
| Oceania | 0(0) | 0(0) | 0(0) | 0(0) | 0(0) | 0(0) | 0(0) |
| South Asia | 5(715) | 9(5,781) | 28(6,404) | 30(3,858) | 40(6,305) | 7(663) | 119(23,726) |
| Southeast Asia | 3(144) | 1(19) | 2(67) | 0(0) | 6(544) | 0(0) | 12(774) |
| Southern Latin America | 0(0) | 0(0) | 0(0) | 0(0) | 0(0) | 0(0) | 0(0) |
| Southern Sub-Saharan Africa | 0(0) | 0(0) | 0(0) | 0(0) | 0(0) | 0(0) | 0(0) |
| Tropical Latin America | 0(0) | 0(0) | 0(0) | 0(0) | 0(0) | 0(0) | 0(0) |
| Western Europe | 0(0) | 0(0) | 0(0) | 0(0) | 0(0) | 0(0) | 0(0) |
| Western Sub-Saharan Africa | 0(0) | 1(17) | 0(0) | 1(15) | 2(22) | 0(0) | 4(54) |
| Total | 9(956) | 11(5,817) | 33(10,809) | 35(4,152) | 50(7,334) | 7(663) | *145(29,731) |

**Table S4a. Sensitivity analysis:** Random effects metaanalysis results for studies on MDR *S.* Typhi in South Asia, comparing results for all studies with only those reporting (a) AST methods used; (b) resistance breakpoints used; (c) internal control strain used and (d) those on over 50 isolates of *S.* Typhi.

| **Time Period** | **Studies (N)** | ***I****²* (%) | **Pooled prevalence [95% CI]** |
| --- | --- | --- | --- |
| **All studies** | | | |
| 1990_1994 | 33 | 98% | 63% [54-71] |
| 1995_1999 | 14 | 100% | 38% [24-52] |
| 2000_2004 | 33 | 99% | 34% [24-44] |
| 2005_2009 | 31 | 99% | 12% [6-20] |
| 2010_2014 | 42 | 98% | 7% [4-12] |
| **Studies reporting AST methods** | | | |
| 1990-1994 | 25 | 99% | 61% [50-71] |
| 1995-1999 | 12 | 100% | 36% [22-51] |
| 2000-2004 | 27 | 99% | 32% [21-44] |
| 2005-2009 | 31 | 99% | 12% [6-20] |
| 2010-2014 | 39 | 98% | 6% [3-10] |
| **Studies reporting resistance breakpoints*** | | | |
| 1990-1994 | 5 | 100% | 67% [38-91] |
| 1995-1999 | 7 | 100% | 29% [14-47] |
| 2000-2004 | 20 | 99% | 27% [18-38] |
| 2005-2009 | 25 | 99% | 13% [6-22] |
| 2010-2014 | 38 | 98% | 6% [3-10] |
| **Studies reporting internal quality control** | | | |
| 1990-1994 | 33 | 98% | 63% [54-71] |
| 1995-1999 | 14 | 100% | 37% [23-53] |
| 2000-2004 | 33 | 99% | 34% [24-44] |
| 2005-2009 | 31 | 99% | 12% [6-20] |
| 2010-2014 | 42 | 98% | 7% [4-12] |
| **Studies with over 50 isolates** | | | |
| 1990-1994 | 23 | 98% | 61% [51-70] |
| 1995-1999 | 14 | 100% | 38% [24-52] |
| 2000-2004 | 22 | 99% | 34% [22-47] |
| 2005-2009 | 27 | 99% | 10% [4-17] |
| 2010-2014 | 30 | 99% | 8% [4-14] |

* Testing standards including version or year

**Table S4b. Sensitivity analysis:** Random effects metaanalysis results for studies on FQNS *S.* Typhi in South Asia, comparing results for all studies with only those reporting (a) AST methods used; (b) resistance breakpoints used; (c) internal control strain used and (d) those on over 50 isolates of *S.* Typhi.

| **Time Period** | **Studies (N)** | ***I****²* (%) | **Pooled prevalence [95% CI]** |
| --- | --- | --- | --- |
| **All studies** | | | |
| 1990_1994 | 6 | 93% | 2% [0-14] |
| 1995_1999 | 8 | 98% | 49% [26-72] |
| 2000_2004 | 29 | 99% | 62% [47-77] |
| 2005_2009 | 38 | 97% | 76% [69-83] |
| 2010_2014 | 50 | 99% | 82% [75-89] |
| **Studies reporting AST methods** | | | |
| 1990-1994 | 3 | 95% | 10% [0-46] |
| 1995-1999 | 8 | 98% | 49% [26-72] |
| 2000-2004 | 26 | 99% | 59% [42-74] |
| 2005-2009 | 36 | 97% | 77% [70-84] |
| 2010-2014 | 48 | 99% | 82% [74-89] |
| **Studies reporting resistance breakpoints*** | | | |
| 1990-1994 | 1 | 0% | 0% [0-0] |
| 1995-1999 | 6 | 99% | 51% [21-81] |
| 2000-2004 | 22 | 99% | 68% [53-82] |
| 2005-2009 | 31 | 97% | 77% [69-84] |
| 2010-2014 | 45 | 99% | 83% [74-90] |
| **Studies with control strain stated** | | | |
| 1990-1994 | 6 | 93% | 3% [0-15] |
| 1995-1999 | 8 | 98% | 49% [26-72] |
| 2000-2004 | 29 | 99% | 62% [47-77] |
| 2005-2009 | 38 | 97% | 76% [69-83] |
| 2010-2014 | 50 | 99% | 82% [75-89] |
| **Studies with over 50 isolates** | | | |
| 1990-1994 | 2 | 85% | 1% [0-4] |
| 1995-1999 | 6 | 96% | 39% [23-55] |
| 2000-2004 | 20 | 99% | 65% [48-80] |
| 2005-2009 | 26 | 98% | 75% [66-83] |
| 2010-2014 | 29 | 99% | 82% [72-91] |

* Testing standards including version or year

**Table S5: Azithromycin resistance in *S.* Typhi.** Resistant isolates are defined as having an MIC >16μg/ml [EUCAST 2014, BSAC 2012] or by disk diffusion, a zone diameter ≤18mm [BSAC 2012]. Sensitive isolates have an MIC ≤16μg/ml or zone diameter ≥19mm.

| **Author (Year)** | **Country** | **Testing Method** | **Number examined (N)** | **Resistant, N (%)** |
| --- | --- | --- | --- | --- |
| Chinh (2000) | Viet Nam | Agar dilution | 86 | 0 (0) |
| Capoor (2009) | India | E-test | 149 | 1 (1) |
| Kasper (2010) | Cambodia | E-test | 41 | 0 (0) |
| Ngoun (2012) | Cambodia | E-test | 102 | 0 (0) |
| Rai (2012)ᵇ | India | E-test | 80 | 27 (34) |
| Vlieghe (2012) | Cambodia | E-test | 20 | 1 (5) |
| Garg (2013) | India | E-test | 17 | 3 (7) |
| Jain (2013) | India | Disk-diffusion | 266 | 17 (6) |
| Koirala (2013) | Nepal | E-test | 218 | 0 (0) |
| Venkatesh (2013) | India | E-test | 132 | 0 (0) |
| Dutta (2014) | India | E-test | 77 | 0 (0) |
| Maltha (2014) | Burkina Faso | E-test | 12 | 0 (0) |
| Phoba (2014) | DRC | E-test | 18 | 0 (0) |
| Srirangaraj (2014) | India | E-test | 16 | 2 (13) |
| Ikram (2015) | Pakistan | Disk-diffusion | 71 | 60 (85) |
| Chande (2016) | India | E-test | 108 | 0 (0) |
| Iyer (2017) | India | E-test | 217 | 0 (0) |
| Khanal (2017) | Nepal | Agar dilution | 74 | 0 (0) |
| Kuijpers (2017) | Cambodia | E-test | 64 | 0 (0) |
| Okanda (2018) | Bangladesh | Microdilution | 18 | 0 (0) |
| Sharma (2018) | India | E-test | 95 | 1 (1) |
| Joshi (2019) | India | E-test | 300 | 0 (0) |

**Table S6: Studies included in the systematic review**

| **Author (Year)** | **Citation** |
| --- | --- |
| Aatekah (2010) | Aatekah O, Shazia S, Umber Z, Arjumand R, Zaidi AKM. Incidence of typhoid bacteremia in infants and young children in southern coastal Pakistan. *Pediatr Infect Dis J* 2010; 29(11): 1035-9. |
| Abdel Wahab (1999) | Abdel Wahab MF, el-Gindy IM, Sultan Y, el-Naby HM. Comparative study on different recent diagnostic and therapeutic regimens in acute typhoid fever. *J Egypt Public Health Assoc* 1999; 74(1-2): 193-205. |
| Abdullah (2012) | Abdullah FE, Faryal H, Kanwal F, Saboohi I, Iqbal MS. Enteric fever in Karachi: current antibiotic susceptibility of Salmonellae isolates. *J Coll Physicians Surg Pak* 2012; 22(3): 147-50. |
| Abdullah (2013) | Abdullah MA, Adnan Z, Sattar NY. Susceptibility of Salmonella enterica serotype Typhi, to the usual line of antimicrobial treatment in Rawalpindi. *Pakistan Journal of Public Health* 2013; 3(2): 14-8. |
| Abdullahi (2015) | Abdullahi M, Olonitola SO, Umoh VJ, Inabo IH. Antibacterial resistance profile and PCR detection of antibiotic resistance genes in Salmonella serovars isolated from blood samples of hospitalized subjects in Kano, North-West, Nigeria. *Br Microbiol Res J* 2015; 5(3): 245-56. |
| Abucejo (2001) | Abucejo PE, Capeding MR, Lupisan SP, et al. Blood culture confirmed typhoid fever in a provincial hospital in the Philippines. *Southeast Asian J Trop Med Public Health* 2001; 32(3): 531-6. |
| Acharya (1995) | Acharya G, Butler T, Ho M, et al. Treatment of typhoid fever: randomized trial of a three-day course of ceftriaxone versus a fourteen-day course of chloramphenicol. *Am J Trop Med Hyg* 1995; 52(2): 162-5. |
| Acharya (2011) | Acharya D, Bhatta DR, Malla S, Dumre SP, Adhikari N, Kandel BP. Salmonella enterica serovar Paratyphi A: an emerging cause of febrile illness in Nepal. *Nepal Med Coll J* 2011; 13(2): 69-73. |
| Acharya (2012) | Acharya D, Trakulsomboon S, Madhup SK, Korbsrisate S. Antibiotic susceptibility pattern and the indicator of decreased ciprofloxacin susceptibility of Salmonella enterica serovar Typhi isolated from Dhulikhel Hospital, Nepal. *Jpn J Infect Dis* 2012; 65(3): 264-7. |
| Achla (2005) | Achla P, Grover SS, Bhatia R, Khare S. Sensitivity index of antimicrobial agents as a simple solution for multidrug resistance in Salmonella Typhi. *Indian J Med Res* 2005; 121(3): 185-93. |
| Adeshina (2009) | Adeshina GO, Osuagwu NO, Okeke CLE, Ehinmidu JO, Bolaji RO. Prevalence and susceptibility of Salmonella Typhi and Salmonella Paratyphi in Zaria, Nigeria. *International Journal of Health Research* 2009; 2(4): 355-60. |
| Adhikari (2012) | Adhikari D, Acharya D, Shrestha P, Amatya R. Ciprofloxacin susceptibility of Salmonella enteric serovar Typhi and Paratyphi A from blood samples of suspected enteric fever patients. *International Journal of Infection and Microbiology* 2012; 1(1): 9-13. |
| Afia (2005) | Afia Z, Ibrahim NG, Tanwir A, Zohair A, Zaidi A, Rumina H. Nalidixic acid screening test in detection of decreased fluoroquinolone susceptibility in Salmonella Typhi isolated from blood. *J Coll Physicians Surg Pak* 2005; 15(7): 413-7. |
| Afifi (2005) | Afifi S, Earhart K, Azab MA, et al. Hospital-based surveillance for acute febrile illness in Egypt: a focus on community-acquired bloodstream infections. *Am J Trop Med Hyg* 2005; 73(2): 392-9. |
| Afroze (2014) | Afroze SR, Rahim MA, Hasan MM, et al. Pattern of antibiotic sensitivity in enteric fever: A tertiary care hospital experience. *Journal of Medicine (Bangladesh)* 2014; 15(2): 122-4. |
| Afzal (2013) | Afzal A, Sarwar Y, Ali A, et al. Molecular evaluation of drug resistance in clinical isolates of Salmonella enterica serovar Typhi from Pakistan. *J Infect Dev Ctries* 2013; 7(12): 929-40. |
| Agarwal (1991) | Agarwal S, Madhu SV, Guleria JS, Talwar V. The problem of emerging chloramphenicol resistance in typhoid fever - A preliminary report. *J Assoc Physicians India* 1991; 39(6): 443-4. |
| Aggarwal (2007) | Aggarwal A, Vij AS, Oberoi A. A three-year retrospective study on the prevalence, drug susceptibility pattern, and phage types of Salmonella enterica subspecies Typhi and Paratyphi in Christian Medical College and Hospital, Ludhiana, Punjab. *J Indian Acad Clin Med* 2007; 8(1): 32-5. |
| Aggarwal (2011) | Aggarwal A, Ghosh A, Gomber S, Mitra M, Parikh AO. Efficacy and safety of azithromycin for uncomplicated typhoid fever: an open label non-comparative study. *Indian Pediatr* 2011; 48(7): 553-6. |
| Agyekum (2010) | Agyekum A. Prevalence of bacteria causing bacteraemia in children under five years in Agogo, Asante-Akyem and their antimicrobial susceptibility patterns: Kwame Nkrumha University of Science & Technology, Kumasi; 2010. |
| Ahmed (2017) | Ahmed D, Nahid MA, Sami AB, et al. Bacterial etiology of bloodstream infections and antimicrobial resistance in Dhaka, Bangladesh, 2005-2014. *Antimicrob Resist Infect Control* 2017; 6. |
| Akinyemi (2000) | Akinyemi KO, Coker AO, Olukoya DK, Oyefolu AO, Amorighoye EP, Omonigbehin EO. Prevalence of multi-drug resistant Salmonella Typhi among clinically diagnosed typhoid fever patients in Lagos, Nigeria. *Z Naturforsch [C]* 2000; 55(5-6): 489-93. |
| Akinyemi (2005) | Akinyemi KO, Smith SI, Bola Oyefolu AO, Coker AO. Multidrug resistance in Salmonella enterica serovar typhi isolated from patients with typhoid fever complications in Lagos, Nigeria. *Public Health* 2005; 119(4): 321-7. |
| Akinyemi (2015) | Akinyemi KO, Iwalokun BA, Alafe OO, Mudashiru SA, Fakorede C. Bla<inf>CTX-M-I</inf> group extended spectrum beta lactamase-producing Salmonella Typhi from hospitalized patients in Lagos, Nigeria. *Infect Drug Resist* 2015; 8: 99-106. |
| Akinyemi (2018) | Akinyemi KO, Oyefolu AOB, Mutiu WB, et al. Typhoid fever: tracking the trend in Nigeria. (Special Issue: Tackling typhoid - what do global and country trends teach us?).American Journal of Tropical Medicine and Hygiene 2018;99(3 Suppl):41-47 |
| Al-Abbasy (2018) | Al-Abbasy AJ. Molecular study of antibiotic resistance gene in salmonella enterica serovar typhi isolates.International Journal of Pharmaceutical Research 2018;10(3):378-384 |
| Alam (2011) | Alam MS, Pillai PK, Kapur P, Pillai KK. Resistant patterns of bacteria isolated from bloodstream infections at a university hospital in Delhi. *J Pharm Bioallied Sci* 2011; 3(4): 525-30. |
| Alaullah (2009) | Alaullah S, Bhuiyan MS, Farhana K, et al. Salmonella enterica serovar Typhi-specific immunoglobulin A antibody responses in plasma and antibody in lymphocyte supernatant specimens in Bangladeshi patients with suspected typhoid fever. *Clin Vaccine Immunol* 2009; 16(11): 1587-94. |
| Ali (2010) | Ali SQ, Ale Z, Naqvi BS, Shahjahan S, Rabia B. Resistance pattern of ciprofloxacin against different pathogens. *Oman Med J* 2010; 25(4): 294-8. |
| Ali (2016) | Ali MK, Sultana S. Antimicrobial sensitivity patterns of Salmonella Typhi in children. *Bangladesh J Med Sci* 2016; 15(3): 416-8. |
| Ali (2017) | Ali A, Ali HA, Shah FH, Zahid A, Aslam H, Javed B. Pattern of antimicrobial drug resistance of Salmonella Typhi and Paratyphi a in a teaching hospital in Islamabad. *J Pak Med Assoc* 2017; 67(3): 375-9. |
| Aliya (2010) | Aliya N, Ram PK, Brooks WA, et al. Burden of typhoid and paratyphoid fever in a densely populated urban community, Dhaka, Bangladesh. *Int J Infect Dis* 2010; 14(Suppl. 3): e93-e9. |
| Aljanaby (2017) | Aljanaby AAJ, Medhat AR. Prevalence of some antimicrobials resistance associated-genes in Salmonella Typhi isolated from patients infected with typhoid fever. *J Bio Sci* 2017; 17(4): 171-84. |
| Amatya (2007) | Amatya NM, Shrestha B, Lekhak B. Etiological agents of bacteraemia and antibiotic susceptibility pattern in Kathmandu Model Hospital. *JNMA J Nepal Med Assoc* 2007; 46(167): 112-8. |
| Amdani (1998) | Amdani SK. Antibiotic resistance pattern of pediatric typhoid fever patients at Harapan Kita Children and Maternity Hospital Jakarta, 1996. *Med J Indones* 1998; 7: 253-6. |
| Anand (1993) | Anand AC. The anatomy of an epidemic (the final report on an epidemic of multidrug resistant enteric fever in eastern India). *Trop Gastroenterol* 1993; 14(1): 21-7. |
| Andrews (2018) | Andrews JR, Qamar FN, Charles RC and Ryan ET. Extensively Drug-Resistant Typhoid - Are Conjugate Vaccines Arriving Just in Time?New England Journal of Medicine 2018;379(16):1493-1495 |
| Anees (2015) | Anees A, Indu S, Fatima K, Anjum P. Multi-drug resistant Salmonella enterica subspecies enterica serotype Typhi: A diagnostic and therapeutic challenge. *Int J Curr Microbiol Appl Sci* 2015; 4(Special Issue 1): 19-25. |
| Anglaret (1997) | Anglaret X, Sylla-Koko F, Bonard D, et al. Susceptibilities to co-trimoxazole of pathogens isolated from blood and stool specimens in Abidjan, Ivory Coast, 1994 to 1996. *J Clin Microbiol* 1997; 35(7): 1915. |
| Anjum (2004) | Anjum P, Qureshi AH, Rafi S. Fluroquinolone resistance in typhoidal Salmonella and its detection by nalidixic acid disc diffusion. *J Pak Med Assoc* 2004; 54(6): 295-301. |
| Arjyal (2011) | Arjyal A, Basnyat B, Koirala S, et al. Gatifloxacin versus chloramphenicol for uncomplicated enteric fever: an open-label, randomised, controlled trial. *Lancet Infect Dis* 2011; 11(6): 445-54. |
| Arjyal (2016) | Arjyal A, Basnyat B, Nhan HT, et al. Gatifloxacin versus ceftriaxone for uncomplicated enteric fever in Nepal: an open-label, two-centre, randomised controlled trial. *Lancet Infect Dis* 2016; 16(5): 535-45. |
| ARSP Working Group (2013) | ARSP Working Group. A multi centre laboratory study of Gram negative bacterial blood stream infections in Sri Lanka. *Ceylon Med J* 2013; 58(2): 56-61. |
| Asna (2000) | Asna SMZH, Ashraful Haq J. Decrease of antibiotic resistance in Salmonella Typhi isolated from patients attending hospitals of Dhaka City over a 3 year period. *Int J Antimicrob Agents* 2000; 16(3): 249-51. |
| Bajracharya (2006) | Bajracharya BL, Baral MR, Shakya S, Tuladhar P, Paudel M, Acharya B. Clinical profile and antibiotics response in typhoid fever. *Kathmandu Univ Med J (KUMJ)* 2006; 4(1): 25-9. |
| Baliga (1999) | Baliga S, Shenoy S, Vidyalaxmi, Pereira P. Ciprofloxacin-resistant Salmonella Typhi. *Natl Med J India* 1999; 12(3): 138. |
| Bavdekar (1991) | Bavdekar A, Chaudhari M, Bhave S, Pandit A. Ciprofloxacin in typhoid fever. *Indian J Pediatr* 1991; 58(3): 335-9. |
| Beig (2009) | Beig FK, Ahmad F, Abqari S. Changing antibiotic sensitivity pattern and scope of chloramphenicol in the management of hospitalised patients of typhoid fever. *J Indian Med Assoc* 2009; 107(12): 862-5. |
| Bekur (2010) | Bekur R, Vandana KE, Shivashankara KN, Valsalan R, Sathyanarayanan V. Paratyphoid fever- Emerging problem in South India. *Asian Pac J Trop Med* 2010; 3(10): 815-7. |
| Bello (2018) | Bello N, Kudu ATD, Adetokun AB, et al. Characterization and Antimicrobial Susceptibility Profile of Bacteraemia Causing Pathogens Isolated from Febrile Children with and without Sickle Cell Disease in Kano, Nigeria.Mediterranean Journal of Hematology & Infectious Diseases 2018;10(1):e2018016 |
| Bhaswati (2007) | Bhaswati S, Shanta D, Dipika S, et al. Phage typing, biotyping & antimicrobial resistance profile of Salmonella enterica serotype Typhi from Kolkata. *Indian J Med Res* 2007; 125(5): 685-8. |
| Bhat (1998) | Bhat KG, Andrade AT, Karadesai SG, Hemashettar BM, Patil CS. Antimicrobial susceptibility of Salmonella Typhi to quinolones & cephalosporins. *Indian J Med Res* 1998; 107: 247-51. |
| Bhat (1999) | Bhat KG, Suresh K. Ciprofloxacin-resistant Salmonella Typhi. *Natl Med J India* 1999; 12(2): 88. |
| Bhat (2009) | Bhat KG, Tripathy A, Rajagopal R, Ramachandran S. A simple broth-disk method to determine the minimum inhibitory concentration of ceftriaxone on Salmonella enterica serovar Typhi and Paratyphi. *Indian J Pathol Microbiol* 2009; 52(2): 189-90. |
| Bhatia (2007) | Bhatia JK, Mathur AD, Arora MM. Reemergence of chloramphenicol sensitivity in enteric fever. *Med J Armed Forces India* 2007; 63(3): 212-4. |
| Bhattacharya (1992) | Bhattacharya SK, Dutta D, Bhattacharya MK, et al. Multi-resistant typhoid fever. *Natl Med J India* 1992; 5(1): 41. |
| Bhattacharya (2003) | Bhattacharya SS, Das U. A steady decrease in occurrence of Salmonella Typhi infection in Rourkela, Orissa. *Indian J Pathol Microbiol* 2003; 46(3): 498-500. |
| Bhattacharya (2011) | Bhattacharya SS, Das U, Choudhury BK. Occurrence & antibiogram of Salmonella Typhi & S. Paratyphi A isolated from Rourkela, Orissa. *Indian J Med Res* 2011; 133: 431-3. |
| Bhetwal (2017) | Bhetwal A, Maharjan A, Khanal PR and Parajuli NP. Enteric Fever Caused by Salmonella enterica Serovars with Reduced Susceptibility of Fluoroquinolones at a Community Based Teaching Hospital of Nepal.International Journal of Microbiology 2017;2017(2869458 |
| Bisso Andrade (1998) | Bisso Andrade A. Fiebre paratífica: estudio de 107 pacientes. *Bol Soc Peru Med Interna* 1998; 11(4): 162-9. |
| Blacksell (2007) | Blacksell SD, Sharma NP, Phumratanaprapin W, et al. Serological and blood culture investigations of Nepalese fever patients. *Trans R Soc Trop Med Hyg* 2007; 101(7): 686-90. |
| Bouzenoune (2011) | Bouzenoune F, Debbih KK, Boudersa F, Kouhil S, Nezzar N. Antibiotic susceptibility of Salmonella enterica serovar Typhi isolated from blood cultures at the Ain M'lila hospital (Algeria), between 2005 and 2008. [French]. *Med Mal Infect* 2011; 41(4): 181-5. |
| Breiman (2012) | Breiman RF, Cosmas L, Njuguna H, et al. Population-based incidence of typhoid fever in an urban informal settlement and a rural area in Kenya: implications for typhoid vaccine use in Africa. *PLoS ONE* 2012; 7(1): e29119. |
| Britto (2018) | Britto CD, Dyson ZA, Duchene S, et al. Laboratory and molecular surveillance of paediatric typhoidal Salmonella in Nepal: Antimicrobial resistance and implications for vaccine policy.PLoS Neglected Tropical Diseases [electronic resource] 2018;12(4):e0006408 |
| Brooks (2005) | Brooks WA, Hossain A, Goswami D, et al. Bacteremic typhoid fever in children in an urban slum, Bangladesh. *Emerg Infect Dis* 2005; 11(2): 326-9. |
| Buch (1994) | Buch NA, Hassan MU, Kakroo DK. Enteric fever--a changing sensitivity pattern, clinical profile and outcome. *Indian Pediatr* 1994; 31(8): 981-5. |
| Butler (1993) | Butler T, Ho M, Acharya G, Tiwari M, Gallati H. Interleukin-6, gamma interferon, and tumor necrosis factor receptors in typhoid fever related to outcome of antimicrobial therapy. *Antimicrob Agents Chemother* 1993; 37(11): 2418-21. |
| Butt (2005) | Butt T, Ahmad RN, Salman M, Kazmi SY. Changing trends in drug resistance among typhoid Salmonellae in Rawalpindi, Pakistan. *East Mediterr Health J* 2005; 11(5-6): 1038-44. |
| Butt (2006) | Butt T, Khan MY, Ahmad RN, Salman M, Afzal RK. Validity of nalidixic acid screening in fluoroquinolone-resistant typhoid Salmonellae. *J Coll Physicians Surg Pak* 2006; 16(1): 31-4. |
| Capoor (2006) | Capoor MR, Nair D, Hasan AS, Aggarwal P, Gupta B. Typhoid fever: narrowing therapeutic options in India. *Southeast Asian J Trop Med Public Health* 2006; 37(6): 1170-4. |
| Capoor (2009) | Capoor MR, Deepthi N, Jitendra P, et al. Minimum inhibitory concentration of carbapenems and tigecycline against Salmonella spp. *J Med Microbiol* 2009; 58(3): 337-41. |
| Chakravorty (1993) | Chakravorty B, Jain N, Gupta B, Rajvanshi P, Sen MK, Krishna A. Chloramphenicol resistant enteric fever. *J Indian Med Assoc* 1993; 91(1): 10-3. |
| Chand (2014) | Chand HJ, Rijal KR, Neupane B, Sharma VK, Jha B. Re-emergence of susceptibility to conventional first line drugs in Salmonella isolates from enteric fever patients in Nepal. *J Infect Dev Ctries* 2014; 8(11): 1483-7. |
| Chandane (2017) | Chandane P, Gandhi A, Bowalekar S. Study of antibiotic susceptibility pattern of Salmonella Typhi in children suffering from enteric fever. *Ann Trop Med Public Health* 2017; 10(2): 440-3. |
| Chande (2002) | Chande C, Shrikhande S, Kapale S, Agrawal S, Fule RP. Change in antimicrobial resistance pattern of Salmonella Typhi in central India. *Indian J Med Res* 2002; 115: 248-50. |
| Chande (2016) | Chande CA, Chopdekar KA, Pradnya V, et al. Current trend of antibiotic sensitivity of Salmonella Typhi and other Salmonellae in Mumbai: A 5 years study. *Indian J Med Microbiol* 2016; 34(1): 115-6. |
| Chaudhary (2011) | Chaudhary R, Sijapati K, Singh SK. A study on nalidixic acid resistant Salmonella among the patients attending Shree Birendra Hospital. *Med J Shree Birendra Hosp* 2011; 10(2): 1-3. |
| Chheng (2013) | Chheng K, Carter MJ, Emary K, et al. A prospective study of the causes of febrile illness requiring hospitalization in children in Cambodia. *PLoS ONE* 2013; 8(4): e60634. |
| Chinh (1997) | Chinh NT, Solomon T, Thong MX, et al. Short courses of ofloxacin for the treatment of enteric fever. *Trans R Soc Trop Med Hyg* 1997; 91(3): 347-9. |
| Chinh (2000) | Chinh NT, Parry CM, Ly NT, et al. A randomized controlled comparison of azithromycin and ofloxacin for treatment of multidrug-resistant or nalidixic acid-resistant enteric fever. *Antimicrob Agents Chemother* 2000; 44(7): 1855-9. |
| Chowta (2005) | Chowta MN, Chowta NK. Study of clinical profile and antibiotic response in typhoid fever. *Indian J Med Microbiol* 2005; 23(2): 125-7. |
| Ciraj (1999) | Ciraj AM, Seetha KS, Gopalkrishna BK, Shivananda PG. Drug resistance pattern and phage types of Salmonella Typhi isolates in Manipal, South Karnataka. *Indian J Med Sci* 1999; 53(11): 486-9. |
| Ciraj (2000) | Ciraj AM, Mohammed M, Bhat KG, Shivananda PG. Copper resistance and its correlation to multiple drug resistance in Salmonella Typhi isolates from south Karnataka. *Indian J Med Res* 2000; 110(DEC.): 181-2. |
| Ciraj (2001) | Ciraj AM, Seema DS, Bhat GK, Shivananda PG. Nalidixic acid screening test for the detection of decreased susceptibility to ciprofloxacin in Salmonella Typhi. *Indian J Pathol Microbiol* 2001; 44(4): 407-8. |
| Crump (2011) | Crump JA, Ramadhani HO, Morrissey AB, et al. Invasive bacterial and fungal infections among hospitalized HIV-infected and HIV-uninfected adults and adolescents in northern Tanzania. *Clin Infect Dis* 2011; 52(3): 341-8. |
| Daga (1994) | Daga MK, Sarin K, Sarkar R. A study of culture positive multidrug resistant enteric fever--changing pattern and emerging resistance to ciprofloxacin. *J Assoc Physicians India* 1994; 42(8): 599-600. |
| Dar (1992) | Dar L, Gupta BL, Rattan A, Bhujwala RA, Shriniwas. Multidrug resistant Salmonella Typhi in Delhi. *Indian J Pediatr* 1992; 59(2): 221-4. |
| Das (2000) | Das U, Bhattacharya SS. Multidrug resistant Salmonella Typhi in Rourkela, Orissa. *Indian J Pathol Microbiol* 2000; 43(2): 135-8. |
| Das (2006) | Das U, Bhattacharya SS. Antibiogram, phage typing and biotyping of Salmonella Typhi and Salmonella Paratyphi A from Rourkela, Orissa. *Indian J Med Res* 2006; 124(1): 109-11. |
| Das (2016) | Das S, Samajpati S, Ray U, Roy I, Dutta S. Antimicrobial resistance and molecular subtypes of Salmonella enterica serovar Typhi isolates from Kolkata, India over a 15 years period 1998-2012. Int J Med Microbiol 2016; 25: 25. |
| Das (2017) | Das S, Samajpati S, Roy I, et al. Molecular Subtyping of Salmonella enterica Serovar Typhi by Pulsed-Field Gel Electrophoresis and Multiple-Locus Variable-Number Tandem-Repeat Analysis in India: Their Association with Antimicrobial Resistance Profiles. *Jpn J Infect Dis* 2017; 70(5): 536-43. |
| Dashti (2008) | Dashti AA, Jadaon MM, Habeeb F, West PW, Panigrahi D, Amyes SG. Salmonella enterica Serotype Typhi in Kuwait and its reduced susceptibility to ciprofloxacin. *J Chemother* 2008; 20(3): 297-302. |
| Dhanashree (2007) | Dhanashree B. Antibiotic susceptibility profile of Salmonella enterica serovars: trend over three years showing re-emergence of chloramphenicol sensitivity and rare serovars. *Indian J Med Sci* 2007; 61(10): 576-9. |
| Dhar (1993) | Dhar KL, Thomas MS, Abraham G. Changing trends in the treatment of Salmonella Typhi infections in north-western India. *J Assoc Physicians India* 1993; 41(9): 616-7. |
| Dheer (2013) | Dheer G, Kundra S, Goel A, Singh T, Berry V. Changing spectrum of antibiotic sensitivity in enteric fever- a six year retrospective study in north india. *Res J Pharm Biol Chem Sci* 2013; 4(4): 492-8. |
| Dilruba (2006) | Dilruba A, D'Costa LT, Khorshed A, Nair GB, Hossain MA. Multidrug-resistant Salmonella enterica serovar Typhi isolates with high-level resistance to ciprofloxacin in Dhaka, Bangladesh. *Antimicrob Agents Chemother* 2006; 50(10): 3516-7. |
| Dimitrov (2005) | Dimitrov TS, Panigrahi D, Emara M, Al-Nakkas A, Awni F, Passadilla R. Incidence of bloodstream infections in a speciality hospital in Kuwait: 8-Year experience. *Med Princ Pract* 2005; 14(6): 417-21. |
| Dimitrov (2010) | Dimitrov T, Dashti AA, Albaksami O, Jadaon MM. Detection of mutations in the gyrA gene in fluoroquinolone resistance Salmonella enterica serotypes Typhi and Paratyphi A isolated from the Infectious Diseases Hospital, Kuwait. *J Clin Pathol* 2010; 63(1): 83-7. |
| Dougle (1997) | Dougle M, Hendriks E, Sanders E, Dorigo-Zetsma JW. Laboratory investigations in the diagnosis of septicaemia and malaria. *East Afr Med J* 1997; 74(6): 353-6. |
| Duggal (2014) | Duggal S, Rongpharpi SR, Gur R, Nayar R, Arora VM. Etiology and susceptibility of blood stream infections in a Referral Hospital in north Delhi: A one year study. *Res J Pharm Biol Chem Sci* 2014; 5(2): 1859-64. |
| Duggal (2016) | Duggal S, Banerjee P, Chugh TD. Review of fifty culture proven salmonella cases. *Indian J Med Sci* 2016; 68(1): 67-72. |
| Dutta (2001)ᵃ | Dutta P, Mitra U, Dutta S, De A, Chatterjee MK, Bhattacharya SK. Ceftriaxone therapy in ciprofloxacin treatment failure typhoid fever in children. *Indian J Med Res* 2001a; 113: 210-3. |
| Dutta (2001)ᵇ | Dutta P, Mitra U, Datta S, et al. Ciprofloxacin susceptible Salmonella Typhi with treatment failure. *J Trop Pediatr* 2001b; 47(4): 252-3. |
| Dutta (2005) | Dutta S, Sur D, Manna B, Bhattacharya SK, Deen JL, Clemens JD. Rollback of Salmonella enterica serotype Typhi resistance to chloramphenicol and other antimicrobials in Kolkata, India. *Antimicrob Agents Chemother* 2005; 49(4): 1662-3. |
| Dutta (2014) | Dutta S, Das S, Mitra U, et al. Antimicrobial resistance, virulence profiles and molecular subtypes of Salmonella enterica serovars Typhi and Paratyphi A blood isolates from Kolkata, India during 2009-2013. *PLoS ONE* 2014; 9(8): e101347. |
| Eibach (2016) | Eibach D, Campos CB, Krumkamp R, et al. Extended spectrum beta-lactamase producing Enterobacteriaceae causing bloodstream infections in rural Ghana, 2007-2012. *Int J Med Microbiol* 2016; 306(4): 249-54. |
| Eibacha (2016) | Eibacha D, Al-Emrana HM, Dekker DM, et al. The emergence of reduced ciprofloxacin susceptibility in Salmonella enterica causing bloodstream infections in rural Ghana. *Clin Infect Dis* 2016; 62(Suppl. 1): S32-S6. |
| El-Din (1996) | El-Din SS, Haseeb NM, Hussein MM, Abdel Wahab MF, Helmy AZ, El-Sagheer M. Chloramphenicol drug failure in typhoid fever. *J Egypt Public Health Assoc* 1996; 71(1-2): 63-78. |
| Elumalai (2014) | Elumalai S, Muthu G, Selvam RE, Ramesh S. Detection of TEM-, SHV- and CTX-M-type beta-lactamase production among clinical isolates of Salmonella species. *J Med Microbiol* 2014; 63(Pt 7): 962-7. |
| Farhana (2015) | Farhana K, Sayeed MA, Feroza Kaneez C, et al. Typhoid fever in young children in Bangladesh: clinical findings, antibiotic susceptibility pattern and immune responses. *PLoS Negl Trop Dis* 2015; 9(4). |
| Feasey (2015) | Feasey NA, Gaskell K, Wong V, et al. Rapid emergence of multidrug resistant, H58-lineage Salmonella Typhi in Blantyre, Malawi. *PLoS Negl Trop Dis* 2015; 9(4). |
| Ganesh (2010) | Ganesh R, Janakiraman L, Vasanthi T, Sathiyasekeran M. Profile of typhoid fever in children from a tertiary care hospital in Chennai-South India. *Indian J Pediatr* 2010; 77(10): 1089-92. |
| Garcia (2014) | Garcia C, Lejon V, Horna G, et al. Intermediate susceptibility to ciprofloxacin among Salmonella enterica serovar Typhi isolates in Lima, Peru. *J Clin Microbiol* 2014; 52(3): 968-70. |
| Garg (1994) | Garg K, Mangal N, Mathur HC. Clinical profile of multi drug resistant typhoid fever in Jaipur City. *Indian Pediatr* 1994; 31(2): 191-3. |
| Garg (2007) | Garg A, Anupurba S, Garg J, Goyal RK, Sen MR. Bacteriological profile and antimicrobial resistance of blood culture isolates from a university hospital. *J Indian Acad Clin Med* 2007; 8(2): 139-43. |
| Garg (2013) | Garg A, Verma S, Kanga A, Singh D, Singh B. Antimicrobial resistance pattern and in vivo activity of azithromycin in Salmonella isolates. *Indian J Med Microbiol* 2013; 31(3): 287-9. |
| Gautam (2002) | Gautam V, Gupta NK, Chaudhary U, Arora DR. Sensitivity pattern of Salmonella serotypes in Northern India. *Braz J Infect Dis* 2002; 6(6): 281-7. |
| Geetha (2014) | Geetha VK, Yugendran T, Srinivasan R, Harish BN. Plasmid-mediated quinolone resistance in typhoidal Salmonellae: a preliminary report from South India. *Indian J Med Microbiol* 2014; 32(1): 31-4. |
| Girgis (1995) | Girgis NI, Sultan Y, Hammad O, Farid Z. Comparison of the efficacy, safety and cost of cefixime, ceftriaxone and aztreonam in the treatment of multidrug-resistant Salmonella Typhi septicemia in children. *Pediatr Infect Dis J* 1995; 14(7): 603-5. |
| Gordon (2001) | Gordon MA, Walsh AL, Chaponda M, et al. Bacteraemia and mortality among adult medical admissions in Malawi - Predominance of non-Typhi Salmonellae and Streptococcus pneumoniae. *J Infect* 2001; 42(1): 44-9. |
| Gross (2011) | Gross U, Amuzu SK, Ciman Rd, et al. Bacteremia and antimicrobial drug resistance over Time, Ghana. *Emerg Infect Dis* 2011; 17(10): 1879-82. |
| Gu (2015) | Gu W, Yang Z, Chen Y, et al. Molecular characteristics of Salmonella enterica Paratyphi A in Yunnan Province, southwest China. *Infect Genet Evol* 2015; 30: 181-5. |
| Guha (2005) | Guha S, Jalan BY, Dey S, Easow JM, Wilson G, Shivananda PG. Salmonella bacteraemia in Pokhara: emergence of antibiotic resistance. *Nepal Med Coll J* 2005; 7(1): 23-5. |
| Gupta (1992) | Gupta S, Meena HS. Changing profile of enteric fever--in summer-91. *J Assoc Physicians India* 1992; 40(11): 726-9. |
| Gupta (1993) | Gupta B, Kumar R, Khurana S. Multi drug resistant Salmonella Typhi in Ludhiana (Punjab). *Indian J Pathol Microbiol* 1993; 36(1): 5-7. |
| Gupta (2009)ᵃ | Gupta V, Kaur J, Chander J. An increase in enteric fever cases due to Salmonella Paratyphi A in & around Chandigarh. *Indian J Med Res* 2009a; 129(1): 95-8. |
| Gupta (2009)ᵇ | Gupta V, Kaur J, Kaistha N. Re-emerging chloramphenicol sensitivity and emerging low level ciprofloxacin resistance among Salmonella enterica serotype typhi isolates in North India. Trop Doct 2009; 39(1): 28-30. |
| Gupta (2013) | Gupta V, Singla N, Bansal N, Kaistha N, Chander J. Trends in the antibiotic resistance patterns of enteric fever isolates - a three year report from a tertiary care centre. *Malays J Med Sci* 2013; 20(4): 71-5. |
| Gurung (2017) | Gurung B, Pandey S, Shah DK, et al. Antibiogram pattern of Salmonella in blood samples of enteric fever patients at lalitpur, nepal. *Asian Pac J Trop Dis* 2017; 7(1): 21-4. |
| Hafiz (1998) | Hafiz S, Habib F, Ahmad N, Haq I, Husain R. Typhoid fevers: treatment with lomefloxacin. *J Pak Med Assoc* 1998; 48(6): 168-70. |
| Haldar (1995) | Haldar KK, Basak S, Chakraborty AK, Das S. Transferable drug resistance in Salmonella Typhi strains isolated from an outbreak at Calcutta in the recent past. *J Indian Med Assoc* 1995; 93(8): 299-300, 15. |
| Halder (1992) | Halder KK, Saha Dalal B, Ghose E, Sanyal S. Chloramphenicol resistant Salmonella Typhi: The cause of recent outbreak of enteric fever in Calcutta. *Indian J Pathol Microbiol* 1992; 35(1): 11-7. |
| Hannan (1991) | Hannan A. Changing pattern of Salmonella typhi isolates in Pakistan and their unchanged response to ofloxacin. International Journal of Experimental and Clinical Chemotherapy 1991; 4(4): 225-9. |
| Hardjo Lugito (2017) | Hardjo Lugito NP, Cucunawangsih. Antimicrobial resistance of Salmonella enterica serovars Typhi and Paratyphi isolates from a general hospital in Karawaci, Tangerang, Indonesia: A five-year review. *Int J Microbiol* 2017; 2017: 6215136. |
| Harichandran (2017) | Harichandran D, Dinesh KR. Antimicrobial susceptibility profile, treatment outcome and serotype distribution of clinical isolates of Salmonella enterica subspecies enterica: a 2-year study from Kerala, South India. *Infect Drug Resist* 2017; 10: 97-101. |
| Harish (2006) | Harish BN, Menezes GA, Sarangapani K, Parija SC. Fluoroquinolone resistance among Salmonella enterica serovar Paratyphi A in Pondicherry [2]. *Indian J Med Res* 2006; 124: 585-7. |
| Hasan (2008) | Hasan R, Zafar A, Abbas Z, Mahraj V, Malik F, Zaidi A. Antibiotic resistance among Salmonella enterica serovars Typhi and Paratyphi A in Pakistan (2001-2006). *J Infect Dev Ctries* 2008; 2(4): 289-94. |
| Hazir (2002) | Hazir T, Qazi SA, Abbas KA, Khan MA. Therapeutic re-appraisal of multiple drug resistant Salmonella typhi (MDRST) in Pakistani children. *J Pak Med Assoc* 2002; 52(3): 123-7. |
| Hermans (1996) | Hermans PW, Saha SK, van Leeuwen WJ, Verbrugh HA, van Belkum A, Goessens WH. Molecular typing of Salmonella Typhi strains from Dhaka (Bangladesh) and development of DNA probes identifying plasmid-encoded multidrug-resistant isolates. *J Clin Microbiol* 1996; 34(6): 1373-9. |
| Hien (1994) | Hien TT, Duong NM, Ha HD, et al. A randomized comparative study of fleroxacin and ceftriaxone in enteric fever. *Trans R Soc Trop Med Hyg* 1994; 88(4): 464-5. |
| Hien (1995) | Hien TT, Bethell DB, Hoa NTT, et al. Short course of ofloxacin for treatment of multidrug-resistant typhoid. *Clin Infect Dis* 1995; 20(4): 917-23. |
| Hoa (1998) | Hoa NTT, Diep TS, Wain J, et al. Community-acquired septicaemia in southern Viet Nam: The importance of multidrug-resistant Salmonella Typhi. *Trans R Soc Trop Med Hyg* 1998; 92(5): 503-8. |
| Holt (2009) | Holt KE, Baker S, Dongol S, et al. High-throughput bacterial SNP typing identifies distinct clusters of Salmonella Typhi causing typhoid in Nepalese children. *BMC Infect Dis* 2009; 10(144). |
| Holt (2012) | Holt KE, Dutta S, Manna B, et al. High-resolution genotyping of the endemic Salmonella Typhi population during a Vi (typhoid) vaccination trial in Kolkata. *PLoS Negl Trop Dis* 2012; 6(1): e1490. |
| Hosoglu (2003) | Hosoglu S, Loeb M, Geyik MF, Ucmak H, Jayaratne P. Molecular epidemiology of invasive Salmonella Typhi in southeast Turkey. *Clin Microbiol Infect* 2003; 9(7): 727-30. |
| Huenger (2010) | Huenger F, Agyekum A, Nkrumah B, et al. Massive emergence of multidrug-resistant Enterobacteriaceae in blood culture isolates of children in Ghana. *Clin Microbiol Infect* 2010; 16: S88. |
| Ikram (2015) | Ikram S, Hussain S, Aslam A, Khan MD, Ahmed I. Evaluation of the current trends in the antimicrobial susceptibility patterns of typhoid Salmonellae. *Pak J Med Health Sci* 2015; 9(4): 1247-50. |
| Imran (2018) | Imran M, Dost S and Saleem M. Pattern of antibiotic resistance among patients with enteric fever.Indo American Journal of Pharmaceutical Sciences 2018;5(8):7797-7801 |
| Islam (1993) | Islam MN, Afroza A, Hasan Z, Majumder B, Hossain A. Recent antibiogram pattern and clinical profile of typhoid fever in children - A study of 36 cases. *Bangladesh J Child Health* 1993; 17(3): 93-6. |
| Iyer (2017) | Iyer RN, Jangam RR, Jacinth A, Venkatalakshmi A, Nahdi FB. Prevalence and trends in the antimicrobial susceptibility pattern of Salmonella enterica serovars Typhi and Paratyphi A among children in a pediatric tertiary care hospital in South India over a period of ten years: a retrospective study. *Eur J Clin Microbiol Infect Dis* 2017; 07: 07. |
| Jain (2013) | Jain S, Chugh TD. Antimicrobial resistance among blood culture isolates of Salmonella enterica in New Delhi. *J Infect Dev Ctries* 2013; 7(11): 788-95. |
| Javaid (2012) | Javaid H, Zafar A, Ahmed JM, Ejaz H, Zubair M. Changing patterns of antimicrobial susceptibility of salmonella Typhi at the children's hospital Lahore. *Pak J Med Health Sci* 2012; 6(1): 201-4. |
| Jesudason (1992) | Jesudason MV, John TJ. Plasmid mediated multidrug resistance in Salmonella Typhi. *Indian J Med Res* 1992; 95: 66-7. |
| Jog (2008) | Jog S, Soman R, Singhal T, Rodrigues C, Mehta A, Dastur FD. Enteric fever in Mumbai--clinical profile, sensitivity patterns and response to antimicrobials. *J Assoc Physicians India* 2008; 56: 237-40. |
| John (2001) | John M. Decreasing clinical response of quinolones in the treatment of enteric fever. *Indian J Med Sci* 2001; 55(4): 189-94. |
| Joshi (2004) | Joshi S, Wattal C, Sharma A, Oberoi JK, Prasad KJ. Quinolones - Drug of choice for enteric fever? *Indian J Med Microbiol* 2004; 22(4): 271-2. |
| Joshi (2007) | Joshi S, Amarnath SK. Fluoroquinolone resistance in Salmonella Typhi and S. Paratyphi A in Bangalore, India. *Trans R Soc Trop Med Hyg* 2007; 101(3): 308-10. |
| Joshi (2011) | Joshi BG, Keyal K, Pandey R, Shrestha BM. Clinical profile and sensitivity pattern of salmonella serotypes in children: A hospital based study. *Journal of Nepal Paediatric Society* 2011; 31(3): 180-3. |
| Joshi (2019) | Joshi S, Adhikary R, Beena HB, et al. Trends in antibiotic susceptibility of enteric fever isolates from South India, 2002–2013.Medical Journal Armed Forces India 2019;75(1):81-85 |
| Kabra (2000) | Kabra SK, Madhulika, Talati A, Soni N, Patel S, Modi RR. Multidrug-resistant typhoid fever. *Trop Doct* 2000; 30(4): 195-7. |
| Kadappu (2003) | Kadappu KK, Bhat R, Kurian B. Pattern of multiple drug resistance in enteric fever in Manipal, India. *Trop Doct* 2003; 33(3): 189-91. |
| Kalonji (2015) | Kalonji LM, Post A, Phoba MF, et al. Invasive Salmonella infections at multiple aurveillance aites in the Democratic Republic of the Congo, 2011-2014. *Clin Infect Dis* 2015; 61: S346-S53. |
| Kapil (1997) | Kapil A, Sood S, Reddaiah VP, Das B, Seth P. Paratyphoid fever due to Salmonella enterica serotype Paratyphi A. *Emerg Infect Dis* 1997; 3(3): 407. |
| Kapil (2002) | Kapil A, Renuka, Das B. Nalidixic acid susceptibility test to screen ciprofloxacin resistance in Salmonella Typhi. *Indian J Med Res* 2002; 115(February): 49-54. |
| Karamat (1996) | Karamat K, Butt T, Hannan A, et al. Problem of multi-drug resistant typhoid fever in Rawalpindi/Islamabad area. *Pak Armed Forces Med J* 1996; 46(2): 48-54. |
| Kariuki (2000) | Kariuki S, Gilks C, Revathi G, Hart CA. Genotypic analysis of multidrug-resistant Salmonella enterica Serovar Typhi, Kenya. *Emerg Infect Dis* 2000; 6(6): 649-51. |
| Kariuki (2004) | Kariuki S, Revathi G, Muyodi J, et al. Characterization of multidrug-resistant typhoid outbreaks in Kenya. *J Clin Microbiol* 2004; 42(4): 1477-82. |
| Kariuki (2010) | Kariuki S, Revathi G, Kiiru J, et al. Typhoid in Kenya is associated with a dominant multidrug-resistant Salmonella enterica serovar Typhi haplotype that is also widespread in Southeast Asia. *J Clin Microbiol* 2010; 48(6): 2171-6. |
| Karki (2013) | Karki AB, Bhatta DR, Shrestha B, et al. Higher nalidixic acid resistance pattern of Salmonella isolates from enteric fever patients in kathmandu model hospital Nepal. *Res J Pharm Biol Chem Sci* 2013; 4(2): 1687-93. |
| Kasper (2010) | Kasper MR, Sokhal B, Blair PJ, Wierzba TF, Putnam SD. Emergence of multidrug-resistant Salmonella enterica serovar Typhi with reduced susceptibility to fluoroquinolones in Cambodia. *Diagn Microbiol Infect Dis* 2010; 66(2): 207-9. |
| Kavita (2010) | Kavita N, Channappa ST, Gaddad SM. Antimicrobial susceptibility of Salmonella Typhi in India. *J Infect Dev Ctries* 2010; 4(2): 70-3. |
| Khan (1994) | Khan MA, Hayat Z, Sadick A. Ofloxacin in the treatment of typhoid fever resistant to chloramphenicol and amoxicillin. *Clin Ther* 1994; 16(5): 815-8. |
| Khan (2010) | Khan FY, Elshafie SS, Almaslamani M, et al. Epidemiology of bacteraemia in Hamad general hospital, Qatar: A one year hospital-based study. *Travel Med Infect Dis* 2010; 8(6): 377-87. |
| Khan (2012) | Khan MI, Soofi SB, Ochiai Leon R, et al. Epidemiology, clinical presentation, and patterns of drug resistance of Salmonella Typhi in Karachi, Pakistan. *J Infect Dev Ctries* 2012; 6(10): 704-14. |
| Khanal (2007) | Khanal B, Sharma SK, Bhattacharya SK, Bhattarai NR, Deb M, Kanungo R. Antimicrobial susceptibility patterns of Salmonella enterica serotype Typhi in eastern Nepal. *J Health Popul Nutr* 2007; 25(1): 82-7. |
| Khanal (2008) | Khanal B, Sharma SK, Amatya R, Poudyal N. Salmonella Typhi and Paratyphi A in Eastern Nepal-Trends in Antimicrobial Resistance. *Int J Infect Dis* 2008; 12: E109-E. |
| Khanal (2017) | Khanal PR, Satyal D, Bhetwal A, et al. Renaissance of conventional first-line antibiotics in Salmonella enterica clinical isolates: Assessment of MICs for therapeutic antimicrobials in enteric fever cases from Nepal. *Biomed Res Int* 2017; 2017: 2868143. |
| Khanam (2014) | Khanam F, Qadri F. Characterization of the induced immune responses, clinical presentation and antibiotic susceptibility pattern in Bangladeshi S. Paratyphi A bacteremic patients. *Int J Infect Dis* 2014; 21: 343. |
| Kharbanda (2013) | Kharbanda P, Duggal N, Mahajan RK, Chaskar P, Hans C. Changing antimicrobial susceptibility of Salmonella isolates: A retrospective study of 3 year (2008-2010) from a tertiary care hospital. *J Pure Appl Microbiol* 2013; 7(2): 1455-6. |
| Ki-Zerbo (2000) | Ki-Zerbo GA, Sawadogo AB, Kyelem N, Zoubga A, Thiombiano R, Durand G. Enterobacteriaceae bacteriemia in human deficiency virus seropositive in patients at Bobo-Dioulasso hospital (Burkina Faso): Study of 26 cases. *Med Mal Infect* 2000; 30(12): 753-6. |
| Koirala (2013) | Koirala S, Basnyat B, Arjyal A, et al. Gatifloxacin versus ofloxacin for the treatment of uncomplicated enteric fever in Nepal: an open-label, randomized, controlled trial. *PLoS Negl Trop Dis* 2013; 7(10): e2523. |
| Komolpis (1999) | Komolpis P, Srifuengfung S, Dhiraputra C, Pingwang B. Salmonella bacteremia: serotype distribution and antimicrobial susceptibility during 1991-1995. *Journal of Infectious Diseases and Antimicrobial Agents* 1999; 16(2): 49-52. |
| Koul (1991) | Koul PB, Murali MV, Sharma PP, Ghai OP, Ramchandran VG, Talwar V. Multi drug resistant Salmonella typhi infection: clinical profile and therapy. *Indian Pediatr* 1991; 28(4): 357-61. |
| Kuijpers (2015) | Kuijpers LMF, Veng CH, Sar D, et al. Ongoing outbreak of Salmonella enterica serovar Paratyphi A infections, Phnom Penh, Cambodia. *J Infect Dev Ctries* 2015; 9(4): 438-40. |
| Kuijpers (2017) | Kuijpers LMF, Phe T, Veng CH, et al. The clinical and microbiological characteristics of enteric fever in Cambodia, 2008-2015. *PLoS Negl Trop Dis* 2017; 11(9): e0005964. |
| Kumar (2001) | Kumar R, Aneja KR, Punia AK, et al. Changing pattern of biotypes, phage types & drug resistance of Salmonella Typhi in Ludhiana during 1980-1999. *Indian J Med Res* 2001; 113: 175-80. |
| Kumar (2002) | Kumar R, Aneja KR, Roy P, Sharma M, Gupta R, Ram S. Evaluation of minimum inhibitory concentration of quinolones and third generation cephalosporins to Salmonella Typhi isolates. *Indian J Med Sci* 2002; 56(1): 1-8. |
| Kumar (2007) | Kumar R, Gupta N, Shalini. Multidrug-resistant typhoid fever. *Indian J Pediatr* 2007; 74(1): 39-42. |
| Kumar (2008) | Kumar S, Rizvi M, Berry N. Rising prevalence of enteric fever due to multidrug-resistant Salmonella: an epidemiological study. *J Med Microbiol* 2008; 57(Pt 10): 1247-50. |
| Kumar (2009) | Kumar Y, Sharma A, Mani KR. High level of resistance to nalidixic acid in Salmonella enterica serovar Typhi in Central India. *J Infect Dev Ctries* 2009; 3(6): 467-9. |
| Kumar (2013)ᵃ | Kumar Y, Sharma A, Mani KR. Antibiogram profile of Salmonella enterica serovar Typhi in India - A two year study. *Trop Life Sci Res* 2013a; 24(1): 45-54. |
| Kumar (2013)ᵇ | Kumar MS, Kumar GSV, Prashanth HV, Prakash R, Veena K, Chandrashekar SC. Antimicrobial susceptibility of enteric fever Salmonellae isolated from blood culture. *Res J Pharm Biol Chem Sci* 2013b; 4(3): 318-24. |
| Labi (2014) | Labi AK, Obeng-Nkrumah N, Addison NO, Donkor ES. Salmonella blood stream infections in a tertiary care setting in Ghana. *BMC Infect Dis* 2014; 14: 3857. |
| Lakshmi (2006) | Lakshmi V, Ashok R, Susmita J, Shailaja VV. Changing trends in the antibiograms of Salmonella isolates at a tertiary care hospital in Hyderabad. *Indian J Med Microbiol* 2006; 24(1): 45-8. |
| Lefebvre (2005) | Lefebvre N, Gning SB, Nabeth P, et al. Clinical and laboratory features of typhoid fever in Senegal. A 70-case study [French]. *Med Trop* 2005; 65(6): 543-8. |
| Leung (2012) | Leung DT, Bogetz J, Itoh M, et al. Factors associated with encephalopathy in patients with Salmonella enterica serotype Typhi bacteremia presenting to a diarrheal hospital in Dhaka, Bangladesh. *Am J Trop Med Hyg* 2012; 86(4): 698-702. |
| Lewis (2005) | Lewis MD, Serichantalergs O, Pitarangsi C, et al. Typhoid fever: a massive, single-point source, multidrug-resistant outbreak in Nepal. *Clin Infect Dis* 2005; 40(4): 554-61. |
| Limpitikul (2014) | Limpitikul W, Henpraserttae N, Saksawad R, Laoprasopwattana K. Typhoid outbreak in Songkhla, Thailand 2009-2011: Clinical outcomes, susceptibility patterns, and reliability of serology tests. *PLoS ONE* 2014; 9(11). |
| Ling (2006) | Ling JH, Wang Y, Li JJ, Xu YC, Xie XL, Chen MJ. Comparison of antimicrobial susceptability of common gram-negative isolates from blood stream between Hong Kong and Beijing patients. [Chinese]. *Chinese Journal of Infection and Chemotherapy* 2006; 6(4): 251-4. |
| Liu (2011) | Liu B, Yin S, Ma S, et al. Clinical analysis of an outbreak of paratyphoid A in eastern suburb of Kunming city. [Chinese]. *Medical Journal of National Defending Forces in Southwest China* 2011; 21(3): 288-90. |
| Lugito (2017) | Lugito NPH and Cucunawangsih. Antimicrobial resistance of Salmonella enterica serovars Typhi and Paratyphi isolates from a general hospital in Karawaci, Tangerang, Indonesia: a five-year review.International Journal of Microbiology 2017;6215136(30): |
| Lunguya (2012) | Lunguya O, Lejon V, Phoba MF, et al. Salmonella Typhi in the Democratic Republic of the Congo: Fluoroquinolone decreased susceptibility on the rise. *PLoS Negl Trop Dis* 2012; 6(11): e1921. |
| Ly (1998) | Ly N, Chinh N, Parry C, Diep T, Wain J, White N. Randomised trial of azithromycin versus ofloxacin for the treatment of typhoid fever in adult. *Med J Indones* 1998; 7: 202-6. |
| Madan (1991) | Madan A, Dhar A, Kulshrestha PP, Laghate VD, Dhar P. Preliminary observation on drug resistant cases of typhoid fever. *J Assoc Physicians India* 1991; 39(6): 449-51. |
| Madhulika (2004) | Madhulika U, Harish BN, Parija SC. Current pattern in antimicrobial susceptibility of Salmonella Typhi isolates in Pondicherry. *Indian J Med Res* 2004; 120(2): 111-4. |
| Mahapatra (2016) | Mahapatra A, Patro S, Choudhury S, Padhee A, Das R. Emerging enteric fever due to switching biotype of Salmonella (Paratyphi A) in Eastern Odisha. *Indian J Pathol Microbiol* 2016; 59(3): 327-9. |
| Mahende (2015) | Mahende C, Ngasala B, Lusingu J, et al. Bloodstream bacterial infection among outpatient children with acute febrile illness in north-eastern Tanzania. *BMC Res Notes* 2015; 8: 289. |
| Maheshwari (1996) | Maheshwari VD, Agarwal SK. Present status of drug resistance in cases of enteric fever in Rajasthan. *J Assoc Physicians India* 1996; 44(9): 618-9. |
| Mahmud (2014) | Mahmud ASM, Hakim MA. Prevalence and antimicrobial resistance of Salmonella strains isolated from blood cultures in Dhaka Division, Bangladesh. *J Pure Appl Microbiol* 2014; 8(3): 2315-23. |
| Makkar (2018) | Makkar A, Gupta S, Khan ID, et al. Epidemiological Profile and Antimicrobial Resistance Pattern of Enteric Fever in a Tertiary Care Hospital of North India - a Seven Year Ambispective Study.Acta Medica (Hradec Kralove) 2018;61(4):125-130 |
| Malla (2005) | Malla S, Kansakar P, Serichantalergs, Rahman M, Basnet S. Epidemiology of typhoid and paratyphoid fever in Kathmandu: two years study and trends of antimicrobial resistance. *JNMA J Nepal Med Assoc* 2005; 44(157): 18-22. |
| Maltha (2014) | Maltha J, Guiraud I, Kabore B, et al. Frequency of severe malaria and invasive bacterial infections among children admitted to a rural hospital in Burkina Faso. *PLoS ONE* 2014; 9(2). |
| Mamun (2004) | Mamun KZ, Tabassum S, Ashna SM, Hart CA. Molecular analysis of multi-drug resistant Salmonella Typhi from urban paediatric population of Bangladesh. *Bangladesh Med Res Counc Bull* 2004; 30(3): 81-6. |
| Manchanda (2006) | Manchanda V, Bhalla P, Sethi M, Sharma VK. Treatment of enteric fever in children on the basis of current trends of antimicrobial susceptibility of Salmonella enterica serovar Typhi and Paratyphi A. *Indian J Med Microbiol* 2006; 24(2): 101-6. |
| Mandal (2002) | Mandal S, Mandal MD, Pal NK. Antimicrobial resistance pattern of Salmonella typhi isolates in Kolkata, India during 1991-2001: a retrospective study. *Jpn J Infect Dis* 2002; 55(2): 58-9. |
| Mandal (2003) | Mandal S, Mandal MD, Pal NK. Ofloxacin minimum inhibitory concentration versus disk diffusion zone diameter for Salmonella enterica serovar Typhi isolates: problems in the detection of ofloxacin resistance. *Jpn J Infect Dis* 2003; 56(5-6): 210-2. |
| Mandal (2004) | Mandal S, Mandal MD, Pal NK. Reduced minimum inhibitory concentration of chloramphenicol for Salmonella enterica serovar Typhi. *Indian J Med Sci* 2004; 58(1): 16-23. |
| Mandal (2006) | Mandal S, Mandal M, Pal N. Antibiotic resistance of Salmonella enterica serovar Paratyphi A in India: Emerging and reemerging problem. *J Postgrad Med* 2006; 52(3): 163-6. |
| Mandal (2009) | Mandal S, Mandal MD, Pal NK. In vitro activity of gentamicin and amikacin against Salmonella enterica serovar Typhi: a search for a treatment regimen for typhoid fever. *East Mediterr Health J* 2009; 15(2): 264-8. |
| Mandal (2012) | Mandal S, Debmandal M, Pal NK. Antibiotic resistance of Salmonella enterica serovar Typhi in Kolkata, India, and in vitro experiments on effect of combined chemotherapy. *ScientificWorldJournal* 2012; 2012(454059). |
| Mandeep (2006) | Mandeep W, Rajni G, Premila P, Rajesh M, Pushpa A, Mani K. Age-related clinical and microbiological characteristics of enteric fever in India. *Trans R Soc Trop Med Hyg* 2006; 100(10): 942-8. |
| Marks (2010) | Marks F, Adu-Sarkodie Y, Hunger F, et al. Typhoid fever among children, ghana. *Emerg Infect Dis* 2010; 16(11): 1796-7. |
| Marks (2017) | Marks F, Kalckreuth V, Aaby P, et al. Incidence of invasive Salmonella disease in sub-Saharan Africa: A multicentre population-based surveillance study. *Lancet Glob Health* 2017; 5(3): e310-e23. |
| Maskey (2006) | Maskey AP, Day JN, Phung QT, et al. Salmonella enterica serovar Paratyphi A and S. enterica serovar Typhi cause indistinguishable clinical syndromes in Kathmandu, Nepal. *Clin Infect Dis* 2006; 42(9): 1247-53. |
| Maskey (2008) | Maskey AP, Basnyat B, Thwaites GE, Campbell JI, Farrar JJ, Zimmerman MD. Emerging trends in enteric fever in Nepal: 9124 cases confirmed by blood culture 1993-2003. *Trans R Soc Trop Med Hyg* 2008; 102(1): 91-5. |
| Mathew (2014) | Mathew R, Jobin SR. Current trends of antimicrobial susceptibility of Salmonella enterica serovar Typhi and Paratyphi a from blood cultures in a tertiary care center of Chennai, Tamil Nadu, India. *Res J Pharm Biol Chem Sci* 2014; 5(2): 1167-74. |
| Mathur (1996) | Mathur YC, Mathur NC, Lal HM. Clinical efficacy of Cefuroxime axetil in S. Typhi. *Indian Pediatr* 1996; 33(12): 1033-7. |
| Mathura (2005) | Mathura KC, Chaudhary D, Simkhada R, Pradhan M, Shrestha P, Gurubacharya DL. Study of clinical profile and antibiotic sensitivity pattern in culture positive typhoid fever cases. *Kathmandu Univ Med J (KUMJ)* 2005; 3(4): 376-9. |
| Mayxay (2013) | Mayxay M, Castonguay-Vanier J, Chansamouth V, et al. Causes of non-malarial fever in Laos: a prospective study. *Lancet Glob Health* 2013; 1(1): e46-54. |
| Mehta (1992) | Mehta A, Rodriques C, Joshi VR. Multiresistant Salmonella organisms in India. *JAMA* 1992; 267(12): 1614. |
| Mehta (2002) | Mehta M, Joshi RM. Antibiogram pattern of Salmonella species causing bacteraemia/septicaemia in a teaching hospital. *J Infect* 2002; 45(2): 133. |
| Menezes (2012) | Menezes GA, Harish BN, Khan MA, Goessens WHF, Hays JP. Antimicrobial resistance trends in blood culture positive Salmonella Typhi isolates from Pondicherry, India, 2005-2009. Clin Microbiol Infect 2012; 18(3): 239-45. |
| Menezes (2016) | Menezes GA, Harish BN, Khan MA, Goessens W, Hays JP. Antimicrobial resistance trends in blood culture positive Salmonella Paratyphi A isolates from Pondicherry, India. *Indian J Med Microbiol* 2016; 34(2): 222-7. |
| Mengo (2010) | Mengo DM, Kariuki S, Muigai A, Revathi G. Trends in Salmonella enteric serovar Typhi in Nairobi, Kenya from 2004 to 2006. *J Infect Dev Ctries* 2010; 4(6): 393-6. |
| Mirza (1995) | Mirza SH, Beeching NJ, Hart CA. The prevalence and clinical features of multi-drug resistant Salmonella Typhi infections in Baluchistan, Pakistan. *Ann Trop Med Parasitol* 1995; 89(5): 515-9. |
| Mirza (2008) | Mirza SH, Khan MA. Low-level quinolone-resistance in multi-drug resistant typhoid. *J Coll Physicians Surg Pak* 2008; 18(1): 13-6. |
| Mishra (1992) | Mishra S, Patwari AK, Anand VK, et al. Multidrug resistant typhoid fever: therapeutic considerations. *Indian Pediatr* 1992; 29(4): 443-8. |
| Mishra (1996) | Mishra OP, Gupta BL, Nath G, Prakash J. Treatment of multidrug-resistant typhoid fever. *J Trop Pediatr* 1996; 42(5): 310-1. |
| Misra (2005) | Misra RN, Bawa KS, Magu SK, Bhandari S, Nagendra A, Menon PK. Outbreak of multi-drug resistant Salmonella Typhi enteric fever in Mumbai garrison. *Med J Armed Forces India* 2005; 61(2): 148-50. |
| Misra (2016) | Misra R, Thakare R, Amrin N, Prasad KN, Chopra S, Dhole TN. Antimicrobial susceptibility pattern and sequence analysis of DNA gyrase and DNA topoisomerase IV in Salmonella enterica serovars Typhi and Paratyphi A isolates with decreased susceptibility to ciprofloxacin. *Trans R Soc Trop Med Hyg* 2016; 110(8): 472-9. |
| Moehario (2009) | Moehario LH, Tjoa E, Kiranasari A, Ningsih I, Rosana Y, Karuniawati A. Trends in antimicrobial susceptibility of gram-negative bacteria isolated from blood in Jakarta from 2002 to 2008. *J Infect Dev Ctries* 2009; 3(11): 843-8. |
| Mohanty (2006) | Mohanty S, Renuka K, Sood S, Das BK, Kapil A. Antibiogram pattern and seasonality of Salmonella serotypes in a North Indian tertiary care hospital. *Epidemiol Infect* 2006; 134(5): 961-6. |
| Muhammad (2013) | Muhammad R, Ali Z, Mehmood K, Ziauddin, Afridi AR, Bari F. Antibiotic sensitivity pattern of salmonella serotypes in patients with enteric fever in a teaching hospital. *Journal of Postgraduate Medical Institute* 2013; 27(4): 397-402. |
| Murdoch (1998) | Murdoch DA, Banatvala NA, Bone A, Shoismatulloev BI, Ward LR, Threlfall EJ. Epidemic ciprofloxacin-resistant Salmonella Typhi in Tajikistan. *Lancet* 1998; 351(9099): 339. |
| Mushtaq (2006) | Mushtaq MA. What after ciprofloxacin and ceftriaxone in treatment of Salmonella Typhi. *Pak J Med Sci* 2006; 22(1): 51-4. |
| Mutai (2018) | Mutai WC, Muigai AWT, Waiyaki P and Kariuki S. Multi-drug resistant Salmonella enterica serovar Typhi isolates with reduced susceptibility to ciprofloxacin in Kenya.BMC Microbiology 2018;18(1):187 |
| Muthumbi (2015) | Muthumbi E, Morpeth SC, Ooko M, et al. Invasive Salmonellosis in Kilifi, Kenya. *Clin Infect Dis* 2015; 61(Suppl. 4): S290-S301. |
| Muyembe-Tamfum (2009) | Muyembe-Tamfum JJ, Veyi J, Kaswa M, Lunguya O, Verhaegen J, Boelaert M. An outbreak of peritonitis caused by multidrug-resistant Salmonella Typhi in Kinshasa, Democratic Republic of Congo. *Travel Med Infect Dis* 2009; 7(1): 40-3. |
| Nakachi (2003) | Nakachi S, Nakamura T, Agha N, et al. Clinical features and early diagnosis of typhoid fever emphasizing usefulness of detecting mesenteric lymphadenopathy with ultrasound as diagnostic method. *Southeast Asian J Trop Med Public Health* 2003; 34(Suppl 2): 153-7. |
| Narain (2015) | Narain U, Gupta R. Emergence of resistance in community-acquired enteric fever. *Indian Pediatr* 2015; 52(8): 709-. |
| Narasanna (2018) | Narasanna R, Chavadi M and Chandrakanth K. Prevalence of multidrug-resistant Salmonella typhi in typhoid patients and detection of blaCTX-M2 and blaCTX-M9 genes in cefetoxime-mediated extended spectrum beta-lactamase-producing Salmonella typhi isolates.Biomedical Research (India) 2018;29(14):3015-3021 |
| Neopane (2008) | Neopane A, Singh SB, Bhatta R, Dhital B, Karki DB. Changing spectrum of antibiotic sensitivity in enteric fever. *Kathmandu Univ Med J (KUMJ)* 2008; 6(1): 12-5. |
| Nga (2012) | Nga TVT, Parry CM, Le T, et al. The decline of typhoid and the rise of non-typhoid Salmonellae and fungal infections in a changing HIV landscape: Bloodstream infection trends over 15 years in southern Vietnam. *Trans R Soc Trop Med Hyg* 2012; 106(1): 26-34. |
| Ngoun (2012) | Ngoun C, Emary K, Khun PA, et al. Enteric fever in Cambodian children is dominated by multidrug resistant H58 Salmonella enterica serovar Typhi with decreased susceptibility to ciprofloxacin. *Int J Infect Dis* 2012; 16: e427. |
| Obaro (2011) | Obaro S, Lawson L, Essen U, et al. Community acquired bacteremia in young children from central Nigeria - a pilot study. *BMC Infect Dis* 2011; 11(137). |
| Ochiai (2008) | Ochiai RL, Acosta CJ, Danovaro-Holliday MC, et al. A study of typhoid fever in five Asian countries: Disease burden and implications for controls. *Bull World Health Organ* 2008; 86(4): 260-8. |
| Okanda (2018) | Okanda T, Haque A, Ehara T, et al. Characteristics of resistance mechanisms and molecular epidemiology of fluoroquinolone-nonsusceptible salmonella enterica serovar typhi and paratyphi a isolates from a tertiary hospital in Dhaka, Bangladesh.Microbial Drug Resistance 2018;24(10):1460-1465 |
| Olsen (2004) | Olsen SJ, Pruckler J, Bibb W, et al. Evaluation of rapid diagnostic tests for typhoid fever. *J Clin Microbiol* 2004; 42(5): 1885-9. |
| Pais (1991) | Pais N, Ravindranath S, Kesavamurthy. Drug resistant typhoid in Bangalore. *J Assoc Physicians India* 1991; 39(8): 654. |
| Pal (1991) | Pal N, Ayyagari A, Panigrahi D. Outbreak of multidrug resistant S. Typhi infection. *Bull Postgrad Inst Med Educ Res Chandigarh* 1991; 25(4): 186-90. |
| Pallab (2006) | Pallab R, Jyoti S, Marak RSK, Garg RK. Predictive efficacy of nalidixic acid resistance as a marker of fluoroquinolone resistance in Salmonella enterica var Typhi. *Indian J Med Res* 2006; 124(1): 105-8. |
| Pandit (2012) | Pandit V, Kumar A, Kulkarni MM, Pattanshetty SM, Samarasinghe C, Kamath S. Study of clinical profile and antibiotic sensitivity in paratyphoid fever cases admitted at teaching hospital in South India. *J Family Med Prim Care* 2012; 1(2): 118-21. |
| Panhotra (2004) | Panhotra BR, Saxena AK, Al-Ghamdi AM. Typhoid fever due to multiresistant Salmonella enterica serovar Typhi having reduced susceptibility to ciprofloxacin and nalidixic acid resistance. *Saudi Med J* 2004; 25(10): 1509-11. |
| Parry (1998) | Parry C, Wain J, Chinh NT, Vinh H, Farrar JJ. Quinolone-resistant Salmonella Typhi in Vietnam. *Lancet* 1998; 351(9111): 1289. |
| Pathak (2012) | Pathak A, Marothi Y, Kekre V, Mahadik K, Macaden R, Lundborg CS. High prevalence of extended-spectrum beta-lactamase-producing pathogens: results of a surveillance study in two hospitals in Ujjain, India. *Infect Drug Resist* 2012; 5: 65-73. |
| Petersiel (2018) | Petersiel N, Shresta S, Tamrakar R, et al. The epidemiology of typhoid fever in the Dhulikhel area, Nepal: A prospective cohort study.PLoS ONE 2018;13 (9) (no pagination)(e0204479): |
| Phetsouvanh (2006) | Phetsouvanh R, Phongmany S, Soukaloun D, et al. Causes of community-acquired bacteremia and patterns of antimicrobial resistance in Vientiane, Laos. *Am J Trop Med Hyg* 2006; 75(5): 978-85. |
| Phoba (2012) | Phoba MF, Lunguya O, Mayimon DV, et al. Multidrug-resistant Salmonella enterica, Democratic Republic of the Congo. *Emerg Infect Dis* 2012; 18(10): 1693-4. |
| Phoba (2014) | Phoba MF, De Boeck H, Ifeka BB, et al. Epidemic increase in Salmonella bloodstream infection in children, Bwamanda, the Democratic Republic of Congo. *Eur J Clin Microbiol Infect Dis* 2014; 33(1): 79-87. |
| Phuong (1999) | Phuong CXT, Kneen R, Anh NT, Luat TD, White NJ, Parry CM. A comparative study of ofloxacin and cefixime for treatment of typhoid fever in children. *Pediatr Infect Dis J* 1999; 18(3): 245-8. |
| Pokharel (2009) | Pokharel P, Rai SK, Karki G, Katuwal A, Vitrakoti R, Shrestha SK. Study of enteric fever and antibiogram of Salmonella isolates at a teaching hospital in Kathmandu Valley. *Nepal Med Coll J* 2009; 11(3): 176-8. |
| Prabhakar (1996) | Prabhakar H, Kaur H, Lal M. Prevalence of multi-drug resistant Salmonella Typhi in Ludhiana Punjab. *Indian J Med Sci* 1996; 50(8): 277-9. |
| Pradhan (2012) | Pradhan R, Shrestha U, Gautam SC, et al. Bloodstream infection among children presenting to a general hospital outpatient clinic in urban Nepal. *PLoS ONE* 2012; 7(10): e47531. |
| Prajapati (2008) | Prajapati B, Rai GK, Rai SK, et al. Prevalence of Salmonella Typhi and Paratyphi infection in children: a hospital based study. *Nepal Med Coll J* 2008; 10(4): 238-41. |
| Punjabi (2012) | Punjabi NH, Taylor WR, Murphy GS, et al. Etiology of acute, non-malaria, febrile illnesses in Jayapura, northeastern Papua, Indonesia. *Am J Trop Med Hyg* 2012; 86(1): 46-51. |
| Punjabi (2013) | Punjabi NH, Agtini MD, Ochiai RL, et al. Enteric fever burden in North Jakarta, Indonesia: a prospective, community-based study. *J Infect Dev Ctries* 2013; 7(11): 781-7. |
| Purighalla (2017) | Purighalla S, Esakimuthu S, Reddy M, et al. Investigation into a community outbreak of Salmonella Typhi in Bengaluru, India.Indian Journal of Medical Research 2017;146(July Supplement):15-22 |
| Qaiser (2011) | Qaiser S, Irfan S, Khan E, Ahsan T, Zafar A. In vitro susceptibility of typhoidal Salmonellae against newer antimicrobial agents: a search for alternate treatment options. *J Pak Med Assoc* 2011; 61(5): 462-5. |
| Qamar (2014) | Qamar FN, Asma A, Kazi AM, Erum K, Zaidi AKM. A three-year review of antimicrobial resistance of Salmonella enterica serovars Typhi and Paratyphi A in Pakistan. *J Infect Dev Ctries* 2014; 8(8): 981-6. |
| Qamar (2018) | Qamar FN, Yousafzai MT, Sultana S, et al. A Retrospective Study of Laboratory-Based Enteric Fever Surveillance, Pakistan, 2012-2014.Journal of Infectious Diseases 2018;218(suppl_4):S201-S205 |
| Qureshi (2001) | Qureshi AH, Mushahid N, Ijaz A, et al. Changing drug susceptibility pattern of Salmonellae Paratyphi A. *J Coll Physicians Surg Pak* 2001; 11(7): 449-51. |
| Rahman (2002) | Rahman M, Ahmad A, Shoma S. Decline in epidemic of multidrug resistant Salmonella Typhi is not associated with increased incidence of antibiotic-susceptible strain in Bangladesh. *Epidemiol Infect* 2002; 129(1): 29-34. |
| Rahman (2005) | Rahman MM, Haq JA, Morshed MA, Rahman MA. Salmonella enterica serovar Typhi with decreased susceptibility to ciprofloxacin--an emerging problem in Bangladesh. *Int J Antimicrob Agents* 2005; 25(4): 345-6. |
| Rahman (2006) | Rahman M, Siddique AK, Shoma S, et al. Emergence of multidrug-resistant Salmonella enterica serotype Typhi with decreased ciprofloxacin susceptibility in Bangladesh. *Epidemiol Infect* 2006; 134(2): 433-8. |
| Rahman (2014) | Rahman BA, Wasfy MO, Maksoud MA, Hanna N, Dueger E, House B. Multi-drug resistance and reduced susceptibility to ciprofloxacin among Salmonella enterica serovar Typhi isolates from the Middle East and Central Asia. *New microbes new infect* 2014; 2(4): 88-92. |
| Rai (2012)ᵃ | Rai GK, Karki S, Prajapati B. Is antimicrobial resistance pattern of enteric fever changing in Kathmandu valley? *Journal of Nepal Paediatric Society* 2012a; 32(3): 221-8. |
| Rai (2012)ᵇ | Rai S, Jain S, Prasad KN, Ghoshal U, Dhole TN. Rationale of azithromycin prescribing practices for enteric fever in India. *Indian J Med Microbiol* 2012b; 30(1): 30-3. |
| Rajashri (2017) | Rajashri P and Amar P. Study of antibiotic sensitivity pattern of Salmonella Typhi in tertiary care centre.International Journal of Healthcare & Biomedical Research 2017;6(1):75-80 |
| Ramesh (2016) | Ramesh U, Das S, Balasubramanian A. Re-emergence of chloramphenicol-susceptible Salmonella Typhi and Paratyphi A strains in India. *Indian J Med Microbiol* 2016; 34(2): 262-3. |
| Ranju (1998) | Ranju C, Pais P, Ravindran GD, Singh G. Changing pattern of antibiotic sensitivity of Salmonella Typhi. *Natl Med J India* 1998; 11(6): 266-7. |
| Rao (1993) | Rao PS, Rajashekar V, Varghese GK, Shivananda PG. Emergence of multidrug-resistant Salmonella Typhi in rural southern India. *Am J Trop Med Hyg* 1993; 48(1): 108-11. |
| Rasaily (1994) | Rasaily R, Dutta P, Saha MR, Mitra U, Lahiri M, Pal SC. Multi-drug resistant typhoid fever in hospitalised children. Clinical, bacteriological and epidemiological profiles. *Eur J Epidemiol* 1994; 10(1): 41-6. |
| Rathish (1994) | Rathish KC, Chandrashekar MR, Nagesha CN. Multidrug resistant Salmonella Typhi in Bangalore, south India. *Indian J Med Sci* 1994; 48(4): 85-8. |
| Rathish (1995) | Rathish KC, Chandrashekar MR, Nagesha CN. An outbreak of multidrug resistant typhoid fever in Bangalore. *Indian J Pediatr* 1995; 62(4): 445-8. |
| Rathore (1996) | Rathore MH, Bux D, Hasan M. Multidrug-resistant Salmonella Typhi in Pakistani children: clinical features and treatment. *South Med J* 1996; 89(2): 235-7. |
| Raveendran (2008) | Raveendran R, Wattal C, Sharma A, Oberoi J, Prasad K, Datta S. High level ciprofloxacin resistance in Salmonella enterica isolated from blood. *Indian J Med Microbiol* 2008; 26(1): 50-3. |
| Raza (2012) | Raza S, Tamrakar R, Bhatt CP, Joshi SK. Antimicrobial susceptibility patterns of Salmonella Typhi and Salmonella Paratyphi A in a tertiary care hospital. *J Nepal Health Res Counc* 2012; 10(22): 214-7. |
| Riyaz (2015) | Riyaz C, Jayavardhana A. Current pattern of Salmonella Typhi antimicrobial susceptibility in the era of antibiotic abuse. *Indian J Basic Appl Med Res* 2015; 5(1): 400-4. |
| Rodrigues (1992) | Rodrigues C, Mehta A, Mehtar S, et al. Chloramphenicol resistance in Salmonella Typhi. Report from Bombay. *J Assoc Physicians India* 1992; 40(11): 729-32. |
| Rodrigues (2003) | Rodrigues C, Shenai S, Mehta A. Enteric fever in Mumbai, India: the good news and the bad news. *Clin Infect Dis* 2003; 36(4): 535. |
| Rupali (2004) | Rupali P, Abraham OC, Jesudason MV, et al. Treatment failure in typhoid fever with ciprofloxacin susceptible Salmonella enterica serotype Typhi. *Diagn Microbiol Infect Dis* 2004; 49(1): 1-3. |
| Sabharwal (2010) | Sabharwal ER. Ceftriaxone resistance in Salmonella Typhi - myth or a reality! *Indian J Pathol Microbiol* 2010; 53(2): 389. |
| Sabherwal (1992) | Sabherwal U, Chaudhary U, Saini S. Multidrug-resistant Salmonella Typhi in Haryana in 1989-90. *Indian J Med Res* 1992; 95: 12-3. |
| Saha (1992) | Saha MR, Dutta P, Bhattacharya SK, et al. Occurrence of multi-drug resistant Salmonella Typhi in Calcutta. *Indian J Med Res* 1992; 95(JULY): 179-80. |
| Saha (1994) | Saha SK. Antibiotic resistance of Salmonella Typhi in Bangladesh. *J Antimicrob Chemother* 1994; 33(1): 190-1. |
| Saha (1997) | Saha SK, Saha S, Ruhulamin M, Hanif M, Islam M. Decreasing trend of multiresistant Salmonella Typhi in Bangladesh. *J Antimicrob Chemother* 1997; 39(4): 554-6. |
| Saha (2001) | Saha SK, Darmstadt GL, Baqui AH, et al. Rapid identification and antibiotic susceptibility testing of Salmonella enterica serovar Typhi isolated from blood: implications for therapy. *J Clin Microbiol* 2001; 39(10): 3583-5. |
| Saha (2002) | Saha MR, Dutta P, Niyogi SK, et al. Decreasing trend in the occurrence of Salmonella enterica serotype Typhi amongst hospitalised children in Kolkata, India during 1990-2000. *Indian J Med Res* 2002; 115(FEB.): 46-8. |
| Saha (2018) | Saha S, Saha S, Das RC, et al. Enteric Fever and Related Contextual Factors in Bangladesh.American Journal of Tropical Medicine & Hygiene 2018;99(3_Suppl):20-25 |
| Saharan (2008) | Saharan G, Gupta A, Gupta BK, Sharma BP, Kochar DK, Purohit VP. Evaluation of antibiotic sensitivity pattern in cases of enteric fever in north west Rajasthan. *J Indian Med Assoc* 2008; 106(8): 528-30, 32. |
| Sania (2016) | Sania KM, Shyamasakhi PD, Krishna Pramodini KD, Sulochana KD. Evaluation of minimum inhibitory concentration of chloramphenicol for Salmonella spp. isolated from enteric fever cases in a tertiary hospital in Imphal. *Int J Pharm Sci Res* 2016; 7(9): 3815-9. |
| Saqib (2000) | Saqib A, Ahmed A. Culture and sensitivity of Salmonella species: analysis of a two year data. *J Pak Med Assoc* 2000; 50(8): 282-4. |
| Secmeer (1995) | Secmeer G, Kanra G, Cemeroglu AP, Ozen H, Ceyhan M, Ecevit Z. Salmonella Typhi infections. A 10-year retrospective study. *Turk J Pediatr* 1995; 37(4): 339-41. |
| Sekar (2003) | Sekar U, Srikanth P, Kindo AJ, Babu VP, Ramasubramanian V. Increase in minimum inhibitory concentration to quinolones and ceftriaxone in salmonellae causing enteric fever. *J Commun Dis* 2003; 35(3): 162-9. |
| Sen (1991) | Sen S, Goyal RS, Dev R. Ciprofloxacin in the management of multiple drug resistant typhoid fever. *Indian Pediatr* 1991; 28(4): 417-9. |
| Sethuraman (1994) | Sethuraman S, Mahamood M, Kareem S. Furazolidone in multi-resistant childhood typhoid fever. *Ann Trop Paediatr* 1994; 14(4): 321-4. |
| Setiabudi (1998) | Setiabudi D, Azhali MS, Garna H, Chairulfatah A. Antibiotic resistance patterns of pediatric typhoid fever at the Department of Child Health, Hasan Sadikin General Hospital, Bandung. *Med J Indones* 1998; 7: 289. |
| Seydi (2005) | Seydi M, Soumare M, Sow AI, Diop BM, Sow PS. Current aspects of Salmonella bacteremia cases in the Ibrahima Diop Mar infectious diseases clinic, Fann national hospital center (Senegal). *Med Mal Infect* 2005; 35(1): 23-7. |
| Shanahan (1998) | Shanahan PM, Jesudason MV, Thomson CJ, Amyes SG. Molecular analysis of and identification of antibiotic resistance genes in clinical isolates of Salmonella typhi from India. J Clin Microbiol 1998; 36(6): 1595-600. |
| Sharma (1993) | Sharma A, Gathwala G. Clinical profile and outcome in enteric fever. *Indian Pediatr* 1993; 30(1): 47-50. |
| Sharma (2006) | Sharma NP, Peacock SJ, Phumratanaprapin W, Day N, White N, Pukrittayakamee S. A hospital-based study of bloodstream infections in febrile patients in Dhulikhel Hospital Kathmandu University Teaching Hospital, Nepal. *Southeast Asian J Trop Med Public Health* 2006; 37(2): 351-6. |
| Sharma (2015) | Sharma, Sharma R, Gupta S. Bacteriological analysis of blood culture isolates with their antibiogram from a tertiary care hospital. *Int J Pharm Sci Res* 2015; 6(11): 4847-51. |
| Sharma (2016) | Sharma P, Dahiya S, Balaji V, et al. Typhoidal Salmonellae: Use of multi-locus sequence typing to determine population structure. *PLoS ONE* 2016; 11(9). |
| Sharma (2018) | Sharma P, Dahiya S, Manral N, et al. Changing trends of culture-positive typhoid fever and antimicrobial susceptibility in a tertiary care North Indian Hospital over the last decade.Indian Journal of Medical Microbiology 2018;36(1):70-76 |
| Sharvani (2016) | Sharvani R, Hemavathi, Dayanand DK, Shenoy P, Sarmah P. Antibiogram of Salmonella isolates: time to consider antibiotic salvage. *J Clin Diagn Res* 2016; 10(5): DC6-DC8. |
| Shenoy (2014) | Shenoy B, Prasad K, Selvi A, Joshi S, Adhikary R, Medimond. Changing trends in antibiograms and clinical profile of Salmonella enterica in pediatric population. *8th World Congress of the World Society for Pediatric Infectious Diseases (Wspid)* 2014: 105-11. |
| Shetty (2012) | Shetty AK, Shetty IN, Furtado ZV, Antony B, Boloor R. Antibiogram of Salmonella isolates from blood with an emphasis on nalidixic acid and chloramphenicol susceptibility in a tertiary care hospital in coastal karnataka: a prospective study. *J Lab Physicians* 2012; 4(2): 74-7. |
| Shirakawa (2006) | Shirakawa T, Acharya B, Kinoshita S, Kumagai S, Gotoh A, Kawabata M. Decreased susceptibility to fluoroquinolones and gyrA gene mutation in the Salmonella enterica serovar Typhi and Paratyphi A isolated in Katmandu, Nepal, in 2003. *Diagn Microbiol Infect Dis* 2006; 54(4): 299-303. |
| Shrestha (2016) | Shrestha KL, Pant ND, Bhandari R, Khatri S, Shrestha B, Lekhak B. Re-emergence of the susceptibility of the Salmonella spp. isolated from blood samples to conventional first line antibiotics. *Antimicrob Resist Infect Control* 2016; 5. |
| Shukun (2010) | Shukun W, Congjia C, Yunbo Y, Biao K, Baowei D. Nalidixic acid resistance and clonal expansion of Salmonella enterica serotype Paratyphi A in Yuxi city, China. *J Med Med Sci* 2010; 17: 320-6. |
| Shwe (2002) | Shwe TN, Nyein MM, Yi W, Mon A. Blood culture isolates from children admitted to Medical Unit III, Yangon Children's Hospital, 1998. *Southeast Asian J Trop Med Public Health* 2002; 33(4): 764-71. |
| Siddiqui (2006) | Siddiqui FJ, Rabbani F, Hasan R, Nizami SQ, Bhutta ZA. Typhoid fever in children: some epidemiological considerations from Karachi, Pakistan. *Int J Infect Dis* 2006; 10(3): 215-22. |
| Singh (1993) | Singh CP, Singh N, Brar GK, Lal G, Kumar H. Efficacy of ciprofloxacin and norfloxacin in multidrug resistant enteric fever in adults. *J Indian Med Assoc* 1993; 91(6): 156-7. |
| Singh (2011) | Singh U, Neopane A, Thapa M, Aryal N, Agrawal K. Salmonella Typhi infections and effect of fluroquinolones and third generation cephalosporins in clinical outcome. *Journal of Nepal Paediatric Society* 2011; 31(3): 216-21. |
| Singhal (2014) | Singhal L, Gupta PK, Kale P, Gautam V, Ray P. Trends in antimicrobial susceptibility of Salmonella Typhi from North India (2001-2012). *Indian J Med Microbiol* 2014; 32(2): 149-52. |
| Singla (2013) | Singla N, Bansal N, Gupta V, Chander J. Outbreak of Salmonella Typhi enteric fever in sub-urban area of North India: a public health perspective. *Asian Pac J Trop Med* 2013; 6(2): 167-8. |
| Sinha (1999) | Sinha A, Sazawal S, Kumar R, et al. Typhoid fever in children aged less than 5 years. *Lancet* 1999; 354(9180): 734-7. |
| Sood (1999) | Sood S, Kapil A, Das B, Jain Y, Kabra SK. Re-emergence of chloramphenicol-sensitive Salmonella Typhi. *Lancet* 1999; 353(9160): 1241-2. |
| Sridhar (1995) | Sridhar CB, Kulkarni RD. Reassessment of frequency of occurrence of typhoid fever and cost efficacy analysis of antibiotic therapy. *J Assoc Physicians India* 1995; 43(10): 679-84. |
| Srikantiah (2006) | Srikantiah P, Girgis FY, Luby SP, et al. Population-based surveillance of typhoid fever in Egypt. *Am J Trop Med Hyg* 2006; 74(1): 114-9. |
| Srikantiah (2007) | Srikantiah P, Vafokulov S, Luby SP, et al. Epidemiology and risk factors for endemic typhoid fever in Uzbekistan. *Trop Med Int Health* 2007; 12(7): 838-47. |
| Srirangaraj (2014) | Srirangaraj S, Kali A, Charles MV. A study of antibiogram of Salmonella enterica serovar Typhi isolates from Pondicherry, India. *Australas Med J* 2014; 7(4): 185-90. |
| Stoesser (2013) | Stoesser N, Moore CE, Pocock JM, et al. Pediatric bloodstream infections in Cambodia, 2007 to 2011. *Pediatr Infect Dis J* 2013; 32(7): e272-e6. |
| Sucindar (2017) | Sucindar M and Kumaran SS. Profile of culture positive enteric fever in children admitted in a tertiary care hospital.Journal of Evolution of Medical and Dental Sciences-Jemds 2017;6(88):6112-6117 |
| Sur (2009) | Sur D, Ochiai RL, Bhattacharya SK, et al. A cluster-randomized effectiveness trial of Vi typhoid vaccine in India. *N Engl J Med* 2009; 361(4): 335-44. |
| Sur (2018) | Sur D, Barkume C, Mukhopadhyay B, et al. A Retrospective Review of Hospital-Based Data on Enteric Fever in India, 2014-2015.Journal of Infectious Diseases 2018;218(suppl_4):S206-S213 |
| Suruchi (2014) | Suruchi B, Anil K, Ganju SA, Atal S. Antibiotic susceptibility pattern of Salmonella enterica serovar Typhi and Paratyphi a from North India: the changing scenario. *Int J Pharma Bio Sci* 2014; 5(4). |
| Takkar (1994) | Takkar VP, Kumar R, Khurana S, Takkar R. Comparison of ciprofloxacin versus cephelexin and gentamicin in the treatment of multi-drug resistant typhoid fever. *Indian Pediatr* 1994; 31(2): 200-1. |
| Takkar (1995) | Takkar VP, Kumar R, Takkar R, Khurana S. Resurgence of chloramphenicol sensitive Salmonella Typhi. *Indian Pediatr* 1995; 32(5): 586-7. |
| Tamang (2007) | Tamang MD, Oh J, Seol S, et al. Emergence of multidrug-resistant Salmonella enterica serovar Typhi associated with a class 1 integron carrying the dfrA7 gene cassette in Nepal. *Int J Antimicrob Agents* 2007; 30(4): 330-5. |
| Tankhiwale (2003)ᵃ | Tankhiwale SS, Agrawal G, Jalgaonkar SV. A preliminary report on current antibiogram of Salmonella enterica serotype typhi in Nagpur. *Indian J Med Microbiol* 2003a; 21(4): 292. |
| Tankhiwale (2003)ᵇ | Tankhiwale SS, Agrawal G, Jalgaonkar SV. An unusually high occurrence of Salmonella enterica serotype Paratyphi A in patients with enteric fever. *Indian J Med Res* 2003b; 117: 10-2. |
| Tanmoy (2018) | Tanmoy AM, Westeel E, Bruyne KD, et al. Salmonella enterica serovar typhi in Bangladesh: exploration of genomic diversity and antimicrobial resistance.mBio 2018;9(6):02112-18 |
| Threlfall (1992) | Threlfall EJ, Ward LR, Rowe B, et al. Widespread occurrence of multiple drug-resistant Salmonella Typhi in India. *Eur J Clin Microbiol Infect Dis* 1992; 11(11): 990-3. |
| Thriemer (2012) | Thriemer K, Ley B, Ame S, et al. The burden of invasive bacterial infections in Pemba, Zanzibar. *PLoS ONE* 2012; 7(2). |
| Trivedi (2010) | Trivedi ND, Trivedi UN. Case report and pathological result based study on typhoid patients. *Int J Pharm Sci Rev Res* 2010; 2(2): 22-3. |
| Vala (2016) | Vala S, Shah U, Ahmad SA, Scolnik D, Glatstein M. Resistance patterns of typhoid fever in children: A longitudinal community-based study. *Am J Ther* 2016; 23(5): E1151-E4. |
| Vandenberg (2010) | Vandenberg O, Nyarukweba DZ, Ndeba PM, et al. Microbiologic and clinical features of Salmonella species isolated from bacteremic children in eastern Democratic Republic of Congo. *Pediatr Infect Dis J* 2010; 29(6): 504-10. |
| Veeraraghavan (2016) | Veeraraghavan B, Anandan S, Muthuirulandi Sethuvel DP, Puratchiveeran N, Walia K, Devanga Ragupathi NK. Molecular characterization of intermediate susceptible typhoidal Salmonella to ciprofloxacin, and its impact. *Mol Diagn Ther* 2016; 20(3): 213-9. |
| Venkatesh (2013) | Venkatesh BM, Joshi S, Adhikary R, Bhaskar BH. Antibiogram of Salmonella Typhi and Salmonella Paratyphi A in a tertiary care hospital in 2012. *Indian J Pathol Microbiol* 2013; 56(4): 484-5. |
| Verma (1996) | Verma M, Chhatwal J, Saini V, Singh T. Enteric fever below 2 years of age. *Indian Pediatr* 1996; 33(3): 229-30. |
| Vidyalakshmi (2008) | Vidyalakshmi K, Yashavanth R, Chakrapani M, et al. Epidemiological shift, seasonal variation and antimicrobial susceptibility patterns among enteric fever pathogens in South India. *Trop Doct* 2008; 38(2): 89-91. |
| Vinh (2005) | Vinh H, Duong NM, Phuong le T, et al. Comparative trial of short-course ofloxacin for uncomplicated typhoid fever in Vietnamese children. *Ann Trop Paediatr* 2005; 25(1): 17-22. |
| Vlieghe (2012) | Vlieghe ER, Phe T, De Smet B, et al. Azithromycin and ciprofloxacin resistance in Salmonella bloodstream infections in Cambodian adults. *PLoS Negl Trop Dis* 2012; 6(12): e1933. |
| Vlieghe (2013) | Vlieghe E, Phe T, De Smet B, et al. Increase in Salmonella enterica serovar Paratyphi A infections in Phnom Penh, Cambodia, January 2011 to August 2013. *Euro Surveill* 2013; 18(39). |
| Vollaard (2004) | Vollaard AM, Ali S, van Asten HA, et al. Risk factors for typhoid and paratyphoid fever in Jakarta, Indonesia. *JAMA* 2004; 291(21): 2607-15. |
| Wain (1998) | Wain J, Diep TS, Ho VA, et al. Quantitation of bacteria in blood of typhoid fever patients and relationship between counts and clinical features, transmissibility, and antibiotic resistance. *J Clin Microbiol* 1998; 36(6): 1683-7. |
| Wain (2001) | Wain J, Bay PVB, Vinh H, et al. Quantitation of bacteria in bone marrow from patients with typhoid fever: Relationship between counts and clinical features. *J Clin Microbiol* 2001; 39(4): 1571-6. |
| Wang (2014) | Wang M, Kan B, Yang J, et al. Epidemiological characteristics of typhoid fever and antibiotic susceptibility testing of Salmonella Typhi isolates in Guangxi, 1994-2013 [Chinese]. *Chung Hua Liu Hsing Ping Hsueh Tsa Chih* 2014; 35(8): 930-4. |
| Wang (2018) | Wang H, Yu H, Zheng W, et al. Genomic epidemiology of Salmonella paratyphi A strains isolated in Hangzhou area. [Chinese].Chinese Journal of Microbiology and Immunology (China) 2018;38(2):116-123 |
| Wankhede (2018) | Wankhede SV and Apurva. Prevalence and antibiogram of Salmonella typhi and paratyphi A isolates from a tertiary care hospital.Indian Journal of Basic and Applied Medical Research 2018;7(Diagnostic):31-38 |
| Wasfy (2002) | Wasfy MO, Frenck R, Ismail TF, Mansour H, Malone JL, Mahoney FJ. Trends of multiple-drug resistance among Salmonella serotype Typhi isolates during a 14-year period in Egypt. *Clin Infect Dis* 2002; 35(10): 1265-8. |
| Woods (2006) | Woods CW, Murdoch DR, Zimmerman MD, et al. Emergence of Salmonella enterica serotype Paratyphi A as a major cause of enteric fever in Kathmandu, Nepal. *Trans R Soc Trop Med Hyg* 2006; 100(11): 1063-7. |
| Wu (2010) | Wu W, Wang H, Lu J, et al. Genetic diversity of Salmonella enteric serovar Typhi and Paratyphi in Shenzhen, China from 2002 through 2007. *BMC Microbiol* 2010; 10: 32. |
| Yadav (2016) | Yadav VC, Kiran VR, Sharma R. Enteric fever in Bastar tribal region-prevalence and sensitivity patterns. *J Evol Med Dent Sci* 2016; 5(53): 3526-30. |
| Yanagi (2009) | Yanagi D, de Vries GC, Rahardjo D, et al. Emergence of fluoroquinolone-resistant strains of Salmonella enterica in Surabaya, Indonesia. *Diagn Microbiol Infect Dis* 2009; 64(4): 422-6. |
| Yashavanth (2010) | Yashavanth R, Vidyalakshmi K. The Re-Emergence of chloramphenicol sensitivity among enteric fever pathogens in Mangalore. *J Clin Diagn Res* 2010; 4(5): 3016-108. |
| Yu (2010) | Yu R, Liang J, Xu H. Clinical analysis of 125 children with typhoid fever from 1993 to 2008 in Chongqing area. [Chinese]. *Chinese Journal of Practical Pediatrics* 2010; 25(7): 539-42. |
| Yu (2011) | Yu F, Fan S, Fan X, et al. Analysis of characteristics of paratyphoid A in 157 Chinese inpatients between 1998 and 2009. *Eur J Clin Microbiol Infect Dis* 2011; 30(1): 71-5. |
| Yu (2017) | Yu AT, Amin N, Rahman MW and Luby S. Typhoid fever case fatality rate in patients presenting to a laboratory network in Dhaka, Bangladesh.American Journal of Tropical Medicine and Hygiene 2017;97 (5 Supplement 1):352 |
| Zehra (2017) | Zehra NM, Irfan F, Mirza IA, et al. Current Trends of Antimicrobial Susceptibility of Typhoidal Salmonellae Isolated at Tertiary Care Hospital.Jcpsp, Journal of the College of Physicians & Surgeons - Pakistan 2017;27(11):690-692 |
| Zellweger (2017) | Zellweger RM, Basnyat B, Shrestha P, et al. A 23-year retrospective investigation of Salmonella Typhi and Salmonella Paratyphi isolated in a tertiary Kathmandu hospital.PLoS Neglected Tropical Diseases [electronic resource] 2017;11(11):e0006051 |
| Zhao (2016) | Zhao JY, Zhang SY, Mu YJ, et al. Drug tolerance and PFGE molecular typing of Salmonella Paratyphi A isolated in Dengfeng, Henan province, 2009-2015 [Chinese]. *Chung Hua Liu Hsing Ping Hsueh Tsa Chih* 2016; 37(5): 714-7. |
| Zhuang (2012) | Zhuang L, Zhang YJ, Tang Z, et al. Epidemiologic characteristics of typhoid and paratyphoid fever on related drug resistance and molecular types regarding Salmonella Typhi and S. Paratyphi, in Jiangsu province [Chinese]. *Chung Hua Liu Hsing Ping Hsueh Tsa Chih* 2012; 33(12): 1269-72. |

**Figures:**

**Figure S1. Meta-analysis and median prevalence comparison.** A scatter graph to compare the median prevalence and pooled prevalence estimates of resistance from a random effects meta-analysis, for every subgroup in the study. Linear regression line fit to the data with an intercept of -1.49 and a coefficient of 1.04. High correlation was observed with an r^2^ of 0.97 and p<0.001.


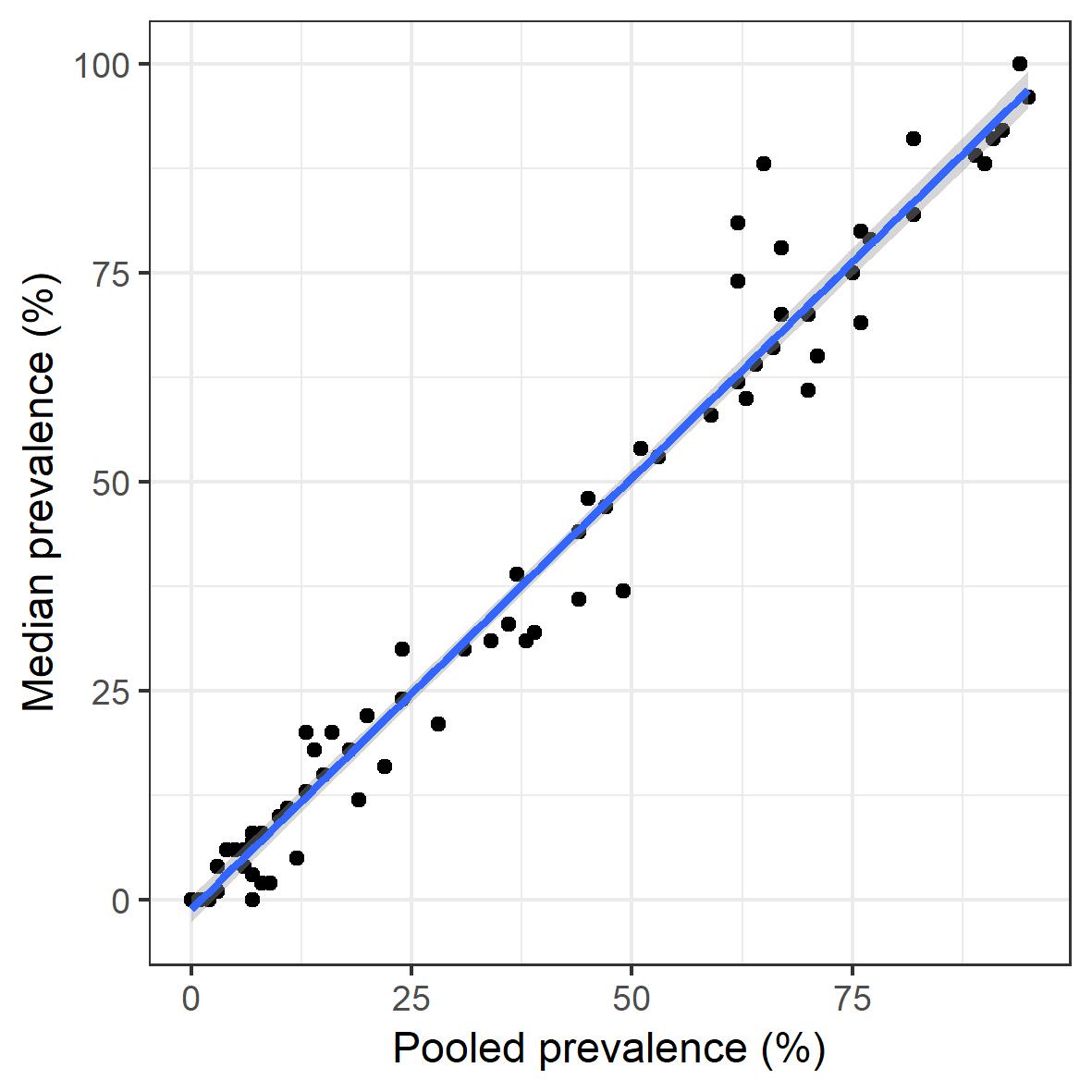


**Figure S2a. Sensitivity analysis:** Random effects metaanalysis results for studies on MDR *S.* Typhi in South Asia, comparing results for all studies (red) with only those reporting AST methods used (olive); (c) internal control strain reported (green), and those on over 50 isolates of *S.* Typhi (blue), resistance breakpoints clearly stated (pink), grouped by 5-year period.

**
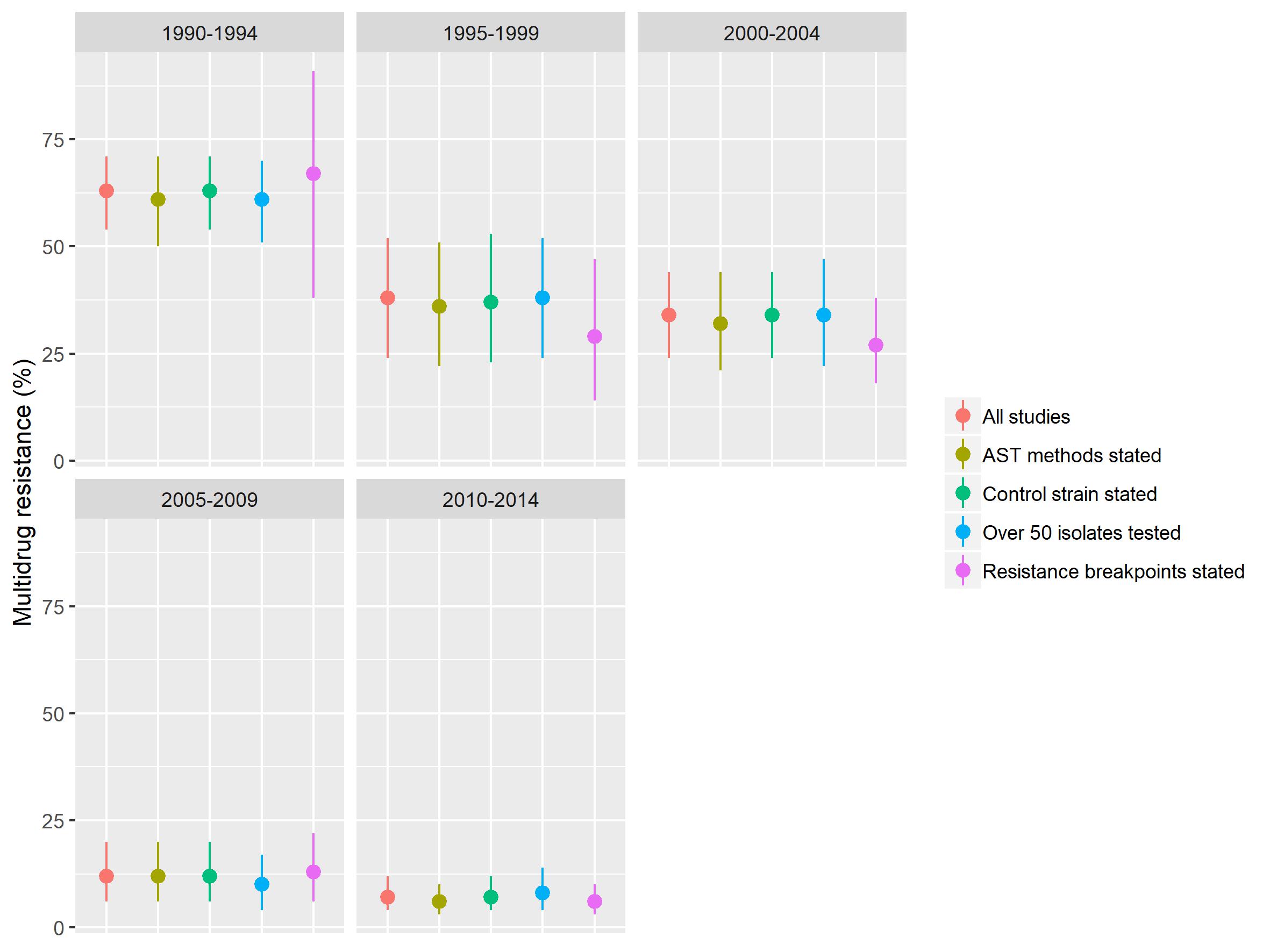
**

**Figure S2b. Sensitivity analysis:** Random effects metaanalysis results for studies on FQNS *S.* Typhi in South Asia, comparing results for all studies (red) with only those reporting AST methods used (olive); (c) internal control strain reported (green), and those on over 50 isolates of *S.* Typhi (blue), resistance breakpoints clearly stated (pink), grouped by 5-year period.

**
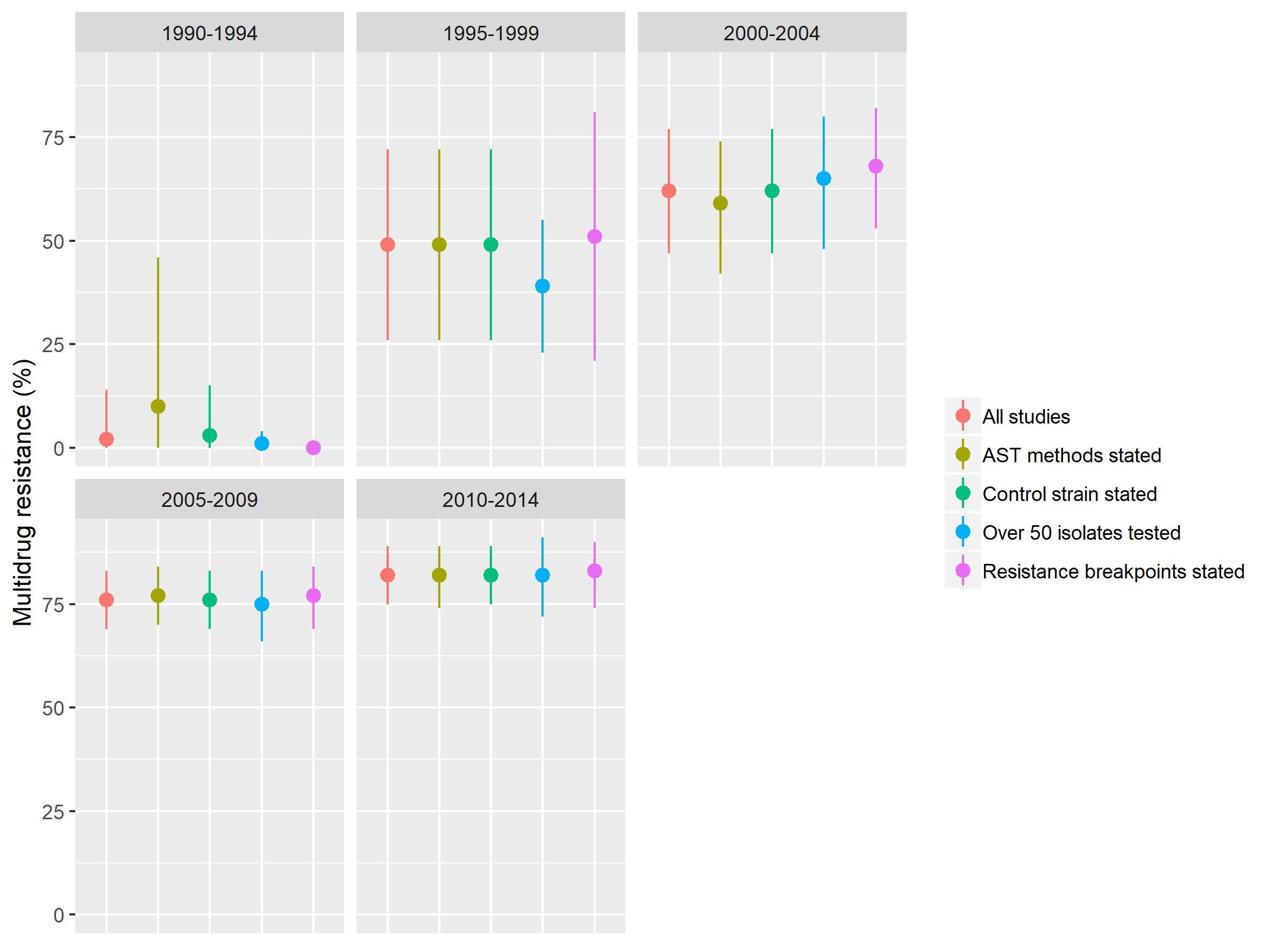
**

**Figure S3.** **MDR *S.* Typhi in sub-Saharan Africa:** Forest plots illustrating the prevalence of MDR amongst *S.* Typhi isolates in sub-Saharan Africa, grouped by five-year time-periods. Individual study results are displayed with 95% confidence intervals, the pooled prevalence [95%CI] for each subgroup is represented by the blue diamond: (a) Central sSA; (b) Eastern sSA; (c) Western sSA. Multidrug resistance is defined as concurrent resistance against ampicillin, chloramphenicol and co-trimoxazole. **
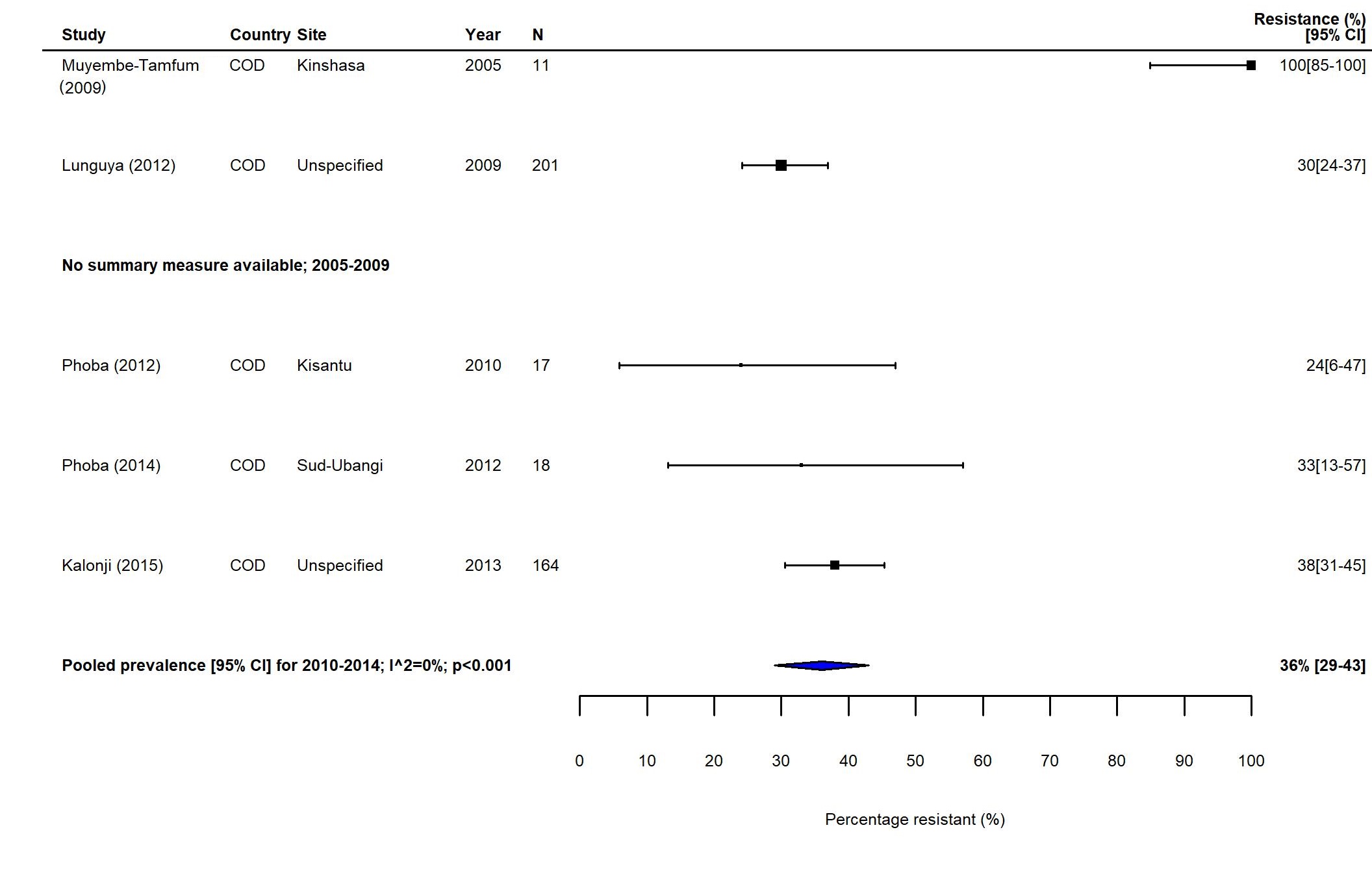
**

(a)


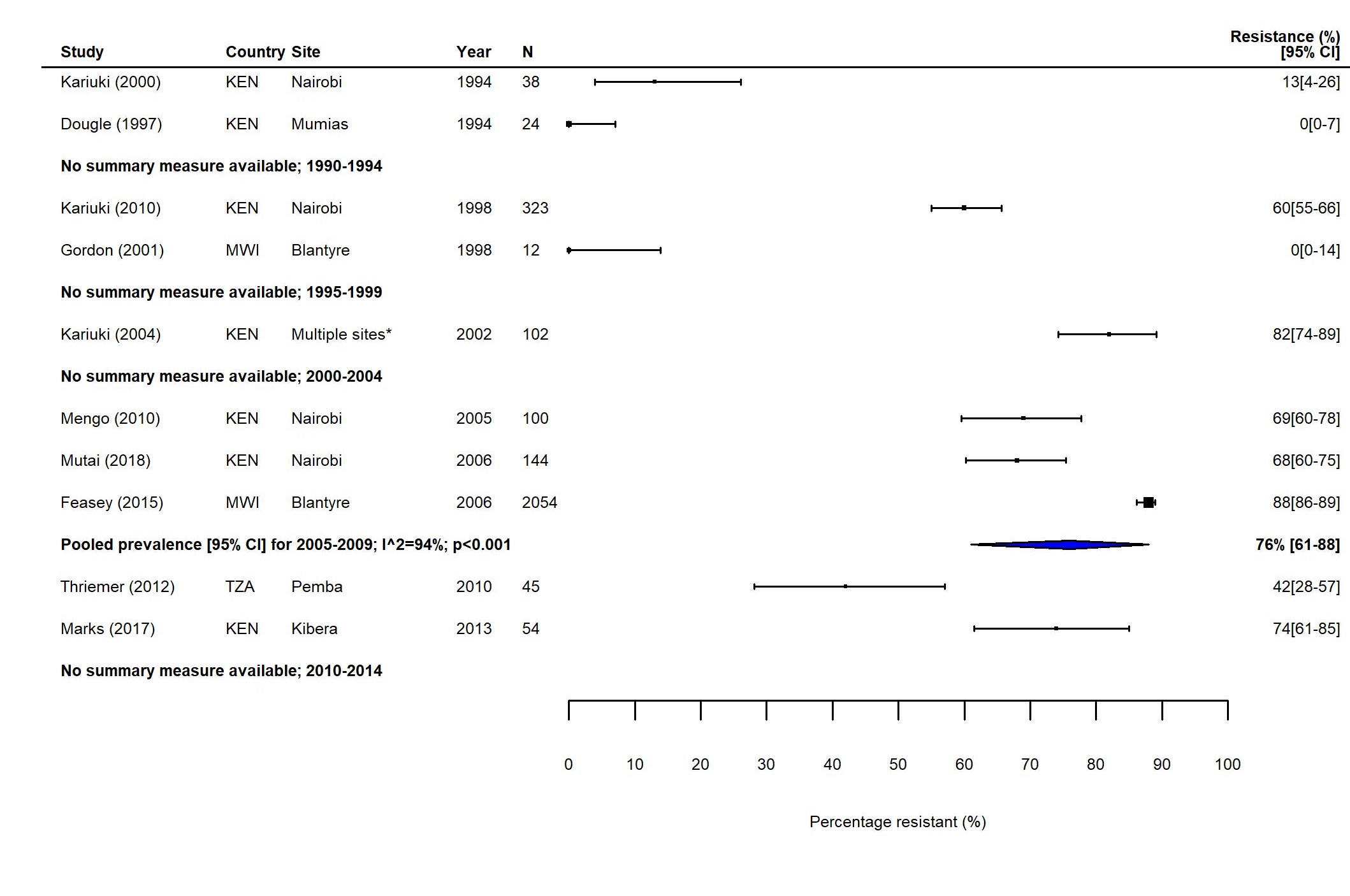


(b)


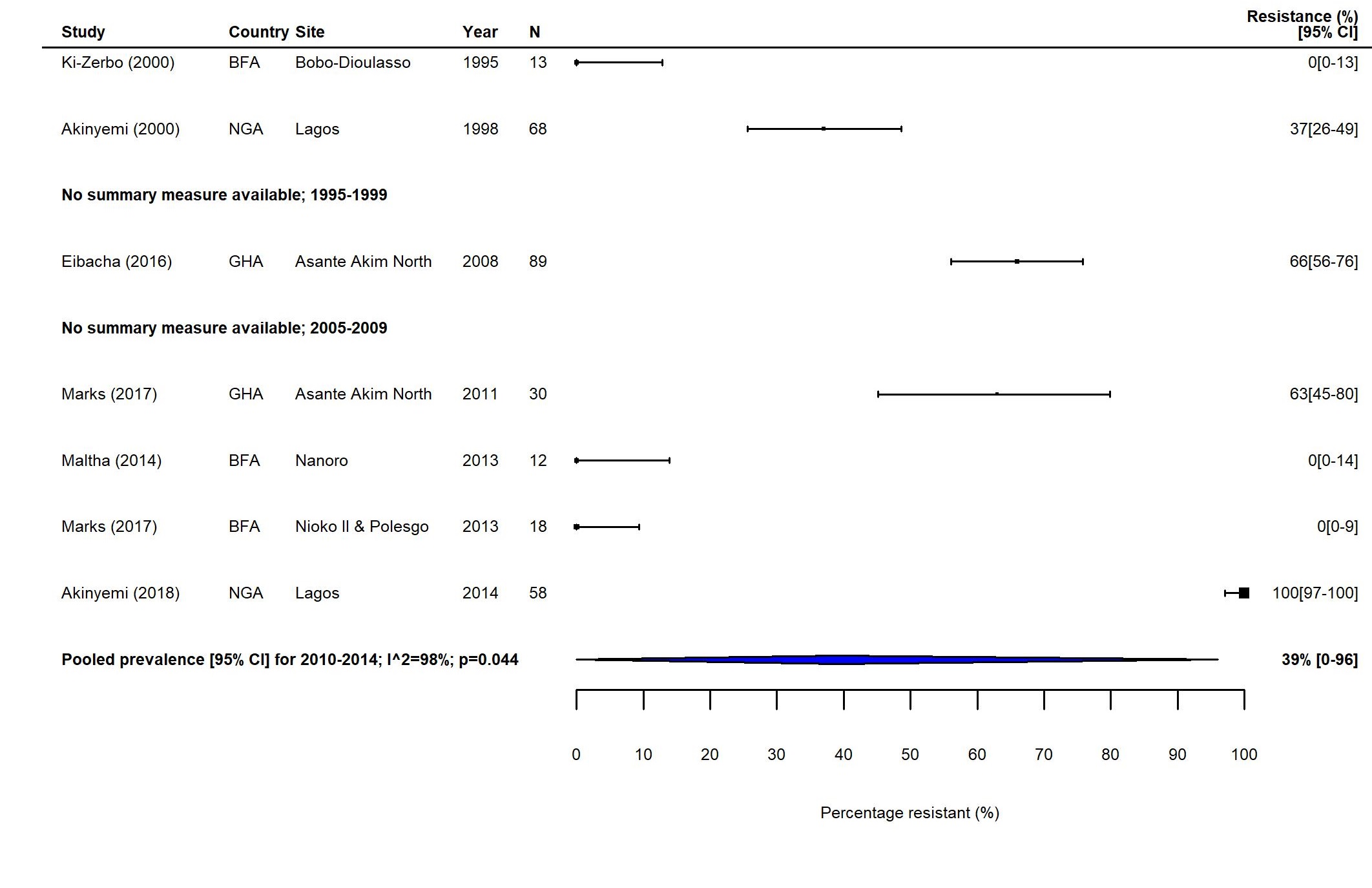


(c)

**Figure S4. MDR *S.* Typhi in North Africa and the Middle East (NAME):** Forest plots illustrating the prevalence of MDR amongst *S.* Typhi isolates in NAME, grouped by five-year time-periods. Individual study results are displayed with 95% confidence intervals, the pooled prevalence [95%CI] for each subgroup is represented by the blue diamond. Multidrug resistance is defined as concurrent resistance against ampicillin, chloramphenicol and co-trimoxazole.

**
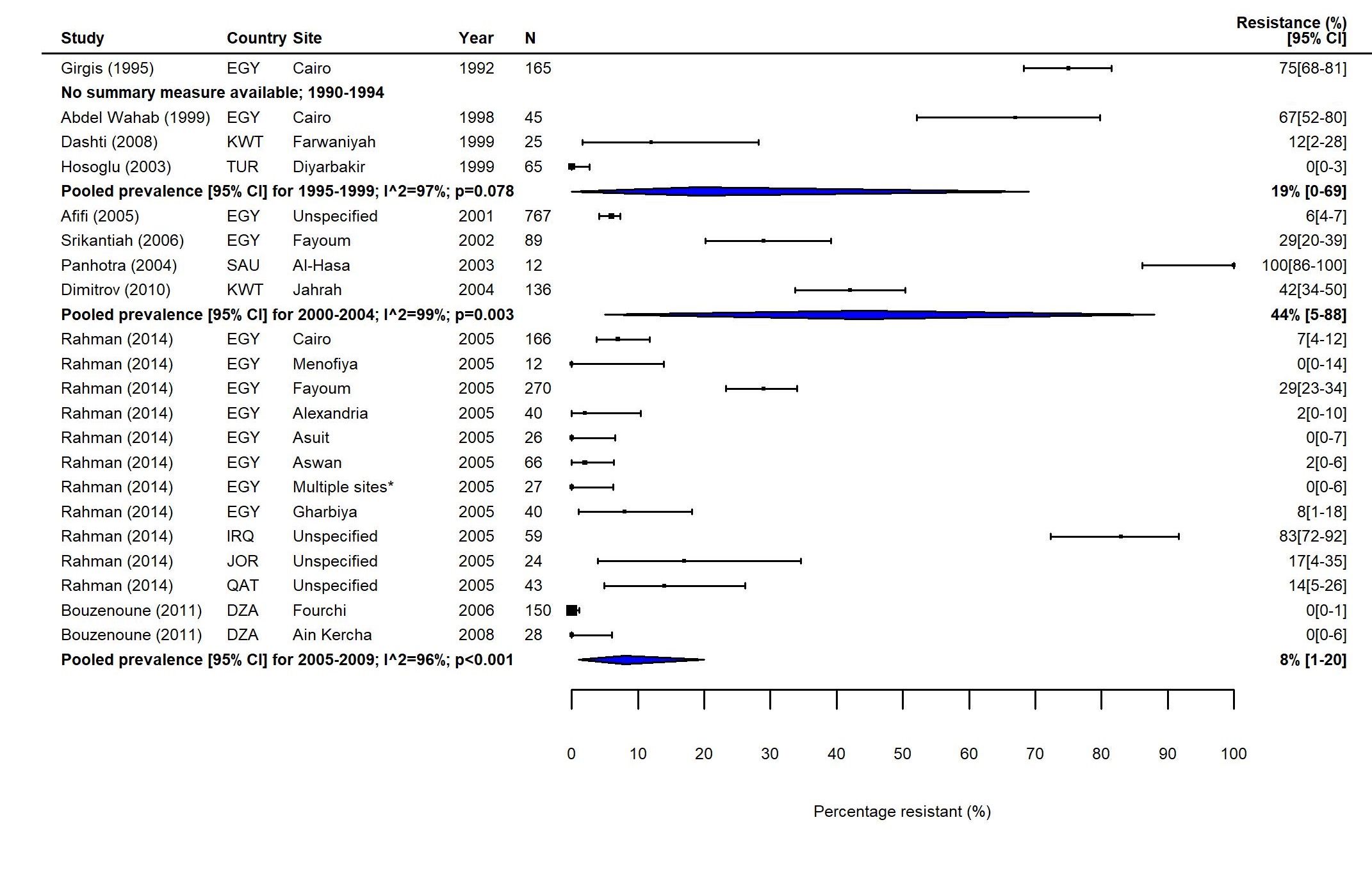
**

**Figure S5. MDR *S.* Typhi in East Asia:** Forest plots illustrating the prevalence of MDR amongst *S.* Typhi isolates in East Asia, grouped by five-year time-periods. Individual study results are displayed with 95% confidence intervals, the pooled prevalence [95%CI] for each subgroup is represented by the blue diamond. Multidrug resistance is defined as concurrent resistance against ampicillin, chloramphenicol and co-trimoxazole. **
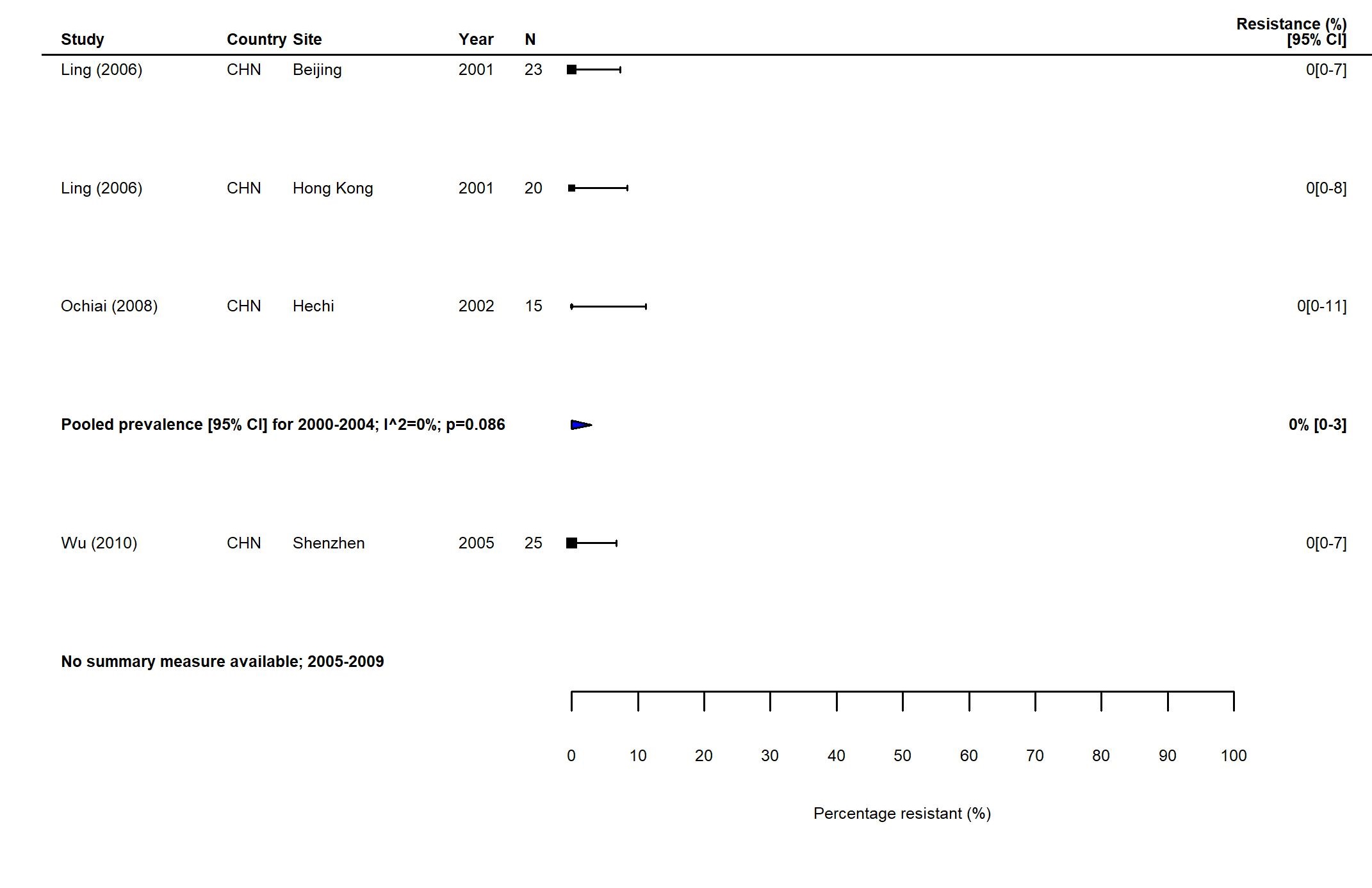
**

**Figure S6.** **FQNS *S.* Typhi in sub-Saharan Africa:** Forest plots illustrating the prevalence of FQNS amongst *S.* Typhi in sub-Saharan Africa, grouped by five-year time-periods. Individual study results are displayed with 95% confidence intervals, the pooled prevalence [95%CI] for each subgroup are represented by the blue diamonds: (a) Central sSA; (b) Eastern sSA; (c) Western sSA.

To allow the analysis of resistance trends over time despite typhoid-specific breakpoint changes for ciprofloxacin (CLSI, 2012) coming into effect during our study (1990-2018), we categorised intermediate (ciprofloxacin MIC 0.12–0.5 μg/ml) and resistant isolates (≥ 1 μg/ml) according to the updated breakpoints, as well as isolates with 'decreased ciprofloxacin (or fluoroquinolone) susceptibility' (ciprofloxacin MIC 0.125–1.0 μg/ml) and nalidixic acid resistant isolates (as proxy marker for 'decreased ciprofloxacin (or fluoroquinolone) susceptibility') as fluoroquinolone non-susceptible (FQNS).

**
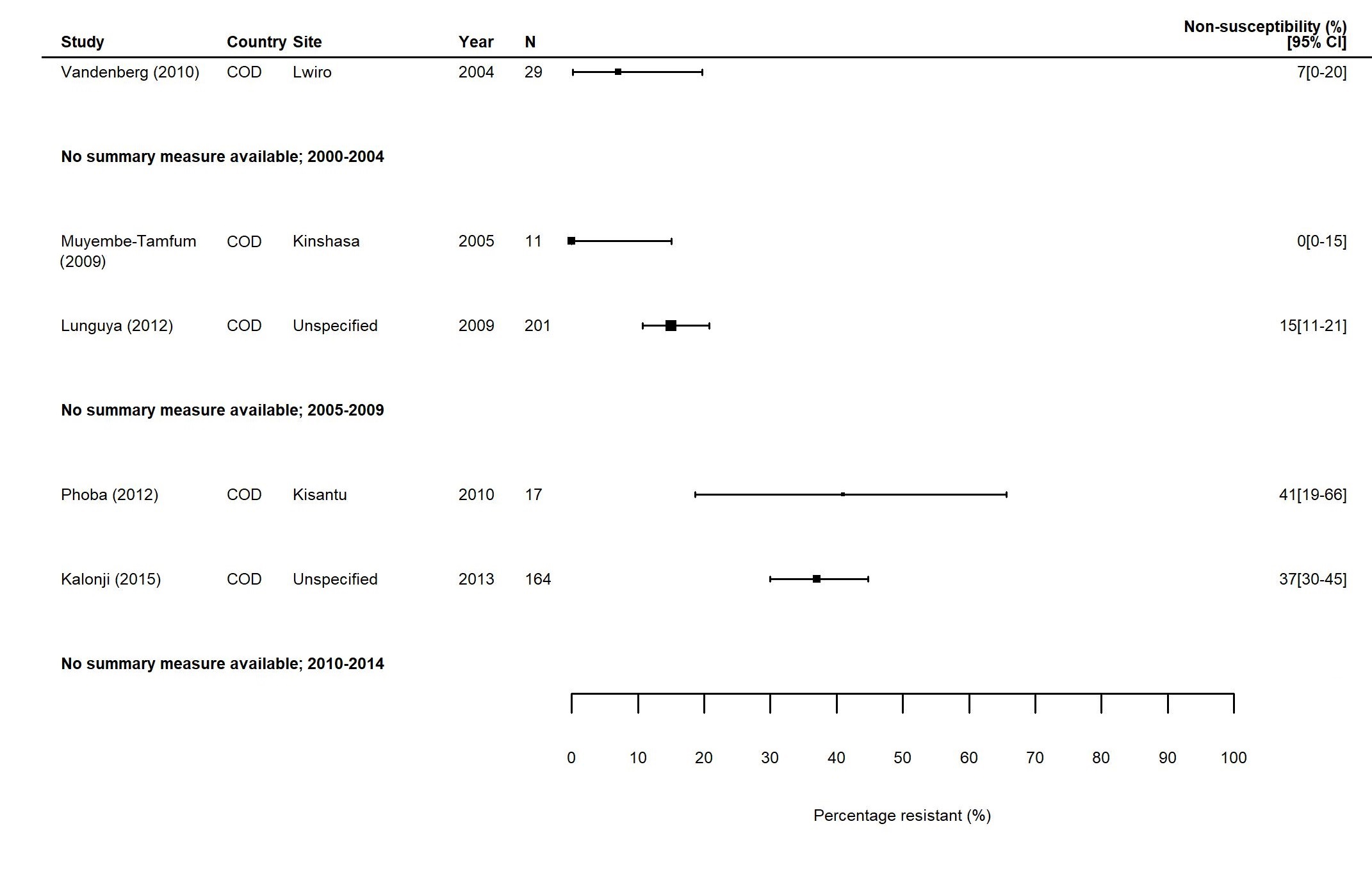
**

(a)

**
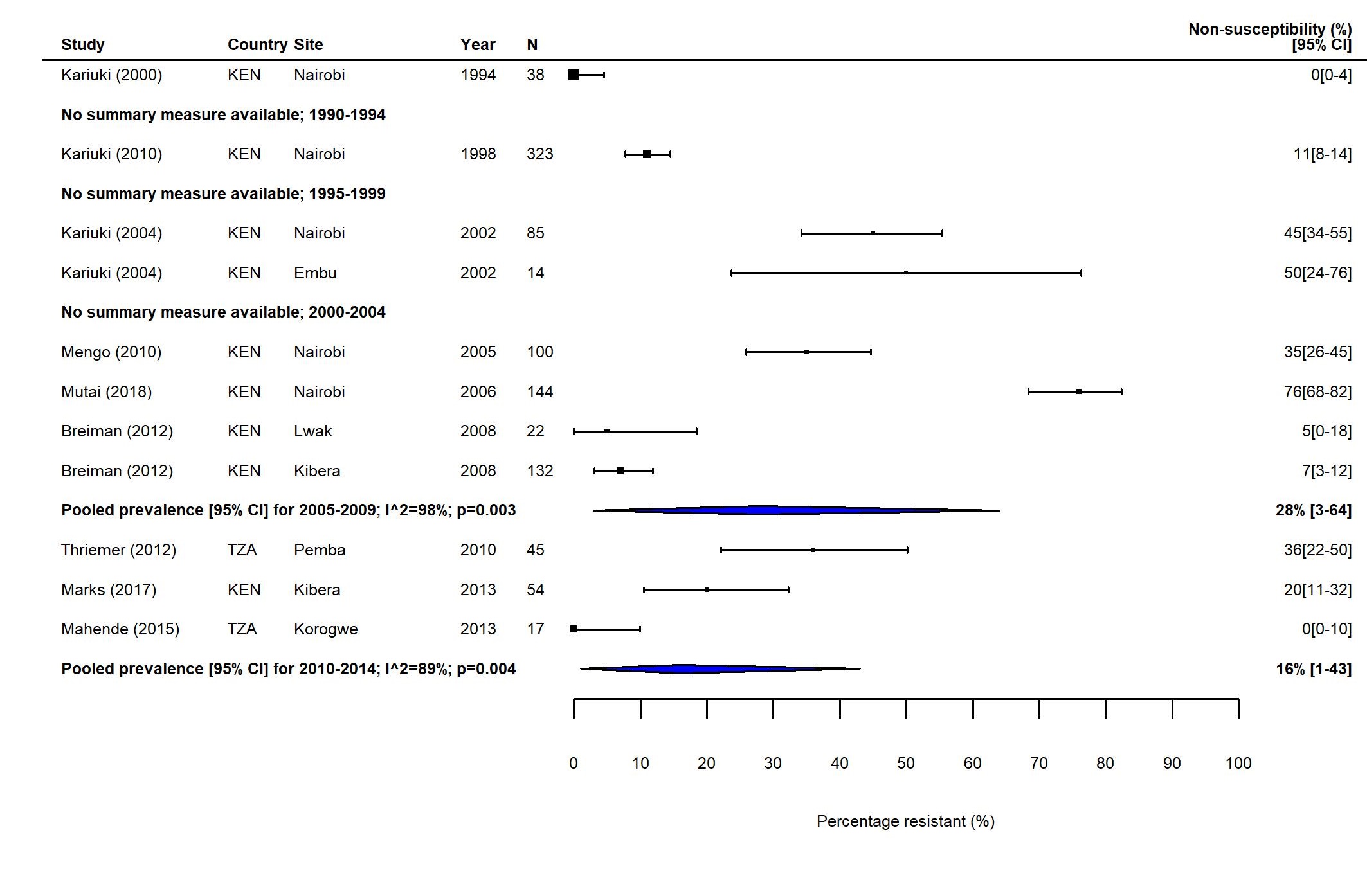
** (b)


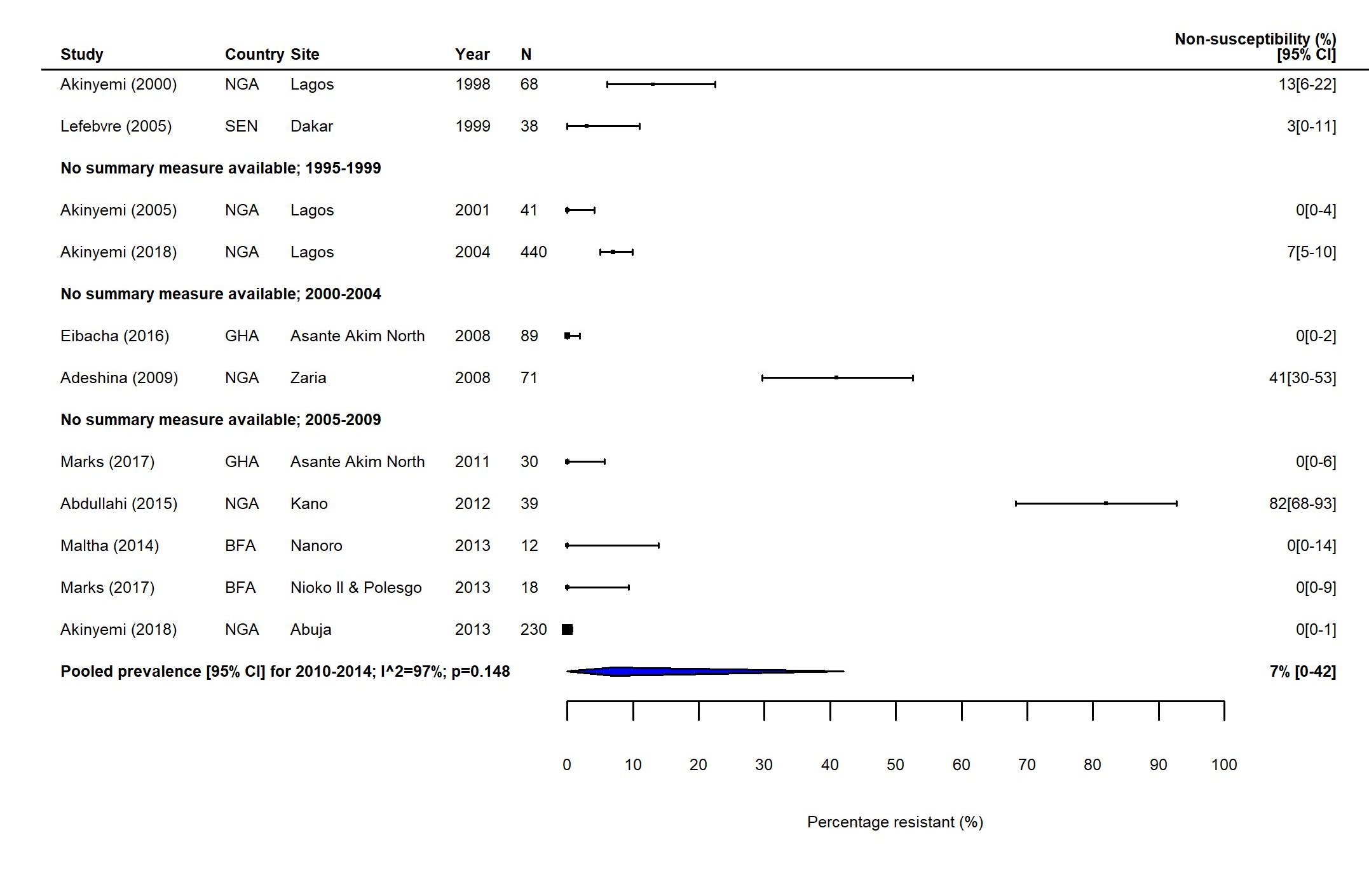


(c)

**Figure S7.** **FQNS *S.* Typhi in North Africa and the Middle East (NAME):** Forest plots illustrating the prevalence of FQNS amongst *S.* Typhi in NAME, grouped by five-year time-periods. Individual study results are displayed with 95% confidence intervals, the pooled prevalence [95%CI] for each subgroup are represented by the blue diamonds.

**
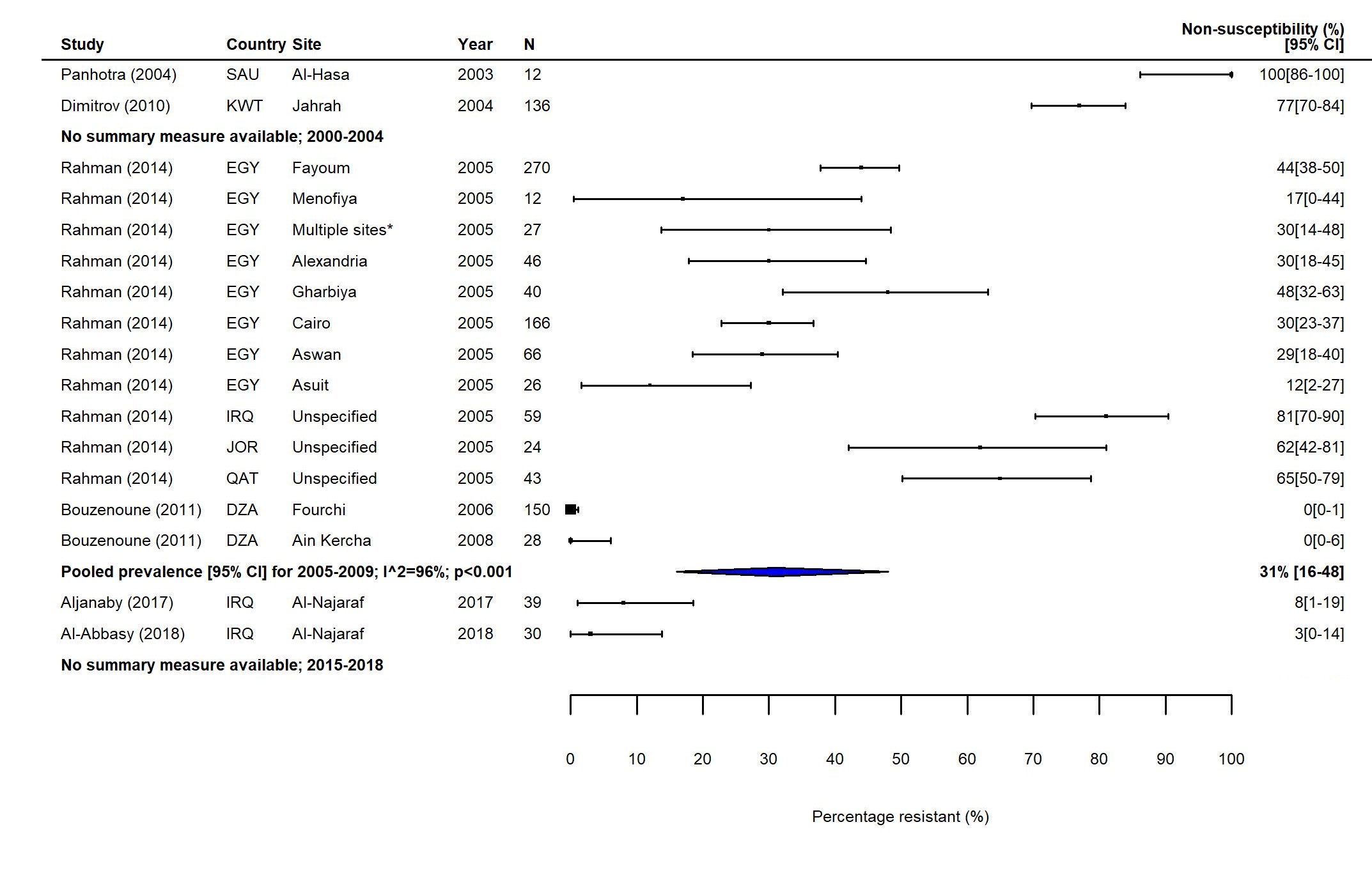
**

**Figure S8.** **FQNS *S.* Typhi in East Asia:** Forest plots illustrating the prevalence of FQNS amongst *S.* Typhi in East Asia, grouped by five-year time-periods. Individual study results are displayed with 95% confidence intervals, the pooled prevalence [95%CI] for each subgroup are represented by the blue diamonds.

**
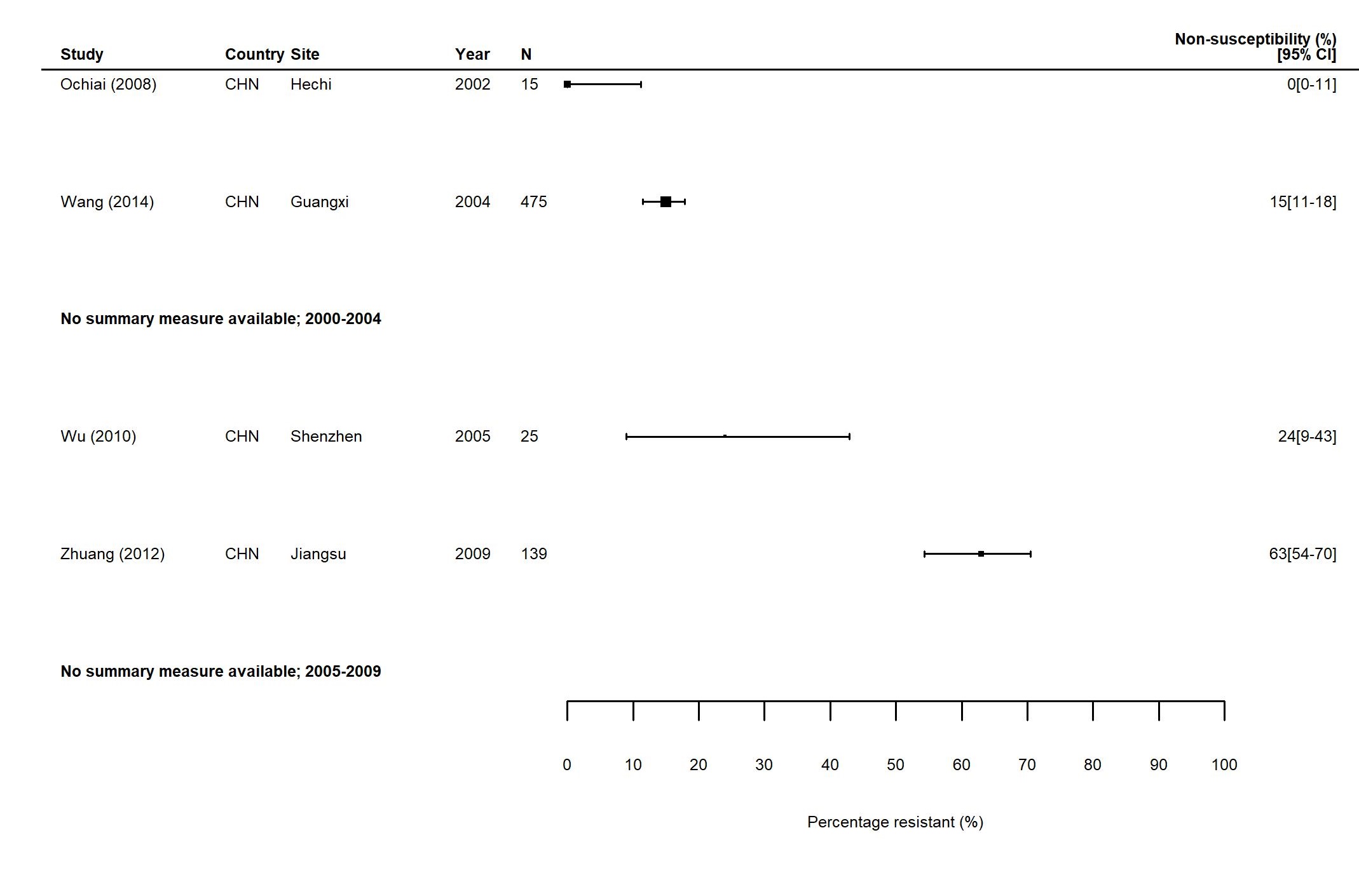
**

**Figure S9. MDR *S.* Paratyphi in South Asia:** Forest plots illustrating the prevalence of MDR amongst *S.* Paratyphi isolates in South Asia, grouped by five-year time-periods. Individual study results are displayed with 95% confidence intervals, the pooled prevalence [95%CI] for each subgroup is represented by the blue diamond: (a) 1990-1999; (b) 2000-2009; (c) 2010-2018. Multidrug resistance is defined as concurrent resistance against ampicillin, chloramphenicol and co-trimoxazole.
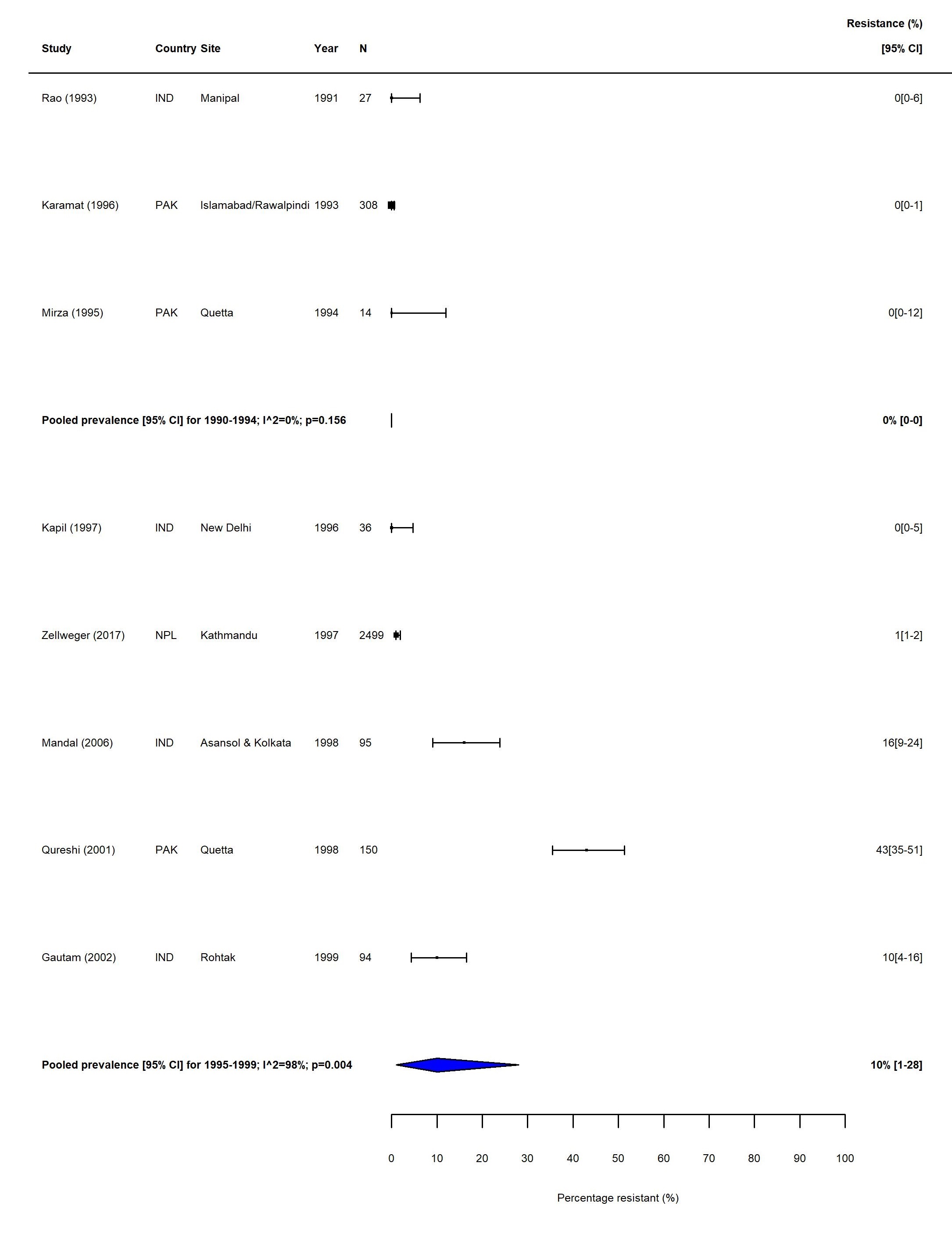
 (a)
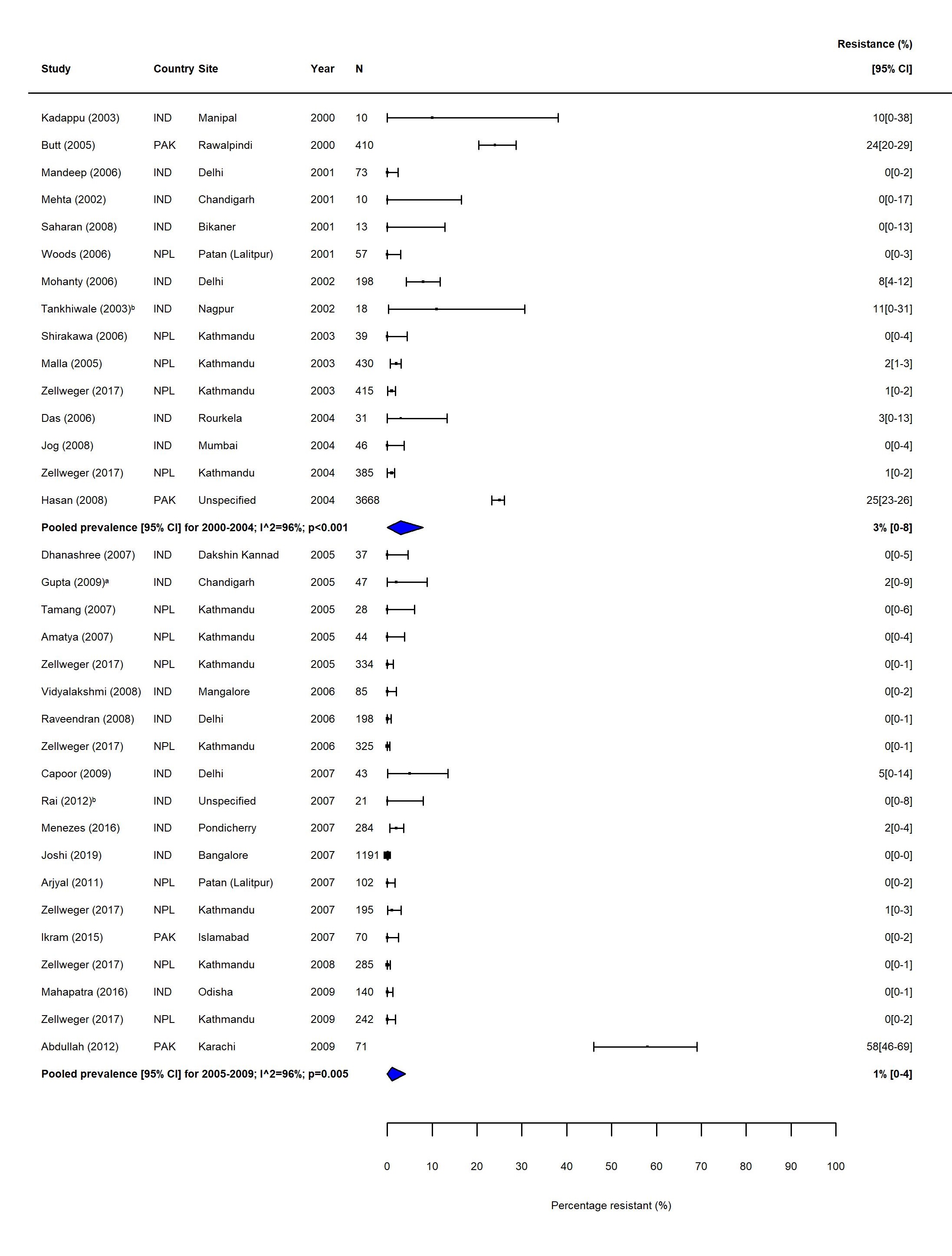


(b)


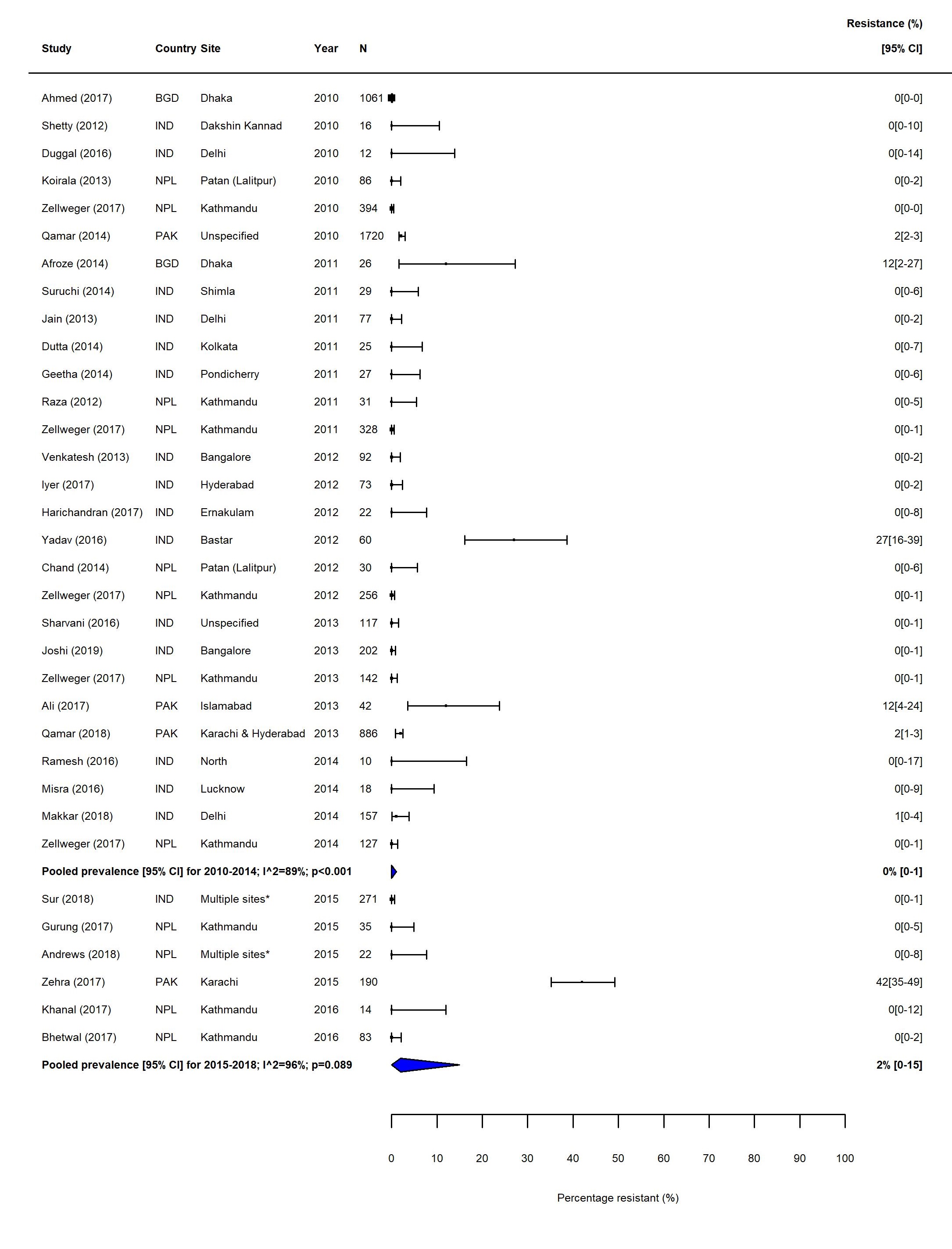


(c)

**Figure S10. MDR *S.* Paratyphi in Southeast Asia:** Forest plots illustrating the prevalence of MDR amongst *S.* Paratyphi isolates in Southeast Asia, grouped by five-year time-periods. Individual study results are displayed with 95% confidence intervals, the pooled prevalence [95%CI] for each subgroup is represented by the blue diamond. Multidrug resistance is defined as concurrent resistance against ampicillin, chloramphenicol and co-trimoxazole.


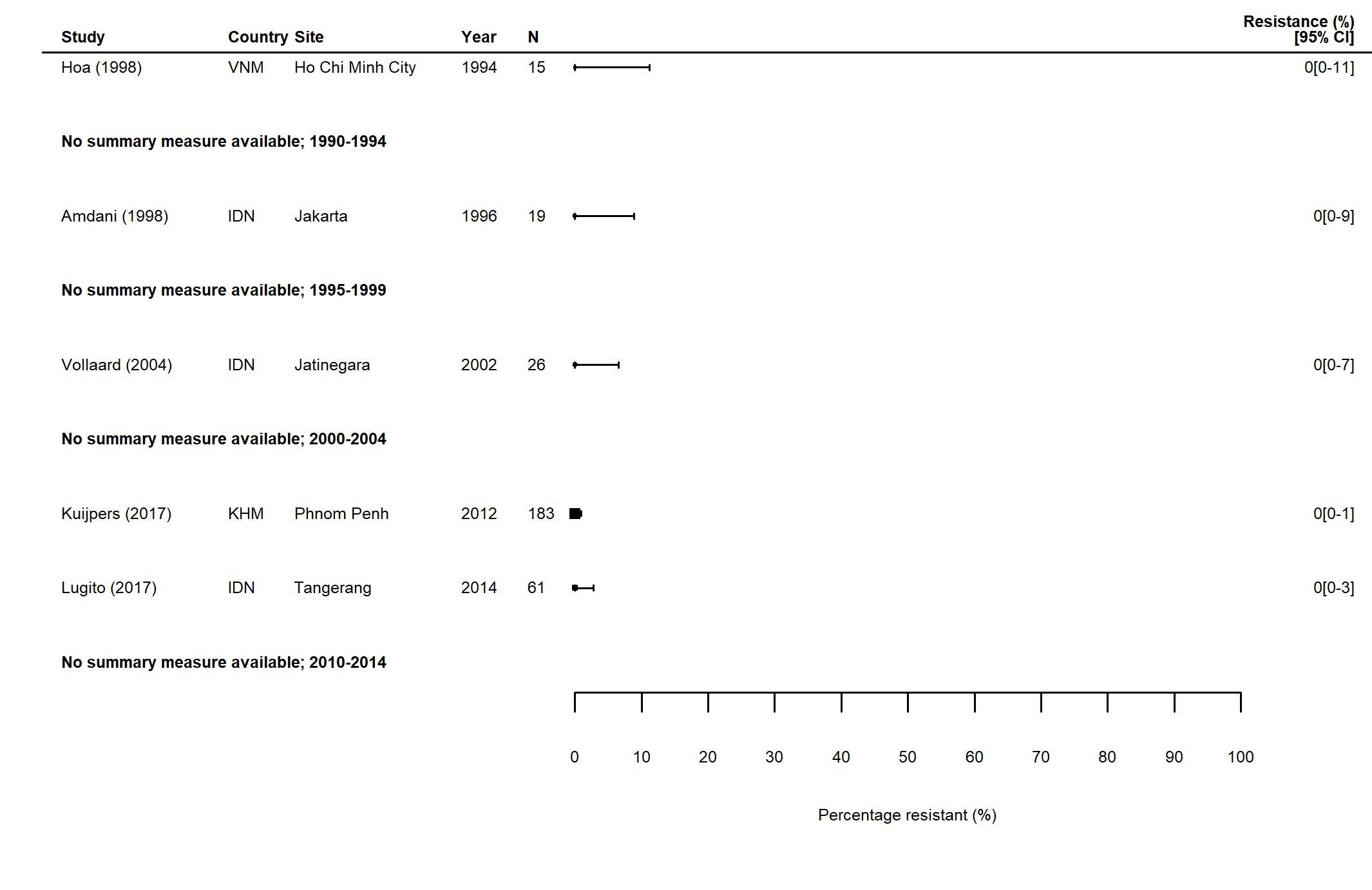


**Figure S11. MDR *S.* Paratyphi in East Asia:** Forest plots illustrating the prevalence of MDR amongst *S.* Paratyphi isolates in East Asia, grouped by five-year time-periods. Individual study results are displayed with 95% confidence intervals, the pooled prevalence [95%CI] for each subgroup is represented by the blue diamond. Multidrug resistance is defined as concurrent resistance against ampicillin, chloramphenicol and co-trimoxazole.

**
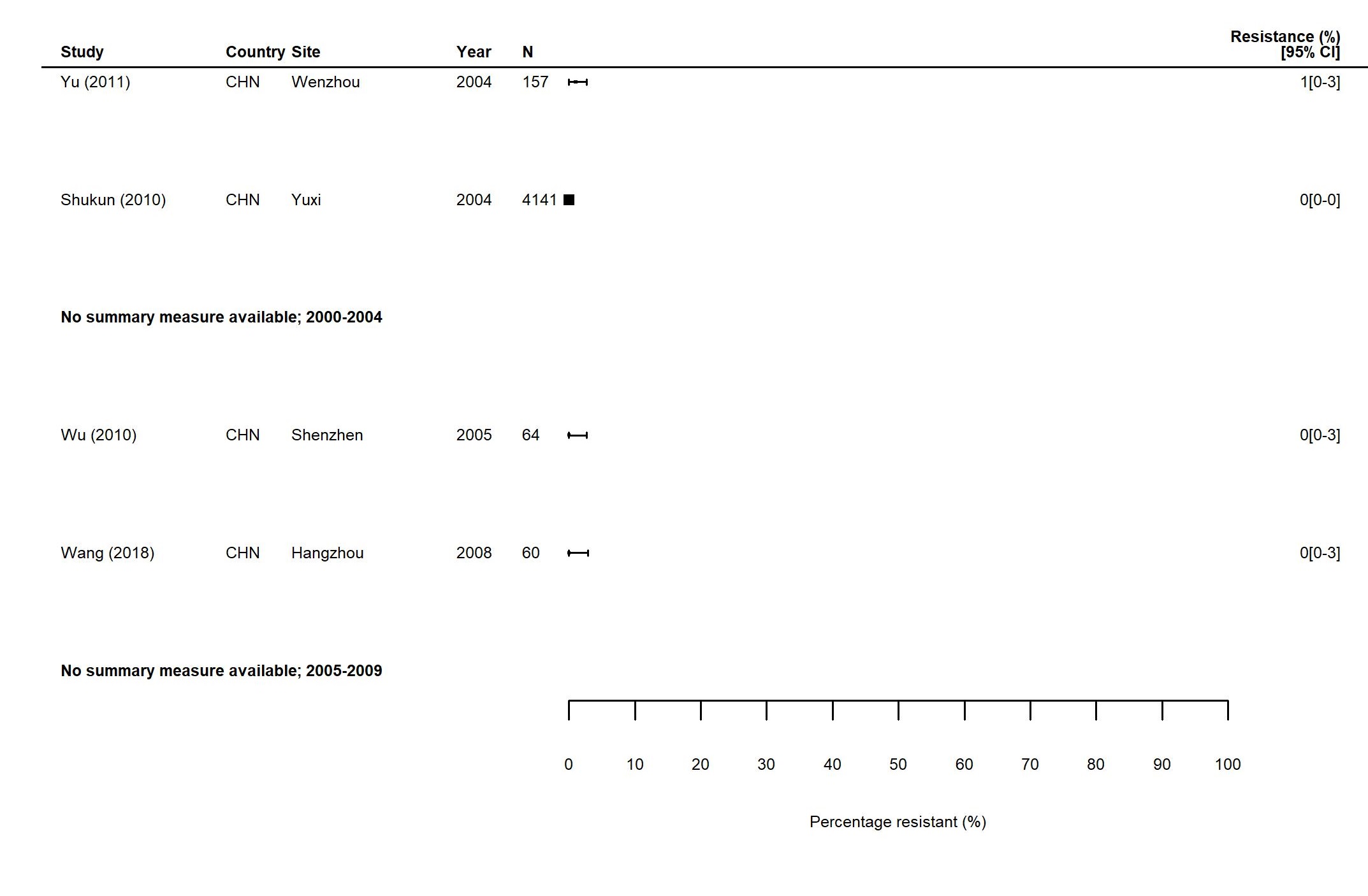
**

**Figure S12. FQNS *S.* Paratyphi in South Asia:** Forest plots illustrating the prevalence of FQNS amongst *S.* Paratyphi in South Asia, grouped by five-year time-periods. Individual study results are displayed with 95% confidence intervals, the pooled prevalence [95%CI] for each subgroup are represented by the blue diamonds.: (a) 1990-2009; (b) 2010-2018.
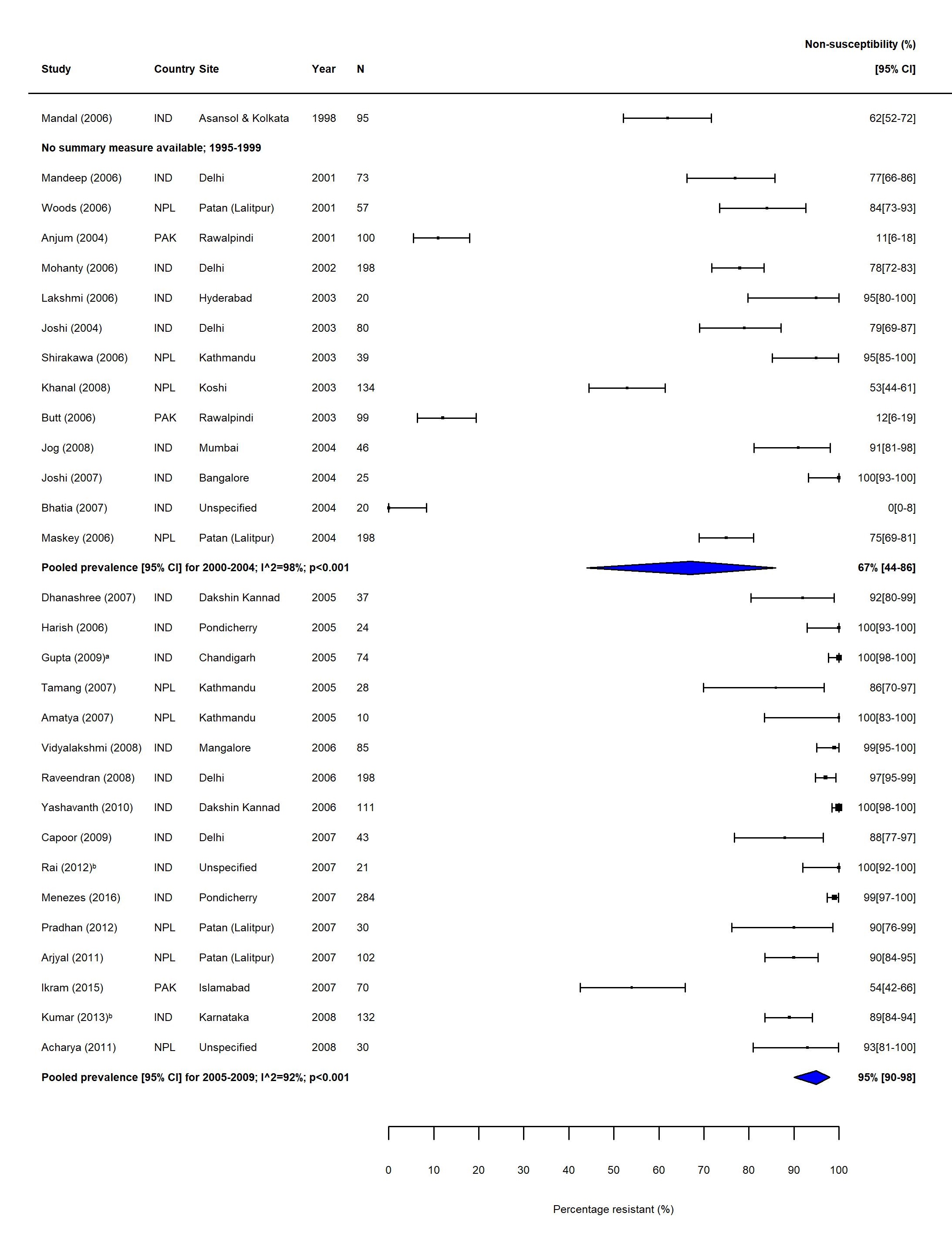


(a)


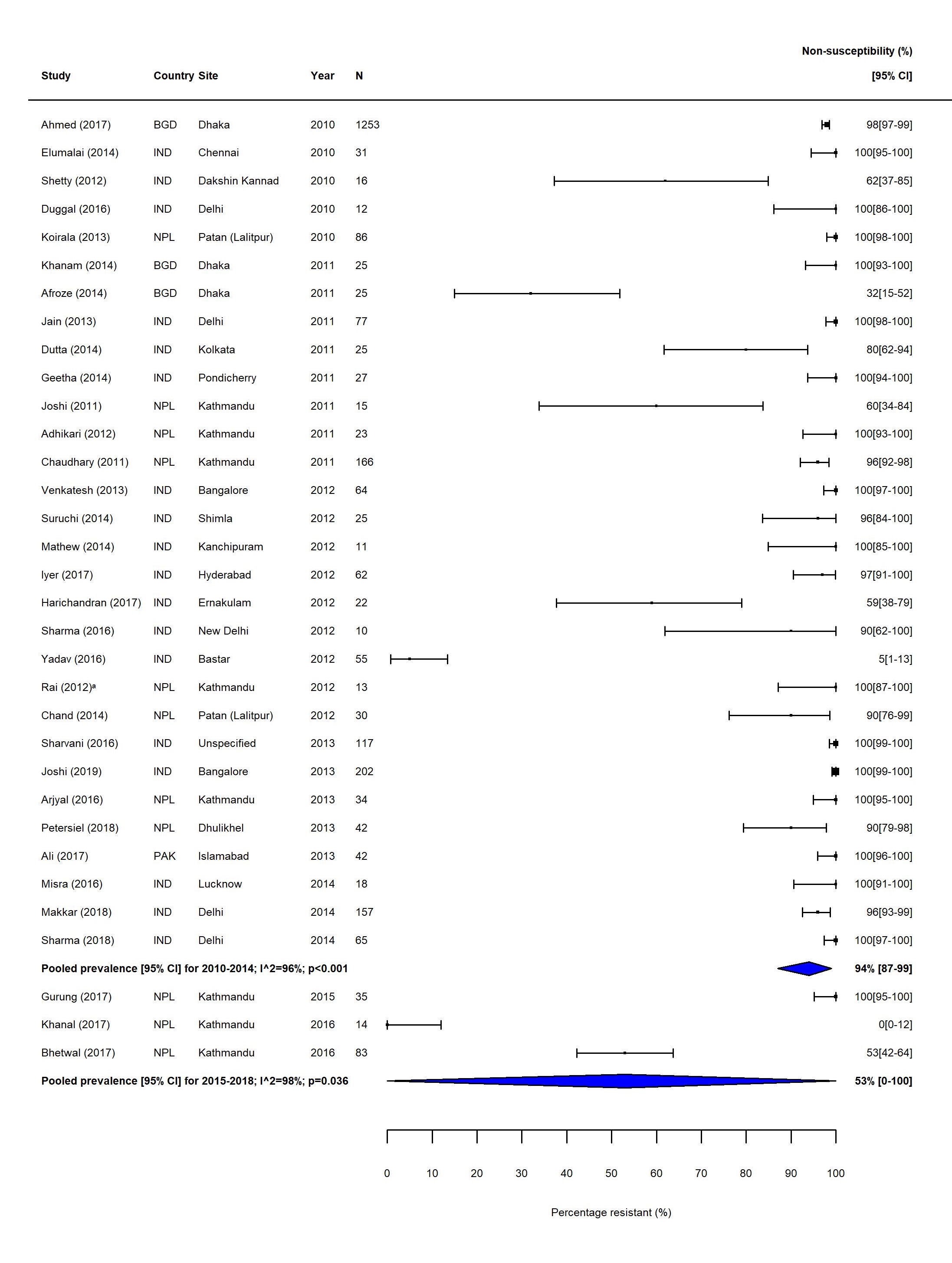


(b)

**Figure S13. FQNS *S.* Paratyphi in Southeast Asia:** Forest plots illustrating the prevalence of FQNS amongst *S.* Paratyphi in Southeast Asia, grouped by five-year time-periods. Individual study results are displayed with 95% confidence intervals, the pooled prevalence [95%CI] for each subgroup are represented by the blue diamonds.
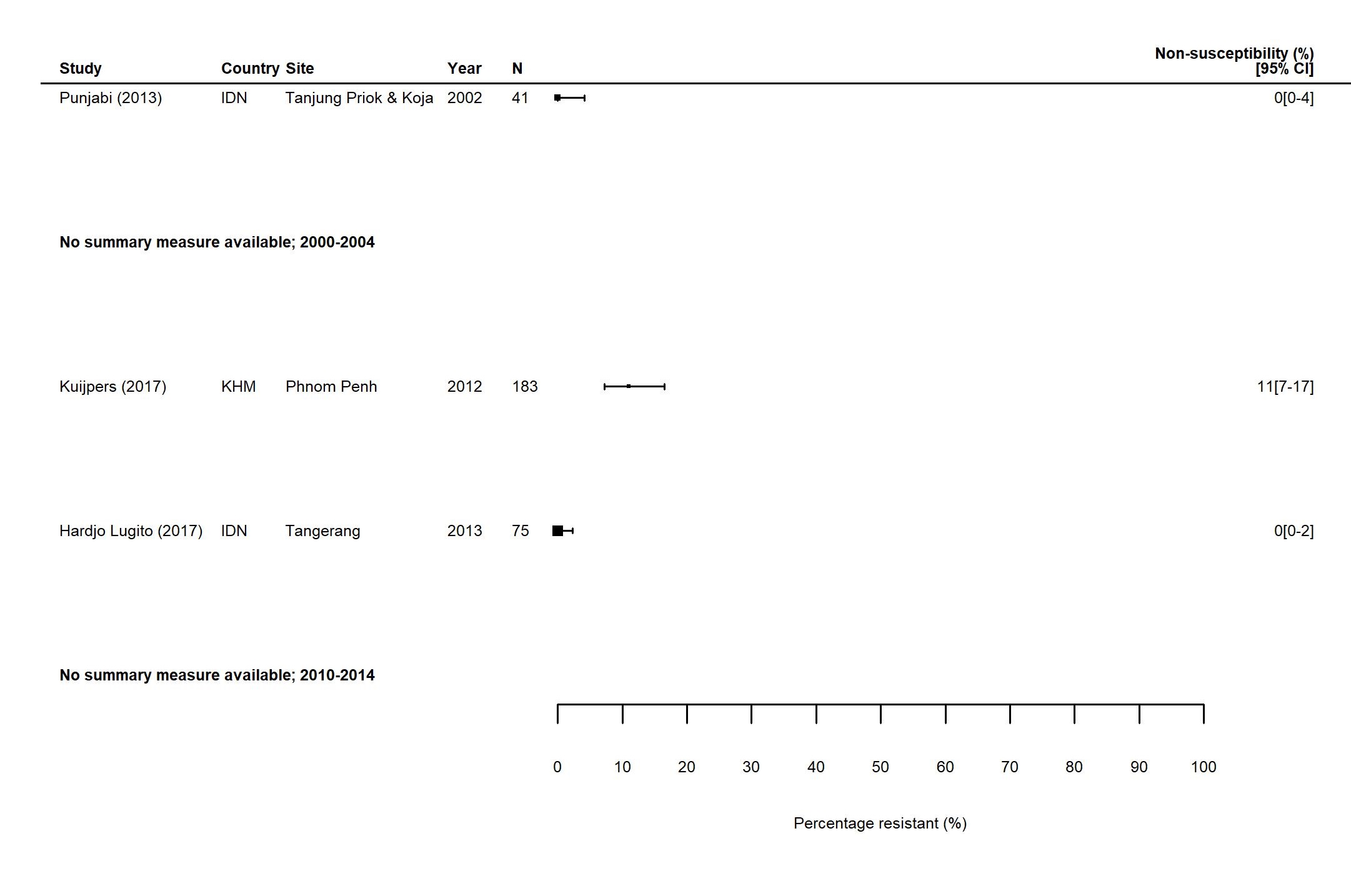


**Figure S14.** **FQNS *S.* Paratyphi in East Asia:** Forest plots illustrating the prevalence of FQNS amongst *S.* Paratyphi in East Asia, grouped by five-year time-periods. Individual study results are displayed with 95% confidence intervals, the pooled prevalence [95%CI] for each subgroup are represented by the blue diamonds.
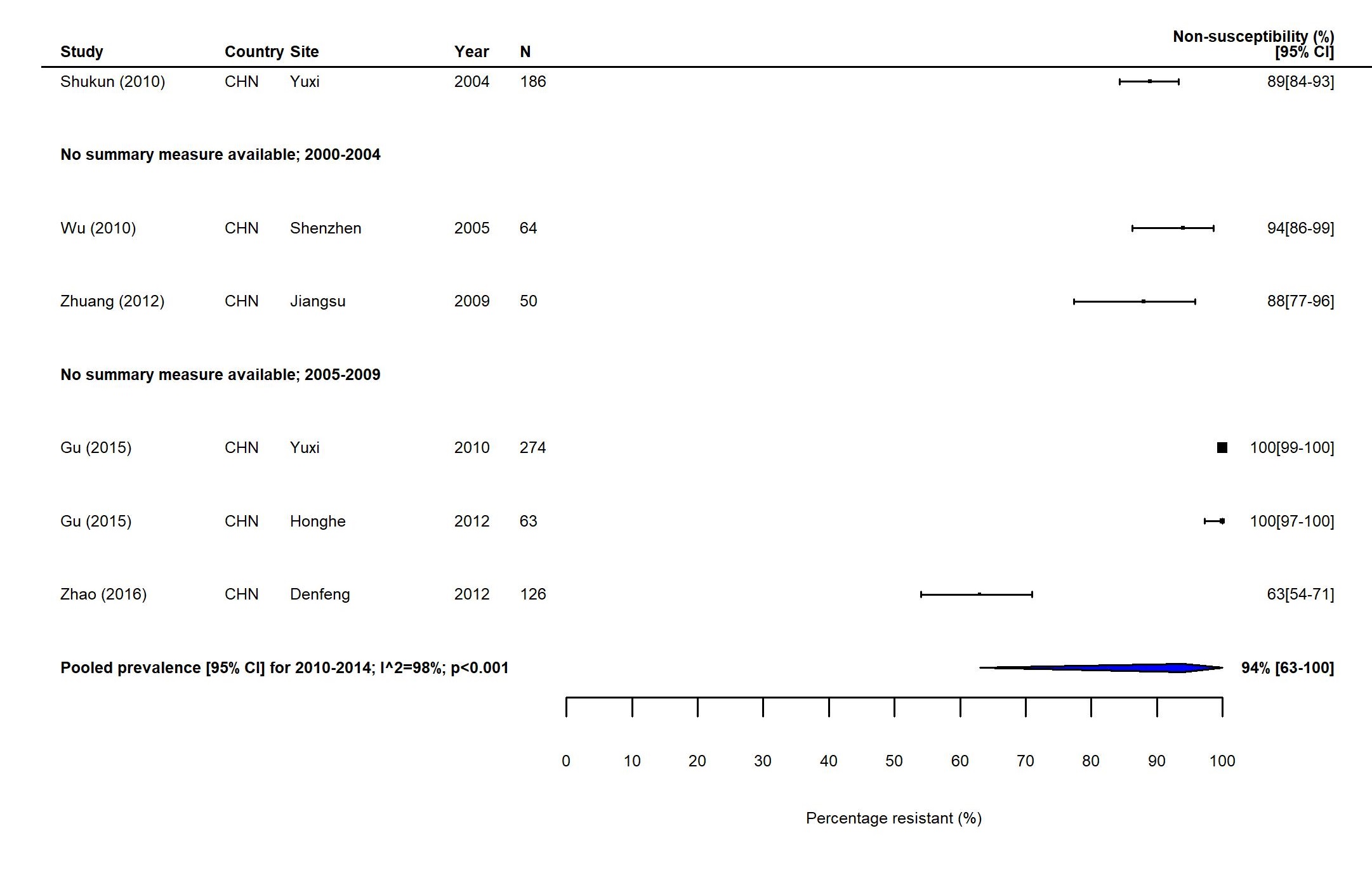

Supplement: Supplementary file 1 — Additional file 1. Supplementary Materials. [file 12916_2019_1443_MOESM1_ESM.docx]
